# Dihydroorotate dehydrogenase inhibition activates STING pathway and pyroptosis to enhance NK cell-dependent tumor immunotherapy

**DOI:** 10.1186/s43556-025-00339-7

**Published:** 2025-10-27

**Authors:** Yongrui Hai, Ruizhuo Lin, Weike Liao, Shuo Fu, Renming Fan, Guiquan Ding, Junyan Zhuang, Bingjie Zhang, Yi Liu, Junke Song, Gaofei Wei

**Affiliations:** 1 Laboratory of Cellular Metabolism and Precision Therapeutics, Institute of Medical Research, Northwestern Polytechnical University, Xi’an, 710072 China; 2 Research & Development Institute of Northwestern Polytechnical University in Shenzhen, Shenzhen, 518057 China; 3 Guizhou Provincial Engineering Technology Research Center for Chemical Drug R&D, Guizhou Medical University, Guiyang, 550004 China; 4 Beijing Key Laboratory of Drug Target Identification and Drug Screening, Institute of Materia Medica, Chinese Academy of Medical Sciences & Peking Union Medical College, Beijing, 100050 China; 5 Department of Medical Oncology, Shaanxi Provincial People’s Hospital, Xi’an 710068, China

**Keywords:** Pyrimidine metabolism, DHODH, NK cells, Pyroptosis, CGAS-STING pathway

## Abstract

Cancer cells rely heavily on de novo pyrimidine synthesis. Inhibiting pyrimidine metabolism directly suppresses tumor growth and fosters immune activation within the tumor microenvironment. Dihydroorotate dehydrogenase (DHODH) is a key enzyme in the de novo pyrimidine synthesis pathway. Inhibiting DHODH can reverse immune suppression and trigger a mild innate immune response. However, the impact of DHODH inhibition on natural killer (NK) cells remains to be explored. In this study, we found that DHODH inhibition promoted NK cell infiltration into tumors efficiently. Mechanistically, DHODH suppression induced mitochondrial oxidative stress, leading to mitochondrial DNA (mtDNA) release into the cytoplasm through voltage-dependent anion channel (VDAC) oligomerization and caspase-3 activation. This subsequently activated the stimulator of interferon gene (STING) pathway, triggered ferroptosis, and induced gasdermin E (GSDME) mediated pyroptosis in cancer cells. These changes collectively facilitated NK cell recruitment. Furthermore, infiltrated NK cells enhanced GSDME-dependent pyroptosis in tumor cells through granzyme release, establishing a positive feedback loop that amplified anti-tumor immunity. Additionally, we developed EA6, a novel DHODH inhibitor that is more effective at promoting NK cell infiltration. In summary, this study reveals that targeting pyrimidine metabolism activates a novel mechanism involving pyroptosis-ferroptosis crosstalk and STING pathway activation to enhance NK cell-mediated immunity. These finding opens new avenues for enhancing the efficacy of targeted nucleotide metabolism in cancer therapy.

**Supplementary Information:**

The online version contains supplementary material available at 10.1186/s43556-025-00339-7.

## Introduction

Abnormally proliferating cancer cells exhibit a heightened demand for energy and biomacromolecules [1]. Nucleotides, the fundamental components of RNA and DNA, play a crucial role in the accelerated nucleotide metabolism that drives tumor cell proliferation, metastasis, and immune evasion [2]. Consequently, targeting nucleotide synthesis remains a cornerstone of contemporary cancer therapy. Traditional nucleotide synthesis inhibitors, such as 5-fluorouracil (5-FU), methotrexate, and cytarabine, are analogs of tumor nucleotide metabolites. Recent research has revealed that nucleotide antimetabolite drugs (NMDs) not only restrain tumor cell proliferation but also hinder immune evasion, providing novel insights into therapeutic approaches aimed at nucleotide metabolism [3].

Pyrimidine nucleotides, which are a key component of nucleotides, are synthesised through two distinct pathways: the salvage pathway and the de novo pathway. Terminally differentiated or resting cells primarily rely on the salvage pathway to maintain nucleotide homeostasis, whereas rapidly proliferating cells, particularly cancer cells, depend heavily on de novo pyrimidine synthesis [4]. Therefore, effectively suppressing de novo pyrimidine synthesis has proven instrumental in impeding tumour progression. Dihydroorotate dehydrogenase (DHODH) is a critical rate-limiting enzyme in the de novo pyrimidine synthesis pathway that catalyses the conversion of dihydroorotate to orotate. DHODH is unique among pyrimidine synthetases in that it is located on the outer surface of the inner mitochondrial membrane and is linked to the mitochondrial electron transport chain [5]. Inhibiting DHODH has been shown to effectively suppress the growth of melanoma [6], glioma [7], small cell lung cancer [8], and various solid tumors, while also overcoming differentiation blockade in acute myeloid leukaemia [9]. These findings emphasise the potential of DHODH as a promising pharmaceutical target in cancer treatment.

It is notable that pyrimidine metabolism significantly influences the immune response within the tumour microenvironment. Disruptions to the levels of purines and pyrimidines can lead to increased genomic instability, thereby enhancing immunogenicity [3]. Hans-Georg Sprenger et al. have suggested that imbalanced pyrimidine levels in cells can trigger innate immune responses [10]. Conversely, pyrimidine metabolism is closely associated with anti-tumor effects of various immune cells. DHODH inhibition restrains cell proliferation without affecting T cell functionality [11]. It also promotes the differentiation of myeloid-derived suppressor cells (MDSC), which can reverse immunosuppressive microenvironments [12]. Furthermore, pyrimidines released from macrophages can limit the efficacy of gemcitabine therapy in pancreatic cancer [13], suggesting that blocking the uptake of pyrimidines by cancer cells could enhance the therapeutic efficiency of chemotherapeutic agents. Therefore, disrupting pyrimidine balance could enhance anti-tumour immunity.

Natural killer (NK) cells play a pivotal role in the innate surveillance of cancer, identifying and attacking stressed cells, particularly those with suppressed major histocompatibility complex class I (MHC-I) expression [14, 15]. As MHC-unrestricted NK cells complement T cell-mediated tumor immunity, the impact of pyrimidine imbalance on NK cell activation is a significant area of interest.

In this study, we observed a substantial increase in tumor-infiltrating NK cells following DHODH inhibition. Suppressing DHODH led to pyroptosis in cancer cells and activated the cyclic GMP-AMP synthase (cGAS)-stimulator of interferon genes (STING) pathway, forming the basis for NK cell-induced anti-tumor immune responses. Additionally, we designed and synthesised a series of DHODH inhibitors, with EA6 exhibiting the greatest activity. EA6 is a markedly more efficient DHODH inhibitor, with approximately 10 times the activity of the classical DHODH inhibitor brequniar (BRQ), and triggers NK infiltration more efficiently. In summary, this study highlights that modulation of pyrimidine metabolism can effectively trigger anti-tumor immune responses, with a particular focus on NK cells. This finding opens new avenues for enhancing the efficacy of targeted nucleotide metabolism in cancer therapy.

## Results

### DHODH inhibition suppresses tumor growth and modulates immune cell dynamics

**Fig. 1:**
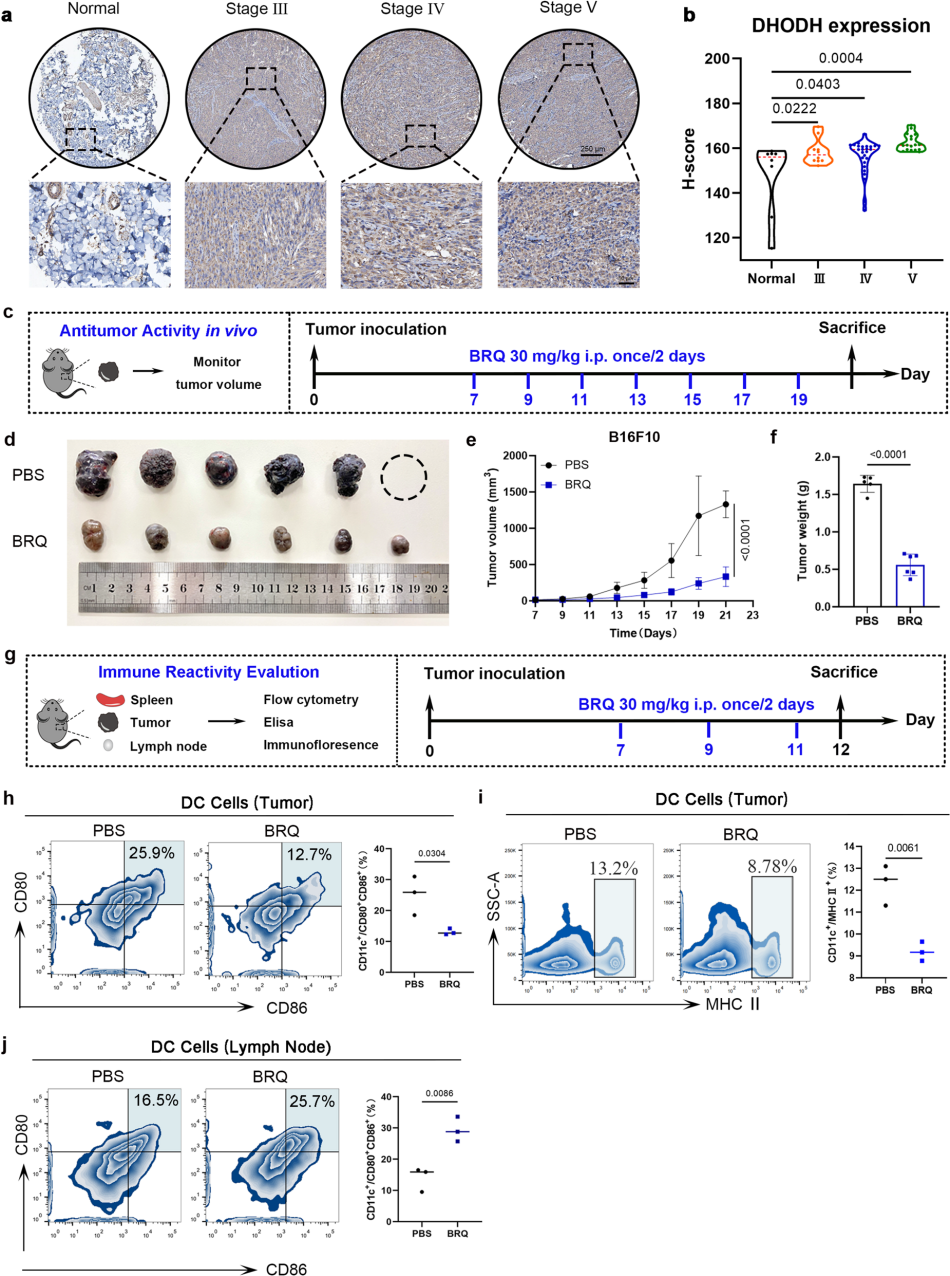
BRQ restrains melanoma growth. **a** Representative images of immunohistochemical staining of DHODH. Melanoma staging by Clark classification. **b** Immunohistochemistry score of DHODH in normal tissues (*n* = 8) and different grades tissue samples of melanoma (grade Ⅲ: *n* = 10; grade Ⅳ: *n* = 26; grade Ⅴ: *n* = 17). **c** Experiment scheme for BRQ therapy of the B16F10 tumor bearing C57BL/6 female mouse model. **d** Images of isolated tumors at the end of the experiment (*n* = 6). **e** Tumor growth in the indicted groups of mice (*n* = 6). **f** The average tumor weight at the end of the experiment (*n* = 6). **g** Immune experiment scheme of the B16F10 tumor bearing C57BL/6 female mouse model. **h** Representative flow cytometry images and quantitative graph of DC cells (CD11c^+^ CD80^+^ CD86^+^) in tumor (*n* = 3). **i** Representative flow cytometry images and quantitative graph of DC cells (MHC Ⅱ.^+^) in tumor (*n* = 3). **j** Representative flow cytometry images and quantitative graph of DC cells in lymph nodes (*n* = 3)

To define the role of DHODH in melanoma, we first performed a tissue microarray analysis. Immunohistochemistry confirmed a significant upregulation of DHODH in melanoma tissues compared to normal skin, with elevated expression maintained across different disease stages (Fig. 1a-b and Fig. S1a). Notably, inhibition of DHODH resulted in a significant regression of melanoma growth [16]. These compelling findings underscore the potential of DHODH as a promising therapeutic target for melanoma treatment.

We next evaluated the therapeutic potential of BRQ, a known DHODH inhibitor, in a B16F10 melanoma mouse model (Fig. 1c). BRQ treatment significantly suppressed tumor growth (Fig. 1d-f), confirming its anti-tumor efficacy. To investigate the immune mechanisms underlying this effect, we analyzed immune cell infiltration following BRQ treatment (Fig. 1g). While we observed a redistribution of mature dendritic cells from tumors to lymph nodes (Fig. 1h-j, S1b-d), the migration of mature DCs from the tumor to the lymph nodes can activate T cells [17], and macrophages are also an important component of immune system, tumor-infiltrating macrophages affect tumor cells proliferation, invasion and metastasis [18]. However, no significant changes were detected in CD8^+^T cells, CD4^+^T cells, or macrophage populations in tumors, spleens, or lymph nodes (Fig. S1e-h). This pattern suggested that BRQ's anti-tumor activity might involve other immune components beyond conventional T-cell responses.

### BRQ enhances anti-tumor immunity through NK cell recruitment and activation

**Fig. 2:**
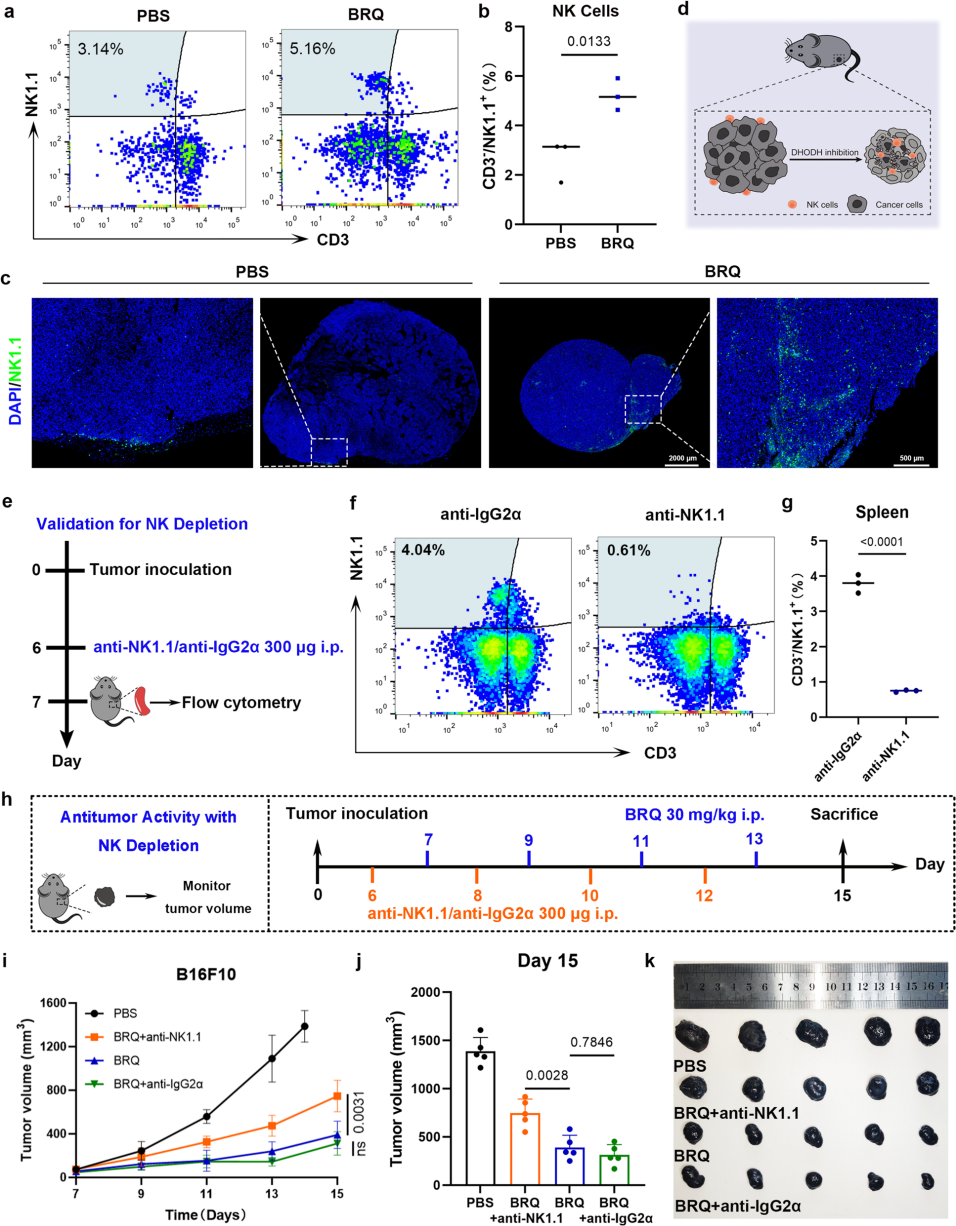
BRQ triggers NK cells infiltration in tumor. **a-b** Representative flow cytometry images and quantitative graph of NK cells in tumor (*n* = 3). **c** Representative immunofluorescence images of tumor-infiltrating NK cells. **d** Schematic representation of BRQ induced anti-tumor immune response. **e** Schematic representation of NK depletion experiment. **f-g** Representative flow cytometry images and quantification of NK cells in spleen after treatment with anti-IgG2α or anti-NK1.1 antibody (*n* = 3). **h** Experiment scheme for BRQ therapy with NK depletion. **i** Tumor growth curves in the various groups of mice (*n* = 5). **j** Average tumor volume at Day 15 of each group (*n* = 5). **k** Images of isolated tumors at the end of experiment in NK depletion experiment (*n* = 5)

NK cells are critical for broad-spectrum anti-tumor effects, as they directly recognize and attack tumor cells without MHC restriction. This makes them particularly effective against tumors with downregulated or lost MHC-I expression, such as malignant melanomas [15, 19]. To determine the role of NK cells in BRQ-mediated tumor suppression, we analyzed NK cells infiltration using flow cytometry and Immunofluorescence. The results revealed significantly increased NK cell infiltration in tumors following BRQ treatment (Fig. 2a-b). While NK cells in control groups were predominantly localized at the tumor periphery, BRQ treatment promoted their deep penetration into tumor cores (Fig. 2c). This enhanced infiltration was accompanied by upregulated expression of pro-inflammatory cytokines (IFN-γ and IL-1β) and downregulation of the NK cell suppressor TGF-β (Fig. S1i-k) [20], indicating BRQ remodels the tumor microenvironment to support NK cell activity (Fig. 2d).

To assess the critical role of NK cells in BRQ-mediated anti-tumor effects, we performed antibody-mediated depletion experiments (Fig. 2e). NK cell depletion was confirmed by splenic flow cytometry (Fig. 2f-g). Notably, NK cell ablation significantly attenuated BRQ's anti-tumor efficacy, while control antibody treatment showed no effect (Fig. 2h-k). These results establish that BRQ-mediated tumor suppression substantially depends on NK cells as crucial effector components of the anti-tumor immune response.

### Transcriptomic analysis reveals immune activation mechanisms underlying BRQ treatment

To investigate the mechanism by which BRQ enhances NK cell infiltration, we performed RNA sequencing analysis. Transcriptomic profiling demonstrated high reproducibility within experimental groups and clear separation between BRQ-treated and control samples (Fig. S1a-b). BRQ treatment significantly altered gene expression, with 2,460 genes upregulated and 3,518 genes downregulated (Fig. S1c-d).

**Fig. 3:**
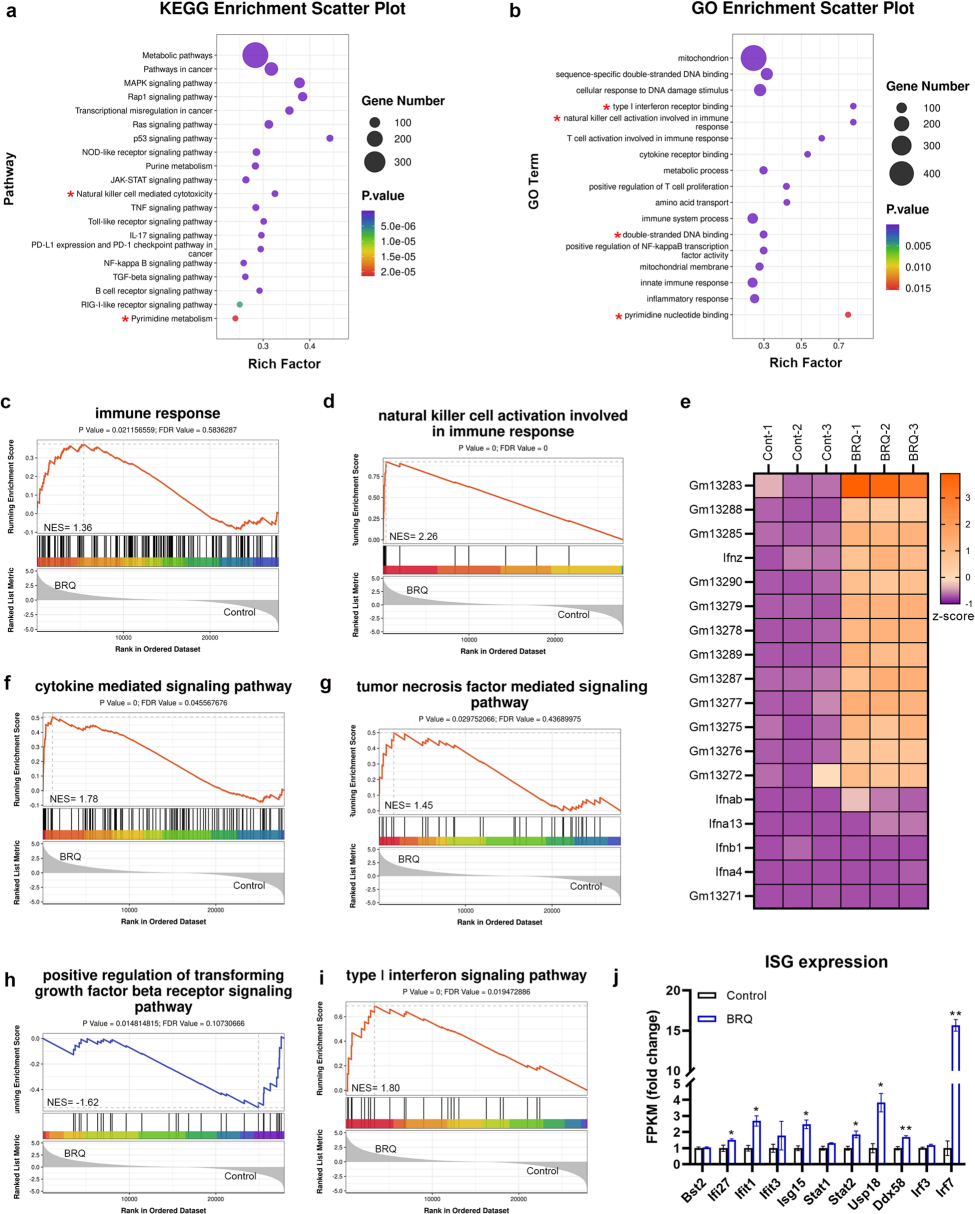
RNA-Seq analysis of B16F10 cells treated with BRQ. **a** KEGG enrichment analysis of DEGs after BRQ treatment. **b** GO enrichment analysis of DEGs after BRQ treatment. **c** GSEA analysis of immune response. **d** GSEA analysis of NK cells activation. **e** Specific genes related to NK cells activation. **f** GSEA analysis of cytokine mediated signaling pathway. **g** GSEA analysis of TNF mediated signaling pathway. **h** GSEA analysis of TGF-β signaling pathway. **i** GSEA analysis of IFN-Ⅰ signaling pathway. **j** ISG expression between the BRQ group and the control group

Kyoto encyclopedia of genes and genomes (KEGG) enrichment analysis revealed that differentially expressed genes (DEGs) were significantly associated with pyrimidine metabolism, reflecting BRQ's inhibitory effect on de novo pyrimidine synthesis (Fig. 3a). Importantly, DEGs were enriched in pathways associated with immune responses, including the Natural killer cell mediated cytotoxicity, NOD-like receptor signaling, JAK-STAT signaling and cytokine pathways (TNF, IL-17, TGF-β). Gene ontology (GO) analysis further confirmed alterations in mitochondrial function and double-stranded DNA binding (Fig. 3b).

Gene set enrichment analysis (GSEA) demonstrated significant upregulation of immune system activation pathways, particularly those involved in NK cell function and cytokine signaling (Fig. 3c-f). BRQ treatment specifically enhanced TNF-α signaling while suppressing TGF-β pathway activity (Fig. 3g-h). Crucially, we observed activation of the type I interferon (IFN-Ⅰ) signaling pathway, evidenced by upregulation of interferon-stimulated genes (ISGs) including IFIT1, ISG15, STAT2, and IRF7 (Fig. 3i-j) [21, 22].

Considering the fundamental role of mitochondria in innate immunity [22] and the potential of cellular pyrimidine imbalance to trigger mitochondrial DNA-dependent innate immunity [10], our RNA-seq results support the hypothesis that BRQ-induced pyrimidine imbalance may contribute to NK cells infiltration into tumors by activating the STING signaling pathway.

### BRQ activates cGAS-STING pathway to enhance the anti-tumor immunity of NK cells

The STING signaling pathway serves as a pivotal link bridging innate and adaptive immunity [23, 24]. Stimulation of the STING pathway can activate NK cells, promising effectors for anti-tumor responses [25, 26]. STING can be activated in response to DNA damage, with the recognition of intracellular free DNA by cGAS being the initial step. Notably, inhibition of de novo pyrimidine synthesis efficiently induces DNA damage and disrupts the pyrimidine balance [4]. Consequently, we explored the effect of BRQ on activating the STING pathway.

**Fig. 4:**
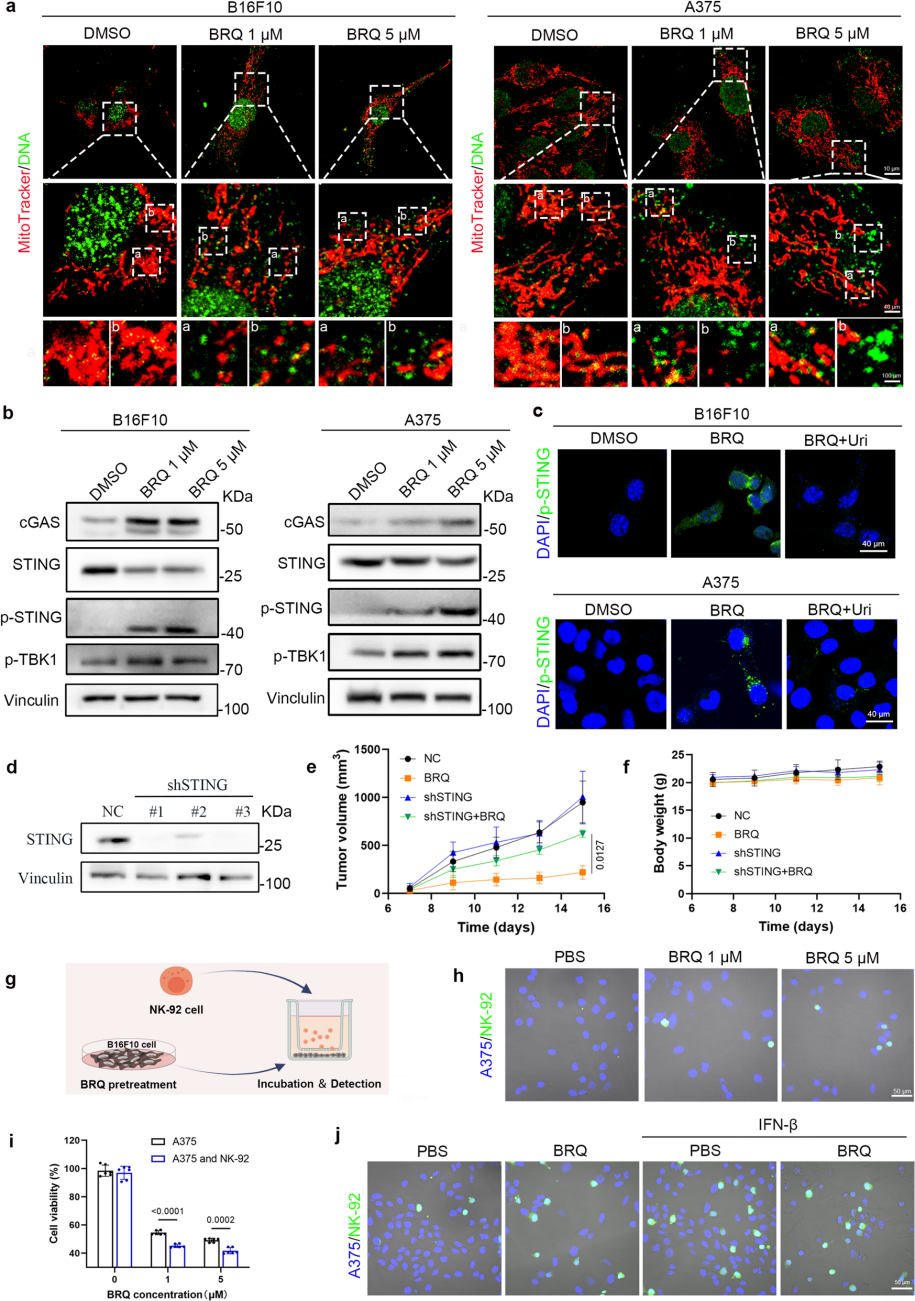
BRQ activates cGAS-STING pathway to enhance the anti-tumor immunity of NK cells.** a** CLSM images of mtDNA released from mitochondrial in B16F10 and A375 (Red: mitochondrial; green: DNA). **b** Western blot analysis of protein involved in cGAS-STING pathway. **c** p-STING expression in B16F10 and A375 cells with DMSO, BRQ (5 μM) or BRQ (5 μM) plus uridine (100 μM). **d** Verification of STING protein knockdown efficiency. **e** B16F10 tumor growth in mice treated with PBS, BRQ (30 mg/kg, i.p.) (*n* = 5). **f** Body weight of mice (*n* = 5). **g** In vitro experiments for NK cells infiltration after treatment: A375 cells were pretreated with BRQ for 24 h and then co-cultured with NK-92 cells in a transwell system. **h** Images of NK-92 contact with pretreated A375 cells as shown in **g** (green: NK-92 label with CFSE, blue: A375 label with DAPI). **i** Cell viability of A375 cells cocultured or not with NK-92 cells after pretreated with BRQ. The A375 alone or co-cultured with NK-92 cells without drug treatment were used as control, respectively (*n* = 6). **j** Images of NK-92 contact with A375 cells. A375 cells were pretreated with BRQ and then treated with or without IFN-β as shown in **g** (green: NK-92 label with CFSE, blue: A375 label with DAPI)

Immunofluorescence analysis revealed dose-dependent mitochondrial DNA (mtDNA) release into the cytosol following BRQ treatment in both B16F10 and A375 cells (Fig. 4a). This was accompanied by significant upregulation of key cGAS-STING pathway components, including cGAS, p-STING, and p-TBK1 (Fig. 4b, S3a-b). The activation of cGAS-STING signaling pathway leads to the induction of IFN-I, which paly critical roles in NK cell biology, including maturation, homeostasis, and activation [27]. Consistent with STING pathway activation, BRQ treatment induced dose-dependent IFN-β production (Fig. S3c).

Uridine serves as the precursor of pyrimidine nucleotides, and its exogenous supplementation has been used to counterbalance the depletion of endogenous uridine resulting from DHODH inhibition. To investigate whether the activation of the cGAS-STING pathway arises from a deficiency in pyrimidine synthesis, we concurrently treated cells with uridine and BRQ. The specificity of this response was confirmed through uridine supplementation, which reversed BRQ-induced STING phosphorylation (Fig. 4c), establishing the dependence on pyrimidine pool depletion.

To confirm that BRQ activates the cGAS-STING signaling pathway in vivo, we analyzed tumor tissues to assess p-STING and IFN-β expression levels. As shown in Fig. S3d–g, p-STING and IFN-β expression increased in tumors treated with BRQ. These results indicate that BRQ activates the cGAS-STING signaling pathway and induces the expression of IFN-Ⅰ, which are important for NK cells induced anti-tumor immunity. Crucially, STING knockdown significantly attenuated BRQ's anti-tumor efficacy (Fig. 4d-f, S3h), confirming the pathway's essential role. In addition, We excluded involvement of alternative DNA sensors absent in melanoma 2 (AIM2) and heterogeneous nuclear ribonucleoprotein A2B1 (hnRNPA2B1) [28, 29], as their expression remained unchanged following BRQ treatment (Fig. S3i-j).

To investigate whether BRQ enhances NK cells cytotoxicity against cancer cells in vitro, we implemented a transwell co-culture system with NK-92 cells in the upper chamber and A375 cells in the bottom chamber to investigate whether BRQ facilitate NK cells infiltration (Fig. 4g). The results demonstrated that BRQ pretreatment enhanced NK-92 cell recognition and cytotoxicity against A375 cells (Fig. 4h-i). Considering the pivotal role of IFN-β in regulating of NK cells numbers, activation, and anti-tumor activity [27], we supplemented exogenous IFN-β into the bottom chamber to investigate NK cells infiltration. Representative images showed that IFN-β facilitated NK cells infiltration, similar to the effect observed with BRQ treatment, which indicated that BRQ-induced IFN-β release is critical for NK cells infiltration (Fig. 4j). It is well established that activation of the STING signalling pathway directly enhances NK cell activity [30]. Importantly, BRQ exhibited no significant cytotoxicity toward NK cells while activating their STING pathway (Fig. S3k-l), suggesting dual enhancement of both tumor immunogenicity and NK cell function.

In summary, these compelling results clearly demonstrate that BRQ exerts potent activation of the STING signaling pathway both in vitro and in vivo, consequently mediating an effective anti-tumor immune response which rely on NK cells.

### NK cell-mediated positive feedback loop amplifies GSDME-dependent pyroptosis in tumor therapy

**Fig. 5:**
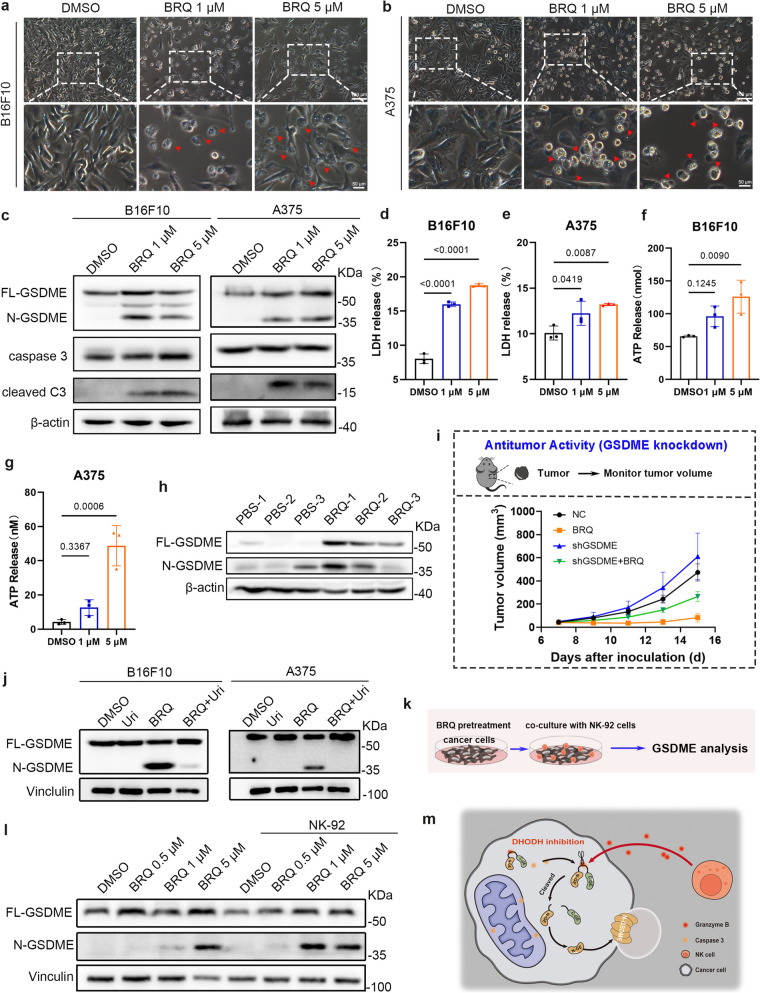
BRQ induces pyroptosis in melanoma cells and synergizes with NK cells. **a-b** Representative images of morphological alterations after BRQ treatment in B16F10 and A375. Arrow indicated cell swelling and rupture. **c** Western blot analysis of GSDME and caspase 3 in B16F10 and A375. **d-e** LDH released in culture supernatants after BRQ treatment in B16F10 and A375 cells (*n* = 3). **f-g** ATP released in culture supernatants after BRQ treatment in B16F10 and A375 cells (*n* = 3). **h** Western blot analysis of GSDME in tumor tissues (*n* = 3). **i** Tumor growth curves in different experimental groups (*n* = 6). **j** Western blot analysis of GSDME in untreated, uridine treated, BRQ treated or BRQ and uridine treated tumor cells. **k** In vitro experiments for NK cells enhancing tumor cells pyroptosis: A375 cells were treated with BRQ for 24 h and then co-cultured with NK-92 cells for 2 h to analysis the expression of GSDME in A375 cells. **l** Western blot analysis of GSDME in indicated groups as shown in **k**.** m** Schematic representation of NK cells aggravation of cancer cells pyroptosis

Notably, the formation of pores on the plasma membrane was observed following stimulation with BRQ in both B16F10 and A375 (Fig. 5a-b), consistent with the hallmark features of pyroptosis. Pyroptosis, a significant innate immune response [31], is closely related to NK cells. Granzyme A from NK cells and cytotoxic T lymphocytes [32], as well as killer-cells granzyme B, have been reported to initiate pyroptosis in target cells by cleaving GSDMB and GSDME, respectively. Importantly, the absence of GSDME in tumors results in reduced NK cells presence in the tumor microenvironment [33]. Thus, we explored the precise mechanism by which BRQ induces pyroptosis and the role of NK cells in activating tumor cells pyroptosis.

The DEGs were enriched in the NOD-like receptor signaling pathway (Fig. 3a). Among these, NLRP3 holds particular significance as a crucial member of the NOD-like receptor family. The activation of inflammasomes orchestrated by NLRP3 can lead to pyroptosis, a process reliant on GSDMD [34]. To investigate whether BRQ induces pyroptosis through GSDMD, the protein expression of GSDMD was assessed. However, it was observed that BRQ did not induce the production of N-GSDMD in either A375 or B16F10 cell lines (Fig. S4a-b). Existing studies have shown that GSDMA can act as an effector protein for pyroptosis [35]; and that the GZMA-GSDMB axis constitutes a novel pyroptotic pathway through which NK cells can kill target cells directly [32]. Furthermore, activated caspase 8 can cleave GSDMC to produce an active N-terminal fragment that targets the cell membrane, forming pores and inducing pyroptosis [36]. However, the results revealed that BRQ treatment did not activate GSDMA, GSDMB, or GSDMC in tumor cells. (Fig. S4a-b), indicating that the anti-tumor effect of BRQ may not depend on these known pyroptotic pathways. Nevertheless, pyroptosis can also be elicited through caspase 3 cleavage of GSDME [37, 38]. Interestingly, both cleaved caspase 3 and N-GSDME demonstrated a significant increase in both A375 and B16F10 cells following BRQ incubation, while the full-length GSDEM (FL-GSDME) and caspase 3 remained unchanged (Fig. 5c). These results indicate that BRQ activates caspase 3 and triggers pyroptosis by cleaving GSDME.

Another characteristic of pyroptosis is the release of cellular contents. The results showed that lactate dehydrogenase (LDH) release increased in a dose-dependent manner after BRQ treatment, demonstrating plasma membrane rupture and leakage (Fig. 5d-e). Additionally, adenosine triphosphate (ATP), a type of damage-associated molecular pattern (DAMP), which can be released from pyroptotic cells, was significantly increased in the cells supernatant (Fig. 5f-g). Furthermore, flow cytometry analysis of propidium iodide (PI) and annexin Ⅴ staining revealed that BRQ markedly induced cell death (Fig. S4c-d). This mechanism extended beyond melanoma, as MC38 colorectal cancer cells similarly exhibited GSDME-dependent pyroptosis (Fig. S4e), indicating that GSDME may be a key effector molecule common to BRQ-induced pyroptosis in tumor cells.

To further validate this conclusion in vivo, we examined tumors from three mice per group to detect GSDME protein expression. Remarkably, N-GSDME were noticeably upregulated (Fig. 5h), which concurred with the findings from immunohistochemistry (Fig. S4f). Additionally, in response to BRQ treatment, the expression of high-mobility group box 1 (HMGB1) in tumors increased (Fig. S4g), which contribute to the activation of DC cells (Fig. 1h-j). Crucially, the ablation of GSDME significantly attenuated the anti-tumor efficacy of BRQ. (Fig. 5i, S4h-i), establishing the functional importance of this pathway. Moreover, exogenous supplementation with uridine inhibited the production of N-GSDME, indicated that BRQ induced pyroptosis is related to pyrimidine metabolism (Fig. 5j).

Notably, we identified a positive feedback loop between NK cells and pyroptosis. While NK-92 cells alone induced modest GSDME cleavage (Fig. S4k), their combination with BRQ pretreatment synergistically enhanced pyroptosis (Fig. 5k-l). This reciprocal interaction, wherein BRQ-induced pyroptosis promotes NK cell infiltration which further amplifies pyroptosis, represents a crucial mechanism for BRQ's immunotherapeutic effects (Fig. 5m).

### Mitochondrial oxidative stress as the origin of BRQ induced anti-tumor immunity

To explore the origin of BRQ induced anti-tumor immunity, we focus on the unique properties of DHODH. DHODH, situated on the external surface of the mitochondrial inner membrane, establishes a connection with the respiratory chain through the coenzyme Q pool [5]. The deficiency in DHODH activity impedes the proper functioning of the respiratory chain, consequently leading to the initiation of oxidative damage [39]. Given that mitochondria serve as pivotal center for immune responses [40], we postulate that BRQ-induced anti-tumor immunity originates from mitochondrial oxidative stress.

**Fig. 6:**
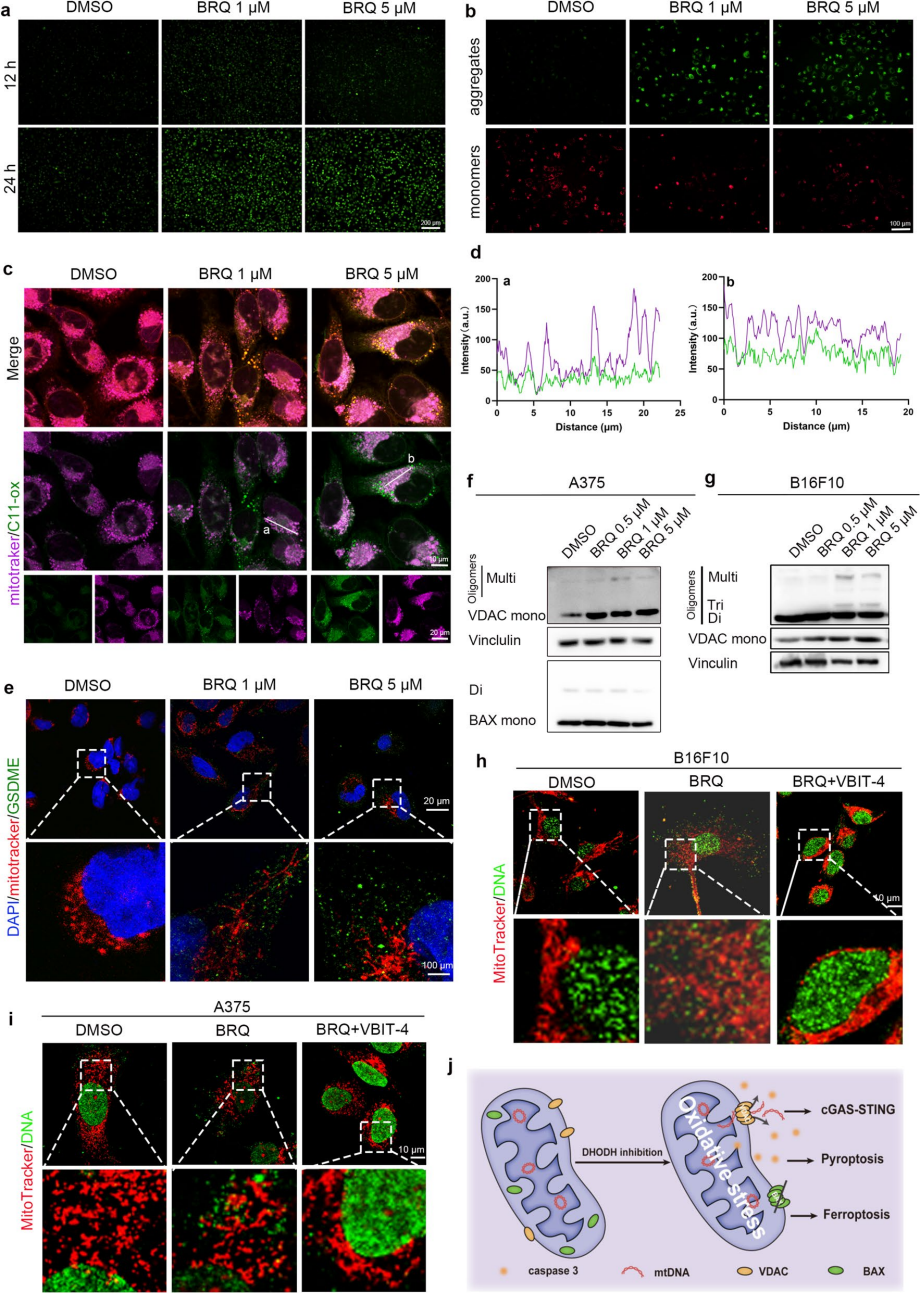
BRQ induces mitochondrial oxidative stress and mtDNA released via VDAC. **a** ROS was detected by DCFH-DA in A375 cells. **b** JC-1 analysis in A375 cells. **c** Representative images of A375 co-stained BODIPY C11 (Oxidized BODIPY-green/non oxidized BODIPY-red) and mitochondrial (purple). **d** Colocalization analysis of mitochondrial and oxidized BODIPY in **c**. **e** Representative images of A375 co-stained mitochondrial(red) and GSDME (green). DAPI was used to stain nuclei. **f** Western blot analysis of VDAC and BAX in A375 cells. **g** Western blot analysis of VDAC in A375 cells. **h-i** CLSM images of mtDNA released from mitochondrial in (h) B16F10 and (i) A375 (Red: mitochondrial; green: DNA). **j** Schematic showing the release of mtDNA from mitochondria

To assess mitochondrial impairment, we initially detected reactive oxygen species (ROS) production subsequent to BRQ stimulation. Notably, BRQ was found to increase ROS levels in a concentration- and time-dependent manner (Fig. 6a and Fig. S5a), while also reducing mitochondrial membrane potential (Fig. 6b and Fig. S5b).

Considering the susceptibility of mitochondrial membranes to oxidative injury and the protective role of DHODH against lipid peroxidation, particularly in glutathione peroxidase 4 (GPX4) low expression cells [41], we subsequently measured mitochondrial lipid peroxidation. In comparison to the 786-O cell line (GPX4^high^) [41], BRQ significantly enhanced mitochondrial lipid peroxidation in GPX4^low^ melanoma cells (A375 and B16F10) (Fig. 6c–d and S5c–e). As BRQ treatment has been shown to significantly increase intracellular ROS and lipid peroxide levels, while decreasing mitochondrial membrane potential, we hypothesised that ferroptosis might be involved in the cell death process induced by BRQ. Ferroptosis is an iron-dependent form of regulated cell death characterised by lipid peroxidation [41]. Subsequent experiments demonstrated that the ferroptosis inhibitor Ferrostatin-1 (Fer-1) attenuated BRQ-induced cell death (Fig. S5f-h), confirming concurrent ferroptosis activation. To determine whether the tumor-killing effect of BRQ is mediated by the inhibition of DHODH, we treated B16F10 cells with BRQ after knocking down DHODH expression. The results showed that DHODH knockdown abolished these effects (Fig. S5i-l), verifying target specificity. Collectively, the comprehensive findings underscore the pronounced induction of oxidative stress within the mitochondrial by BRQ treatment.

Given the release of mtDNA as observed in Fig. 4a, we delved into the underlying mechanism governing mtDNA release from damaged mitochondria. Mitochondrial outer membrane permeabilization (MOMP) emerged as a key contributor to mtDNA release. Following MOMP, the gradual expansion of outer membrane pores triggers extrusion and rupture of the inner mitochondrial membrane (IMM) [42]. Although mitochondrial membrane permeabilization can be facilitated by GSDME-N [43], it is noteworthy that despite BRQ significantly elevating GSDME expression, no discernible co-localization between mitochondria and GSDME was detected (Fig. 6e). These observations negate the involvement of GSDME-N in mtDNA release.

Moreover, BAX/BAK oligomerization, known to form pores in the IMM that facilitates mtDNA release [44], was not observed in A375 cells after BRQ treatment (Fig. 6f). Voltage-dependent anion channel (VDAC) oligomers have been recognized as inducers of mtDNA release [45]. Indeed, VDAC oligomerization facilitated mtDNA efflux, as demonstrated by oligomer detection (Fig. 6f-g) and VBIT4-mediated inhibition of mtDNA release (Fig. 6h-i, S5m) [46]. In summary, the release of mtDNA from compromised mitochondria instigates the activation of the cGAS-STING signaling pathway. Concurrently, mitochondrial oxidative stress plays a pivotal role in expediting pyroptosis [47]. Cumulatively, these findings underscore the critical role of damaged mitochondria as the underlying source driving BRQ-induced anti-tumor immunity (Fig. 6j).

### EA6, a more effective DHODH inhibitor

**Fig. 7:**
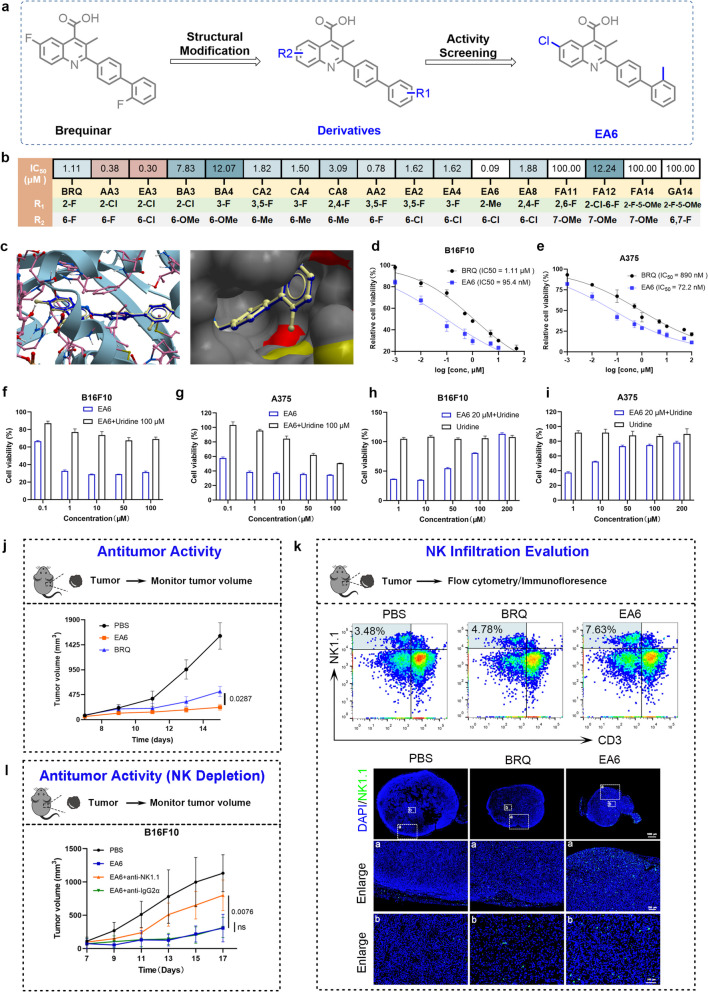
EA6, a more effective DHODH inhibitor. **a** Structural transformation from BRQ to EA6. **b** IC_50_ values of BRQ and BRQ derivatives drugs to B16F10 cell lines at 72 h. **c** Comparison of the binding sites of BRQ (blue) and EA6 (gray) with DHODH (PDB ID:1D3G). **d-e** Cell viability in B16F10 or A375 treated with BRQ or EA6 (*n* = 3). **f-g** Cell viability in B16F10 or A375 treated with EA6 or EA6 + uridine (*n* = 3). **h-i** Cell viability in B16F10 or A375 treated with uridine or uridine + EA6 (n = 3). **j** B16F10 tumors growth in mice treated with vehicle, BRQ (30 mg/kg, i.p.) or EA6 (30 mg/kg, i.p.) once ever two days (*n* = 5). **k** Representative flow cytometry images and immunofluorescence images of NK cells in tumors from PBS, BRQ (30 mg/kg, i.p.) or EA6 (30 mg/kg, i.p.) treated mice. Mice were treated every two days, and after three times the tumors was collected for testing. **l** B16F10 tumor growth in mice treated with PBS, EA6 (30 mg/kg, i.p.), the combination of EA6 (30 mg/kg, i.p.) and anti-IgG2α (300 ug, i.p.) or the combination of EA6 (30 mg/kg, i.p.) and anti-anti-NK1.1(300 ug, i.p.) (*n* = 3)

In the quest to enhance the anti-tumor efficacy of BRQ, a series of derivatives based on BRQ were designed and synthesized (Fig. 7a). Initially, by introducing electron-withdrawing groups such as F and Cl to the 6th position of the quinoline ring, compounds AA3, EA3 were obtained, and by introducing electron-donating groups such as -CH_3_ and -OCH_3_ to the quinoline ring, compounds BA3, BA4, CA2, CA4, and CA8 were obtained. Activity screening revealed that, compared to BRQ, replacing electron-withdrawing groups on the 6th position of the quinoline ring with electron-donating groups resulted in better anti-tumor activity.

Subsequently, on the basis of introducing F and Cl at the 6th position of the quinoline ring, we further explored the effects of substituents at different positions on the biphenyl ring on its activity, then designed and synthesized compounds AA2, EA2, EA4, EA6, and EA8. The results showed that the compound with -CH_3_ substitution on the biphenyl ring exhibited significantly better anti-tumor activity than the F and Cl substitutions.

In addition, to further investigate whether anti-tumor activity could be effectively enhanced by introducing -OCH_3_ at the 7th position of the quinoline ring and introducing F at both the 6th and 7th positions simultaneously, compounds FA11, FA12, FA14, and GA14 were synthesized. Unfortunately, these structural modifications still exhibited poor anti-tumor activity. Ultimately, EA6 was selected as the compound with the highest activity (Fig. 7b and Table S1).

In order to elucidate the molecular mechanism behind the enhanced activity of EA6, we first analysed the binding modes of EA6 and BRQ to the DHODH protein (PDB: 1D3G) using molecular docking. The docking results showed that the methyl substituent on the biphenyl ring of EA6 could bind more deeply to the hydrophobic pocket of DHODH than BRQ could (Fig. 7c), providing a structural basis for EA6's enhanced inhibitory activity. Circular dichroism (CD) spectroscopy confirmed that both compounds induce conformational changes in DHODH, with EA6 producing more pronounced structural alterations (Fig. S6a). EA6 exhibited approximately tenfold greater cytotoxicity than BRQ in melanoma cells (Fig. 7d-e) and induced significant cell death at 500 nM (Fig. S6b). The specificity of EA6 for DHODH was validated through uridine rescue experiments, which completely reversed its anti-proliferative effects (Fig. 7f-i).

Subsequently, the in vivo anti-tumor effect of EA6 was evaluated. The results showed that, compared with BRQ, EA6 had a more significant inhibitory effect on tumor growth and no obvious systemic toxicity (Fig. 7j, S6c). Additionally, the expression levels of GSDME and p-STING were upregulated by EA6 (Fig. S6d), and examination of the major organs after treatment revealed no severe structural or pathological changes (Fig. S6e). Taken together, EA6 induces melanoma cells pyroptosis and activates the cGAS-STING signaling pathway in vivo, which demonstrated superior anti-tumor activity.

Importantly, EA6 demonstrated enhanced efficacy in promoting NK cell infiltration into tumors, flow cytometry analysis showed that EA6 treatment increased the proportion of NK cells from 3.48% to 7.63% of CD45^+^ lymphocytes (Fig. 7k and Fig. S6f). As depicted in Fig. 7k. in the PBS group, NK cells were mainly aggregated at the tumors periphery and were less abundant. Conversely, following treatment with BRQ/EA6, a significant infiltration of NK cells into the tumor core was evident, indicating indicating that EA6 had a higher capacity to promote NK cells infiltration within the tumor microenvironment. In addition, NK cell depletion experiments further established that EA6's anti-tumor efficacy depends on NK cell activity (Fig. 7l, S6g-h).

In summary, EA6 emerged as a more potent DHODH inhibitor, demonstrating strong tumor regression in melanoma and acting as an NK cells-dependent therapeutic agent.

## Discussion

In recent years, our research group has primarily focused on tumor metabolism and cancer immunotherapy [48, 49]. Restraining nucleotide metabolism is a therapeutic target not only for chemotherapy, but also for improving the efficacy of cancer immunotherapy. Disrupting nucleotide metabolism increases genomic instability by destroying purine and pyrimidine pools, which ultimately promotes immunogenicity [3]. DHODH, a key rate-limiting enzyme in de novo pyrimidine synthesis, plays an important role in tumor immunity [10, 12]. However, the effect of DHODH inhibition on NK cells remains unexplored. Since malignant melanomas frequently escape immunosurveillance through MHC-I downregulation, rendering them susceptible to NK cell-mediated killing, we investigated whether NK cells contribute to DHODH inhibition-induced anti-tumor immunity in melanoma.

Our study revealed distinct responses between CD8^+^ T cells and NK cells to DHODH inhibition. While BRQ treatment did not significantly increase CD8^+^ T cell infiltration, it induced remarkable NK cell accumulation in tumors. NK cell depletion substantially diminished the anti-tumor effect of DHODH inhibition, highlighting their essential role in this process.

The pivotal role of mitochondria in cellular energy production is well documented, but their emerging importance as centres of immune responses has opened up new areas of research [22]. DHODH is located on the outer surface of the inner mitochondrial membrane and is connected to the respiratory chain, providing a compelling link between mitochondrial function and immune activation. It has been revealed that high concentrations of BRQ induce mitochondria-associated ferroptosis in cancer cells [41, 50], further substantiating the link between DHODH and mitochondrial-induced immune responses.

Our investigation demonstrates that DHODH inhibition triggers mitochondrial oxidative stress, leading to VDAC oligomerization and subsequent mtDNA release. Cytosolic mtDNA then activates cGAS-STING signaling—a critical driver of anti-tumor immunity. Concurrently, mitochondrial damage promotes caspase-3-mediated GSDME cleavage, inducing pyroptosis and DAMP release that further amplify immune responses.

The production of IFN-I is a hallmark of STING pathway activation. IFN-I plays a crucial role in NK cell homeostasis, activation and antitumour functionality [27]. We found that BRQ promoted NK cell activity through tumor-derived IFN-β, with significant IFN-β upregulation observed in the tumor microenvironment. These observations further elucidate the role of DHODH inhibition in facilitating NK cell infiltration by upregulating IFN-I.

Notably, we identified a positive feedback loop between NK cells and pyroptosis. NK cell-derived granzyme B cleaves GSDME at the same site as caspase-3 [33], and co-culture with NK cells enhanced GSDME cleavage in tumor cells. This reciprocal enhancement between NK cells and pyroptosis significantly contributes to the anti-tumor immune response.

Finally, we developed EA6, an optimized DHODH inhibitor with superior activity. EA6 demonstrated significantly enhanced potency in inducing NK cell infiltration and suppressing tumor growth compared to BRQ.

**Fig. 8:**
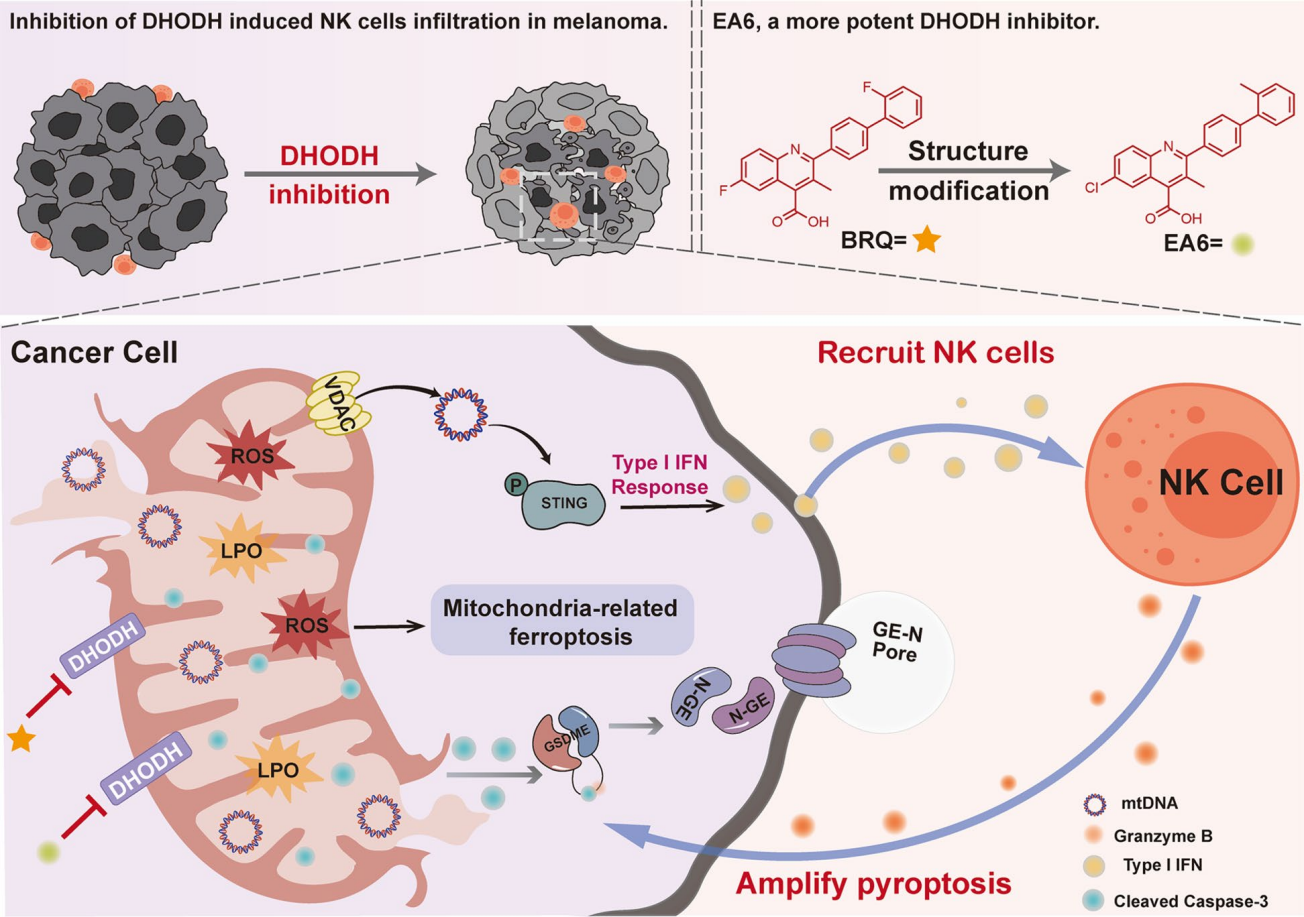
Anti-tumor mechanisms of DHODH inhibition. DHODH inhibition induces mitochondrial oxidative stress and VDAC oligomerization, leading to mitochondrial DNA release and subsequent activation of the cGAS-STING pathway. This promotes NK cell infiltration into tumors. Infiltrating NK cells enhance GSDME-mediated pyroptosis in cancer cells, while mitochondrial oxidative stress concurrently triggers ferroptosis. These processes establish a positive feedback loop that amplifies the anti-tumor immune response

In conclusion, our study establishes that DHODH inhibition activates pyroptosis and cGAS-STING signaling to promote NK cell-mediated anti-tumor immunity (Fig. 8). These findings provide new avenues for enhancing nucleotide metabolism-targeted cancer therapies and support the potential of combining DHODH inhibitors with immunotherapy approaches.

While our findings demonstrate the promise of DHODH inhibition in NK cell-mediated immunity, several questions remain. Future studies should focus on elucidating the precise regulatory mechanisms governing NK cell activation and their interactions with other immune components. Additionally, developing combination strategies that integrate DHODH inhibitors with existing immunotherapies could enhance anticancer efficacy while minimizing adverse effects, potentially leading to more targeted and personalized cancer treatments.

## Materials and methods

### Cell culture

The B16F10 (murine melanoma cells), A375 (human melanoma cells), 786-O (human renal carcinoma cells), PUMC-HUVEC-T1 (human endothelial cells) and NK-92 (human natural killer cells) were procured from Procell Life Science & Technology (Wuhan, China). B16F10, 786-O, PUMC-HUVEC-T1 and A375 cells were cultured in DMEM/RPMI-1640 medium supplemented with 10% fetal bovine serum (Excell Bio, Shanghai, FSP500) and 1% penicillin/streptomycin (MacGene, Beijing, CC004). NK-92 cells were cultured with specific medium (CM-0530) procured from Procell Life Science & Technology. Incubation of all cell lines was carried out at 37 °C under a 5% CO_2_ atmosphere. In this study, the cell lines involved were confirmed to be correct and free of contamination after short tandem repeat (STR) analysis and quality inspection.

### Mice and anti-tumor therapy

Female C57BL/6 mice aged 6–8 weeks were obtained from SPF (Beijing) Biotechnology Co., Ltd. B16F10 cells were harvested and suspended in serum-free RPMI-1640 medium. Subsequently, 5 × 10^6^ cells were subcutaneously injected into each mouse. When the tumor size reached approximately 50 mm^3^, the mice were treated with PBS (intraperitoneal injection, once every two days), BRQ (intraperitoneal injection, 30 mg/kg, once every two days), or EA6 (intraperitoneal injection, 30 mg/kg, once every two days).

For NK cells depletion, 250 μg of anti-NK1.1 or IgG2α antibodies were diluted in dilution buffer (InVivoPure pH 7.0 Dilution Buffer) and intraperitoneally injected into the mice on days 6, 8, 10, and 12. Spleens were collected 24 h after the first injection of anti-NK1.1 antibody for flow cytometry analysis to verify the depletion of NK cells.

The tumor volumes and body weights of the mice were measured every 2 days. The tumor volume (V) was calculated using the following equation, where 'a' represents the longest dimension of the tumor and 'b' represents the shortest dimension of the tumor: V = a*b^2^/2.

### Western blot

After the indicated treatment duration, cells were lysed and protein concentrations were determined using the BCA Protein Assay Kit (Beyotime). Proteins (20 μg per sample) were separated by SDS-PAGE and transferred to PVDF membranes. After blocking with 5% non-fat milk, membranes were incubated overnight with primary antibodies, followed by incubation with corresponding secondary antibodies. Protein bands were visualized using an ECL substrate and a chemiluminescence imaging system. Detailed information of antibodies as shown in Table S1 in supporting information.

### Enzyme-linked immunosorbent assay

After a 36 h treatment with BRQ, the culture medium was collected from the cells and centrifuged for subsequent analysis. In the animal study, tumors were collected at the indicated time points and lysed with ELISA extraction buffer on ice for 2 h. The supernatant was then centrifuged for further analysis. The concentrations of IL-10, IL-12, IL-1B, TGF-β, and IFN-γ were determined using an ELISA kit (Fanke, Shanghai) according to the manufacturer's instructions.

### Cell transfection

Small interfering RNA (siRNA) targeting DHODH (siDHODH) and negative control siRNA (siNC) were obtained from Tsingke Biotechnology Co., Ltd. (Beijing, China). Transient transfection was performed using Lipofectamine 2000 (Invitrogen, 11,668–019) with 100 nM siRNA pools targeting human or mouse genes. For stable knockdown, short hairpin RNA (shRNA) targeting STING or GSDME and a control shRNA (shNC) were cloned into lentiviral vectors (Tsingke). B16F10 cells were transduced with lentivirus and selected with puromycin to establish stable knockdown cell lines. All siRNA and shRNA sequences are listed in Tables S3–S5.

### NK cell-induced cytotoxicity to BRQ-treated A375 cells

A375 cells were pretreated with BRQ for 30 h and then replacement of fresh medium to continuation incubation for 6 h. Subsequently, NK-92 cells were added to A375 cells in a ratio of 1:5 and cocultured for 12 h. Finally, cell viability of A375 were evaluated by MTT assay.

To characterize NK-92 cells recognition of A375 cells, briefly, A375 cells were labeled with Hochest 33,342 and NK92 cells were labeled with CSFE. After co-culture with the transwell system, the contact between NK-92 cells and A375 cells were evaluated by confocal laser scanning microscopy (CLSM).

### Immunofluorescence

Cells grown on coverslips were fixed with 4% paraformaldehyde and permeabilized with 0.2% Triton X-100 when required. After blocking with 3% BSA, cells were incubated with primary antibodies at 4 °C overnight, followed by fluorescence-conjugated secondary antibodies. Nuclei were counterstained with DAPI, and images were acquired using CLSM.

### Mitochondrial function analysis

The mitochondrial membrane potential was evaluated using the Enhanced Mitochondrial Membrane Potential Assay Kit with JC-1 (C2003S, Beyotime). The status of the mitochondrial permeability transition pore (mPTP) was assessed using the Mitochondrial Permeability Transition Pore Assay Kit (C2009S, Beyotime). A375 and B16F10 cells were treated with BRQ for 24 h, stained according to manufacturer protocols, and visualized by fluorescence microscopy (OLYMPUS IX73) or CLSM.

### LDH and ATP release assay

A375 and B16F10 cells were treated with the specified concentrations of BRQ for 48 h. After centrifugation at 3000 rpm for 5 min, the supernatant was collected for the detection of LDH release and ATP levels. The LDH Release Assay Kit and Enhanced ATP Assay Kit were used, following the provided instructions, to perform these measurements.

### Flow cytometry analysis

After three rounds of treatment, the mice were euthanized to examine their immune phenotype. The analysis focused on mature DC cells (CD11c^+^CD80^+^CD86^+^), T cells (CD45^+^CD3^+^CD4^+^/CD8^+^), and NK cells (CD45^+^CD3^−^NK1.1^+^). Tumors, spleens, and lymph nodes were dissected, followed by digestion or homogenization to obtain single cells. These cells were then stained with specific antibodies and analyzed using flow cytometry. FlowJo_V10 software was utilized for data analysis and interpretation.

### Inhibitors treatment

VDAC inhibitor treatment: Pre-treat the cells with 10 μM of the VDAC oligomerisation inhibitor VBIT-4 (S3544, SelleckChem) for 30 min and then proceed with the BRQ immunofluorescence staining steps. Ferroptosis inhibitor treatment: Pre-treat the cells with 20 μM Fer-1 (S7243, SelleckChem) for 30 min and then add different concentrations of BRQ for 48 h.

### RNA-seq

Total RNA was extracted from B16F10 cells treated with DMSO or BRQ for 24 h using Trizol (Thermofisher, 15,596,018). RNA libraries were constructed and sequenced on the Illumina Novaseq™ 6000 platform (LC Bio Technology, Hangzhou). Differential gene expression analysis was performed via OmicStudio (https://www.omicstudio.cn/tool), with significance thresholds set at |log₂FC|≥ 1 and *q*-value < 0.05.

### Tissue microarrays chips assay

Melanoma tissue microarrays were obtained from ZhuoLi Biotech Co., Ltd. (Shanghai, China), comprising 5 normal skin tissues and 44 melanoma tumor tissues, with duplicate cores per case for experimental reproducibility. The TMA was accompanied by detailed clinicopathological data for all patients, enabling integrated analysis of molecular findings with clinical features.

### Statistical analysis

The data analysis was performed using GraphPad Prism 9.5 software. The results are presented as mean ± SEM (standard error of the mean) or mean ± SD (standard deviation), depending on the specific analysis. Each experiment was repeated independently at least three times to ensure the reliability of the findings. Statistical significance was determined using the student's t-test for comparisons between two groups, and One-way ANOVA followed by post hoc tests for comparisons among multiple groups. *P* value of less than 0.05 was considered statistically significant.

## Supplementary Information

Supplementary Material 1.

## Data Availability

The RNA-sequencing data generated by this study have been deposited to GEO database under accession number: GSE245151. Other data are available in the main text or the Supplementary Information.
