# Supplementary material for: Dihydroorotate dehydrogenase inhibition activates STING pathway and pyroptosis to enhance NK cell-dependent tumor immunotherapy
Source: Mol Biomed. 2025 Oct 27;6:87. doi: 10.1186/s43556-025-00339-7 (PMC12559559; doi:10.1186/s43556-025-00339-7)

Supplementary Information

Title:

Dihydroorotate dehydrogenase inhibition activates STING pathway and pyroptosis to enhance NK cell-dependent tumor immunotherapy

Authors:

Yongrui Hai[1, 2, †], Ruizhuo Lin[1, 2, †], Weike Liao[3, †], Shuo Fu[4], Renming Fan[1, 2], Guiquan Ding[4], Junyan Zhuang[1, 2], Bingjie Zhang[1, 2], Yi Liu[5*], Junke Song[4*], Gaofei Wei[1, 2*]

Affiliation and address:

[1] Laboratory of Cellular Metabolism and Precision Therapeutics, Institute of Medical Research, Northwestern Polytechnical University, Xi'an 710072, China.

[2] Research & Development Institute of Northwestern Polytechnical University in Shenzhen, Shenzhen 518057, China.

[3] Guizhou Provincial Engineering Technology Research Center for Chemical Drug R&D, Guizhou Medical University, Guiyang 550004, China.

[4] Beijing Key Laboratory of Drug Target Identification and Drug Screening, Institute of Materia Medica, Chinese Academy of Medical Sciences & Peking Union Medical College, Beijing 100050, China.

[5] Department of Medical Oncology, Shaanxi Provincial People's Hospital, Xi'an 710068, China.

[†] These authors contributed equally to this work.

***Corresponding authors:** liuyi@spph-sx.ac.cn(Yi Liu); smilejunke@imm.ac.cn (Junke Song); weigf0605@163.com (Gaofei Wei)

Supplemental Figures and Figure legends

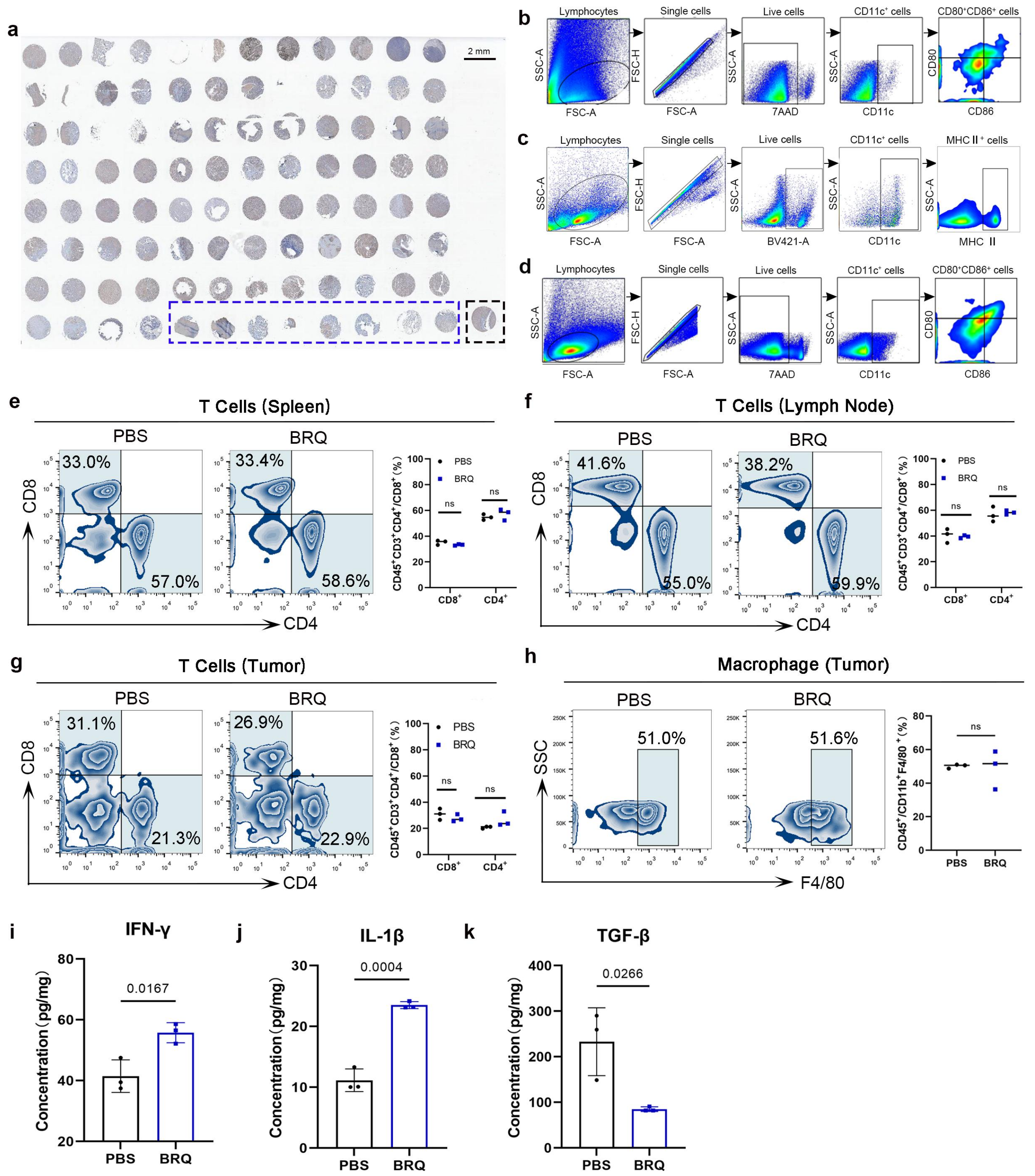

Fig. S1 BRQ restrains melanoma growth. **a** Full view of melanoma tissue microarray. (Black bordered rectangle: localization point; Blue bordered rectangle: normal tissues; others: melanoma tissues). **b** Flow gating strategy of DCs (CD11c$^+$CD80$^+$CD86$^+$) in tumors. **c** Flow gating strategy of DCs (MHC II$^+$) in tumors. **d** Flow gating strategy of DCs (CD11c$^+$CD80$^+$CD86$^+$) in lymph nodes.

e-g Representative flow cytometry images and quantitative graph of T cells in (e) spleen, (f) lymph nodes and (g) tumor (n = 3). **h** Representative flow cytometry images and quantitative graph of macrophage in tumor (n = 3). **i-k** The concentration of IFN-γ, IL-1β and TGF-β in tumors were analyzed by ELISA (n = 3).

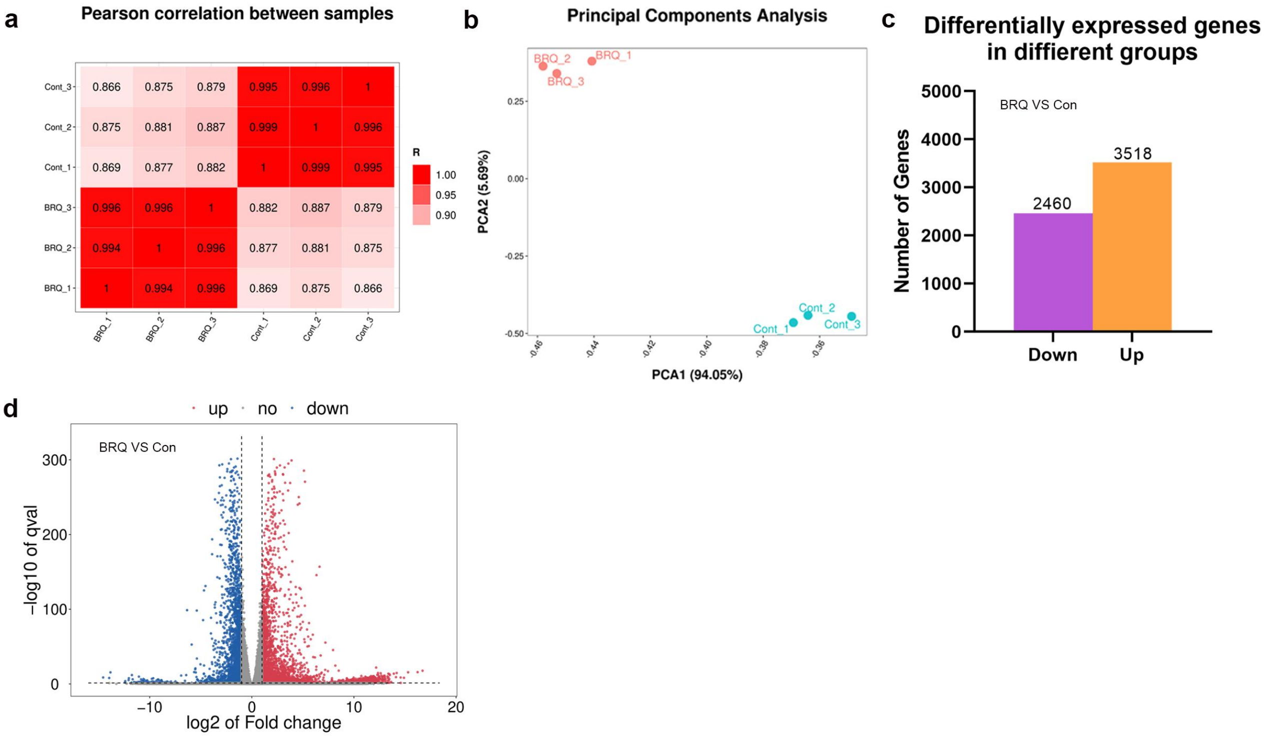

Fig. S2 RNA-Seq analysis of B16F10 cells treated with BRQ. **a** Pearson correlation between the BRQ group and the control group. **b** PCA analysis between the BRQ and the control group. **c** The number of DEGs after BRQ treatment. **d** The volcano plot of DEGs in BRQ and the control group.

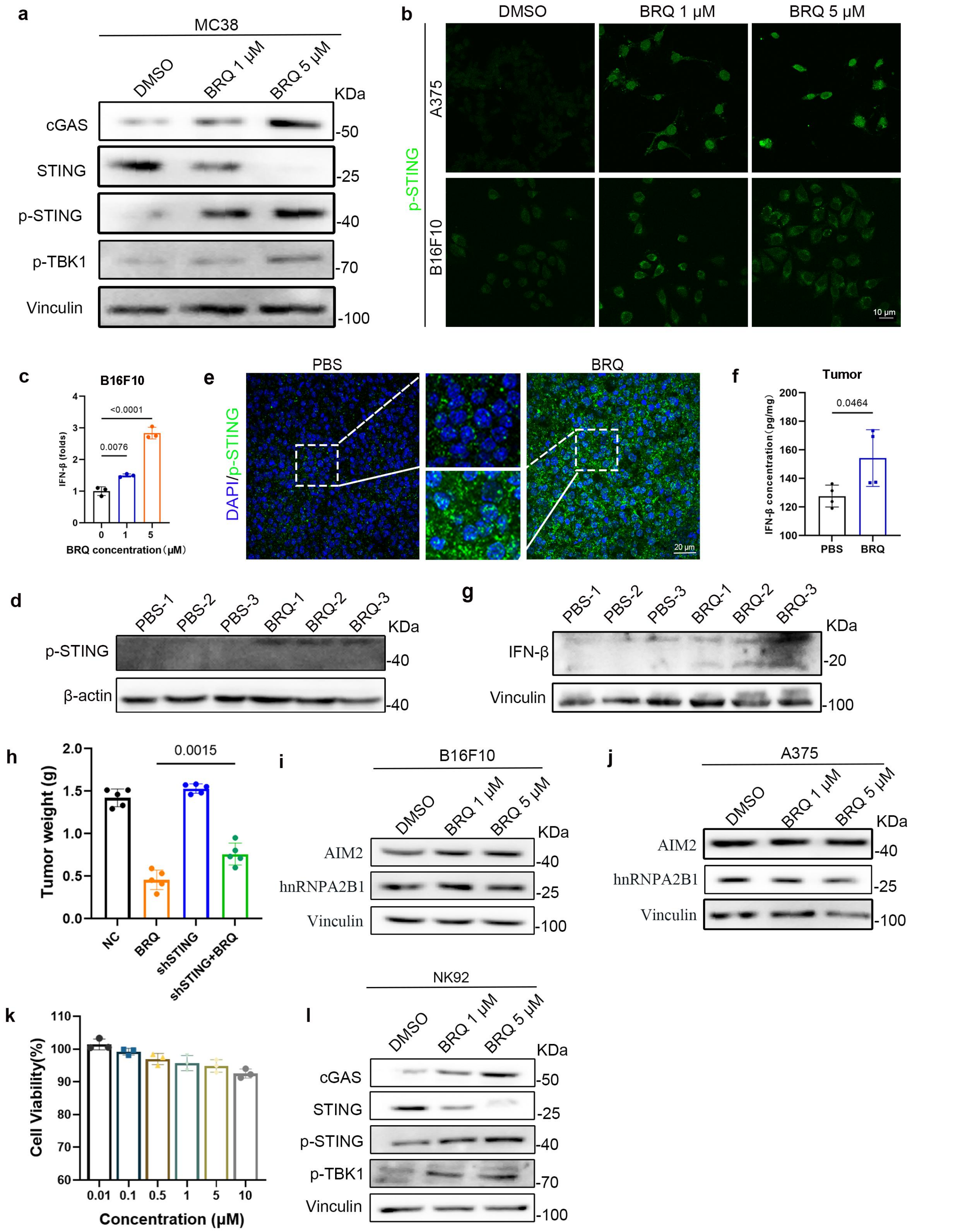

Fig. S3 BRQ activation of the cGAS-STING pathway enhances the antitumor immunity of NK cells.

a Western blot analysis of protein involved in cGAS-STING pathway. **b** CLSM images of p-STING

expression in BRQ treatment and control group. **c** ELISA analysis of INF-β in culture medium of untreated, BRQ treated B16F10 cells (n = 3). **d** Western blot analysis of p-STING in tumor tissue (n = 3). **e** Immunostaining of p-STING in tumor tissue (Blue: DAPI; green: p-STING). **f** ELISA analysis of INF-β in tumors of untreated or BRQ treated mice (n = 3). **g** Western blot analysis of IFN-β in tumor tissue (n = 3). **h** The average tumor weight at the end of the experiment (n = 5). **i-j** Western blot analysis of AIM2 and hnRNPA2B1 in (i) B16F10 and (j) A375 cells. **k** Cell viability of NK92 cells after treatment with different concentrations of BRQ (n = 3). **l** Western blot analysis of protein involved in cGAS-STING pathway.

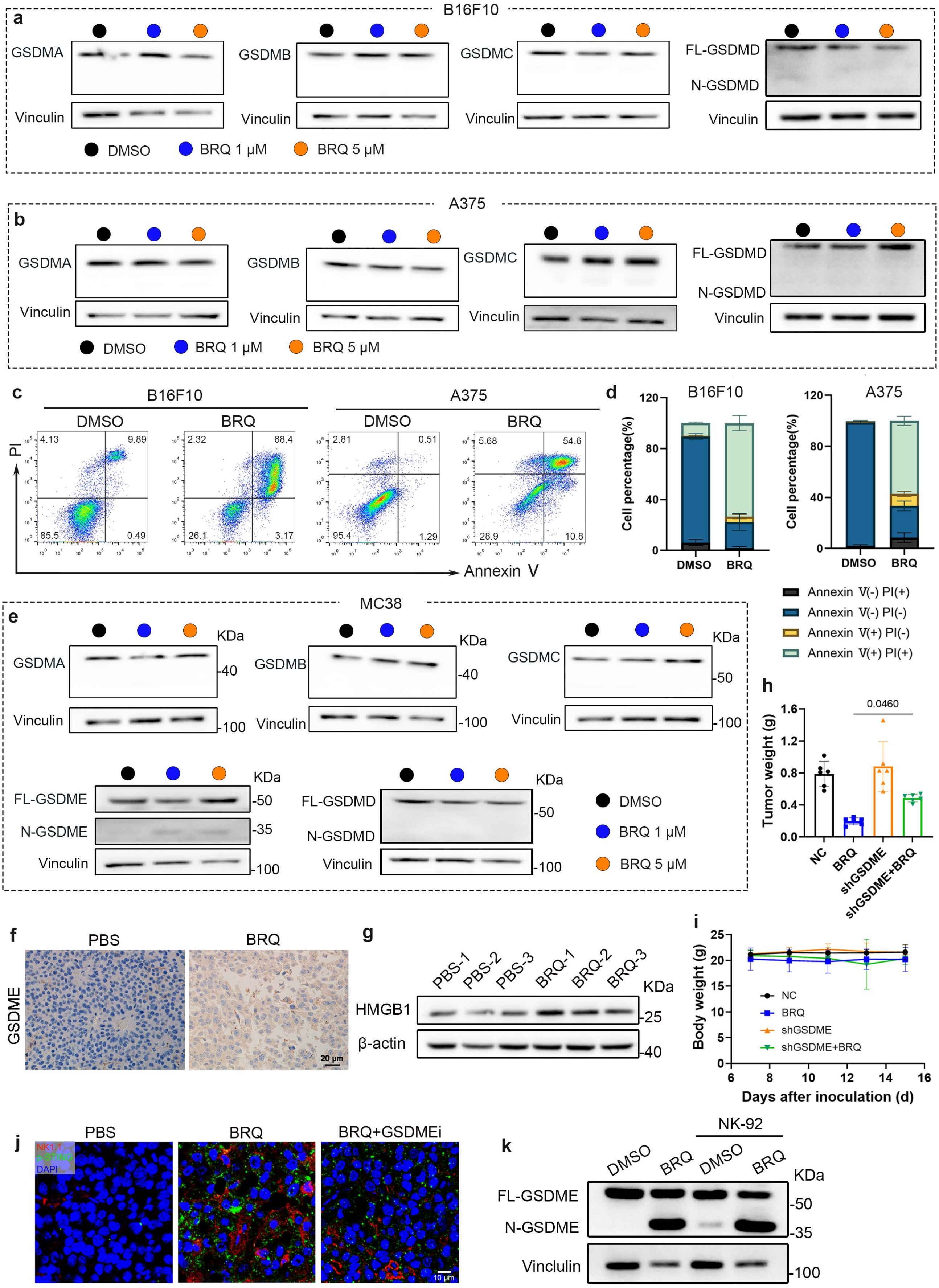

B16F10
a
GSDMA
GSDMB
GSDMC
FL-GSDMD
N-GSDMD
Vinculin
DMSO
BRQ 1 μM
BRQ 5 μM
A375
b
c
B16F10
A375
DMSO
BRQ
PI
Annexin V
d
Cell percentage(%)
Annexin V(-) PI(+)
Annexin V(-) PI(-)
Annexin V(+) PI(-)
Annexin V(+) PI(+)
MC38
e
KDa
FL-GSDME
N-GSDME
h
Tumor weight (g)
0.0460
NC
BRQ
shGSDME
shGSDME+BRQ
f
PBS
GSDME
20 μm
g
PBS-1
PBS-2
PBS-3
BRQ-1
BRQ-2
BRQ-3
HMGB1
β-actin
i
Body weight (g)
Days after inoculation (d)
j
BRQ+GSDMEi
DAPI
10 μm
k
NK-92
Vinclulin

Fig. S4 BRQ induces pyroptosis in melanoma cells and synergizes with NK cells. **a-b** Western blot analysis of GSDMA, GSDMB, GSDMC and GSDMD in (a) B16F10 and (b) A375 cells. **c** Flow analysis of cells death after BRQ treatment. **d** Quantification of single PI positive, single FITC-Annexin V positive, and FITC-Annexin V/PI double positive or negative cells. **e** Western blot analysis of GSDMA, GSDMB, GSDMC, GSDMD and GSDME in MC38 cells. **f** Immunohistochemistry analysis of GSDME expression in tumor tissue in BRQ treatment and control group. **g** Western blot analysis of HMGB1 in tumor tissue (n = 3). **h** The average tumor weight at the end of the experiment (n = 6). **i** Body weight of mice (n = 6). **j** Representative immunofluorescence images of NK cells and p-STING in tumor tissues. **k** Western blot analysis of GSDME in indicated groups.

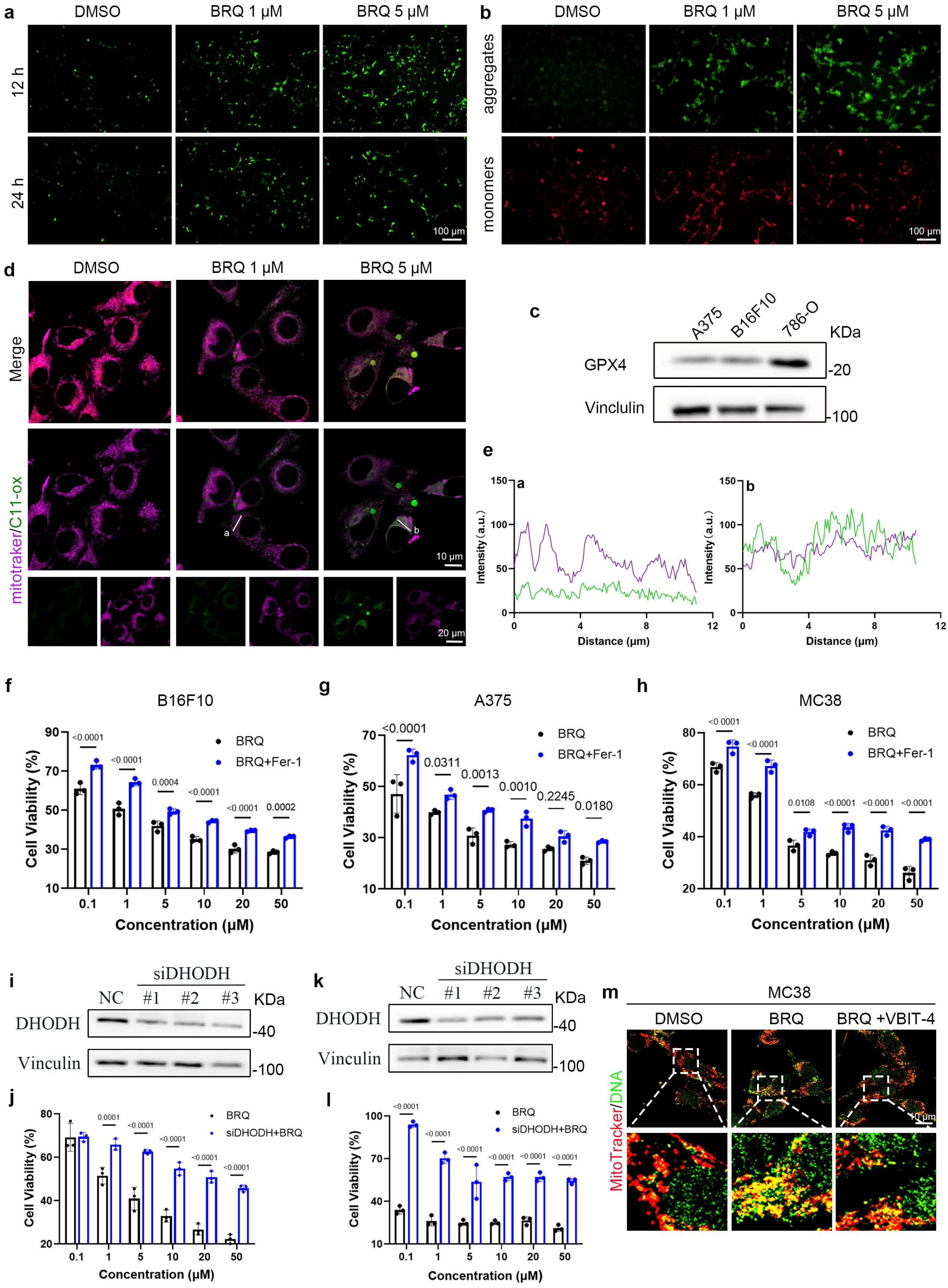

Fig. S5 BRQ induced mitochondrial oxidative stress and mtDNA released via VDAC. **a** ROS was

detected by DCFH-DA in B16F10 cells. **b** JC-1 analysis in B16F10 cells. **c** Western blot analysis of GPX4 in A375, B16F10 and 786-O cell lines. **d** Representative images of B16F10 cells co-stained BODIPY C11 (Oxidized BODIPY-green/non oxidized BODIPY-red) and mitochondrial (purple). **e** Colocalization analysis of mitochondrial and Oxidized BODIPY B16F10 cells. **f-h** Cell viability in (f) B16F10, (g) A375 and (h) MC38 cells treated with BRQ or BRQ+Fer-1 (n = 3). **i** Representative images of knocking down the expression level of DHODH in B16F10 cells. **j** Cell viability in B16F10 treated with BRQ or BRQ+siDHODH (n = 3). **k** Representative images of knocking down the expression level of DHODH in A375 cells. **l** Cell viability in A375 treated with BRQ or BRQ+siDHODH (n = 3). **m** CLSM images of mtDNA released from mitochondrial in MC38 (Red: mitochondrial; green: DNA).

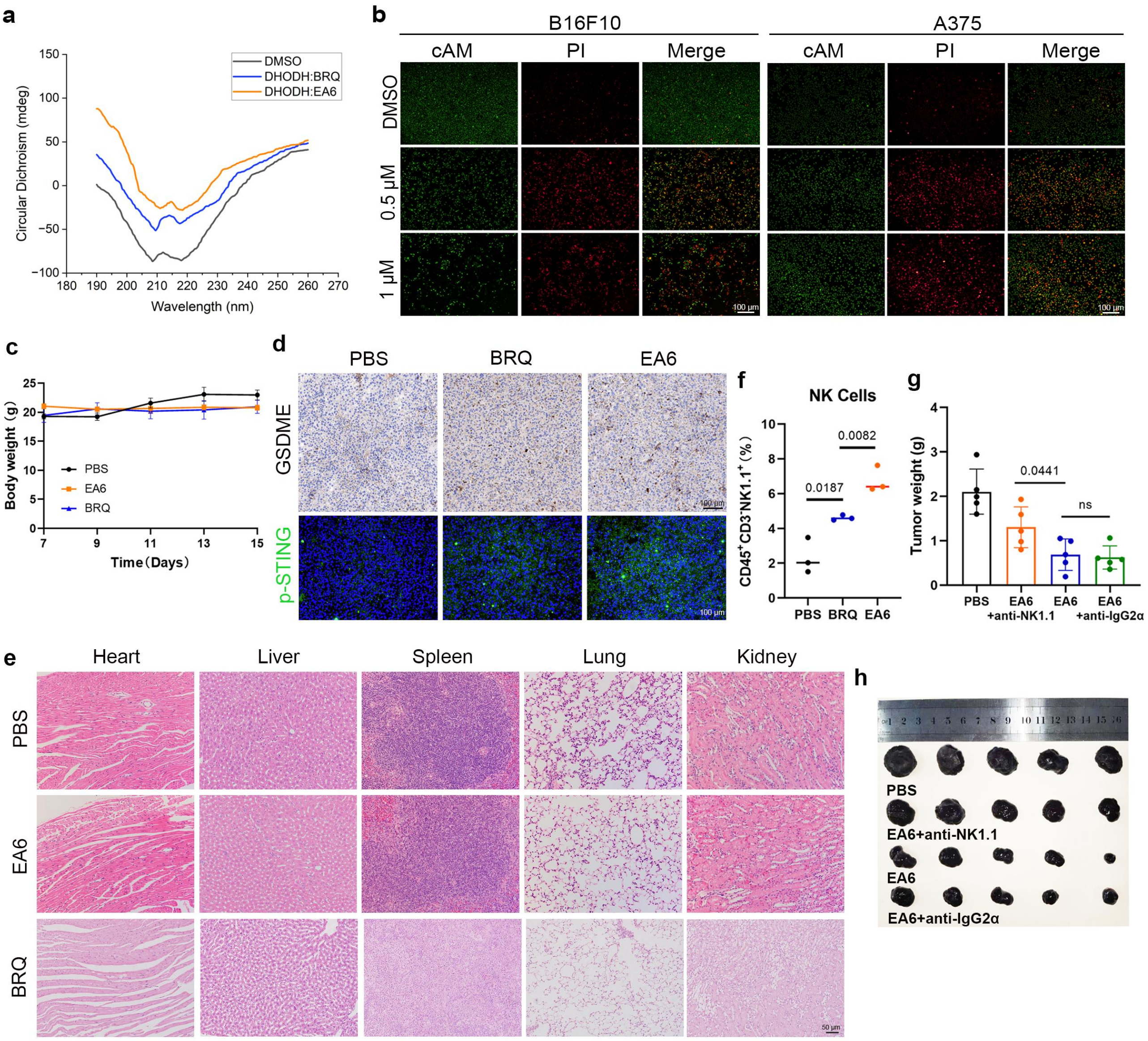

Fig. S6 EA6, a more effective DHODH inhibitor. **a** Representative data of BRQ or EA6 binding to DHODH detected by CD. Purified DHODH protein (20 μM) was incubated with 1 μM BRQ/EA6 and subjected to CD spectroscopy analysis. **b** Live/dead staining analysis in B16F10 and A375 cells. Red indicated dead cells (PI), green indicated live cells (cAM). **c** Body weight of mice (n = 5). **d** The expression of GSDEM and p-STING in indicted tumor tissue. **e** H&E stain of heart, liver, spleen, lung and kidney. **f** Quantitative graph of flow cytometry analysis for tumor-infiltrating NK cells. **g** The average tumor weight at the end of the experiment. **h** Images of isolated tumors for each group in NK-depletion experiment.

Table S1 IC_{50} values of drugs to variable cell lines at 72 h [a]

Compounds	R1	R2	B16F10
			IC_{50} μM
BQR	-	-	1.11±0.08
AA2	3,5-F	6-F	0.78±0.32
AA3	2-Cl	6-F	0.38±0.06
BA3	2-Cl	$6\text{-}OCH_3$	7.83±5.32
BA4	3-F	$6\text{-}OCH_3$	12.07±4.76
CA2	3,5-F	$6\text{-}CH_3$	1.82±0.41
CA4	3-F	$6\text{-}CH_3$	1.50±0.39
CA8	2,4-F	$6\text{-}CH_3$	3.09±0.56
EA2	3,5-F	6-Cl	1.62±0.64
EA3	2-Cl	6-Cl	0.3±0.07
EA4	3-F	6-Cl	1.62±0.84
EA6	$2\text{-}CH_3$	6-Cl	0.09±0.05
EA8	2,4-F	6-Cl	1.88±0.22
FA11	2,6-F	$7\text{-}OCH_3$	＞100
FA12	2-Cl-6-F	$7\text{-}OCH_3$	12.24±2.36
FA14	$2\text{-}F\text{-}5\text{-}OCH_3$	$7\text{-}OCH_3$	＞100
GA14	$2\text{-}F\text{-}5\text{-}OCH_3$	6,7-F	＞100

[a] IC_{50} values are represented by mean ± SD of three independent experiments.

Table S2 Detailed information of antibodies

Antibody	Supplier	Cat. Num.	Dilution
DHODH Polyclonal antibody	Proteintech	14877	1:2000
GSDME antibody	CST	40618	1:1000
Gasdermin D (E9S1X) Rabbit mAb	CST	39754	1:1000
Cleaved Gasdermin D (Asp276) Rabbit mAb	CST	10137	1:1000
Caspase-3(D3R6Y) Rabbit mAb	CST	14220	1:1000
Anti-Vinculin antibody	Abcam	129002	1:10000
STING(D1V5L) Rabbit mAb	CST	50494	1:1000
P-STING(S365) Rabbit mAb	CST	72971	1:1000
cGAS(D3080) Rabbit mAb	CST	31659	1:1000
TBK1/NAK(D1B4) Rabbit mAb	CST	3504	1:1000
p-TBK1/NAK(S172) Rabbit mAb	CST	5483	1:1000
IRF-3(D83B9) Rabbit mAb	CST	4302	1:1000
p-IRF-3(S396) Rabbit mAb	CST	29047	1:1000
P-STING(S365) Rabbit mAb	CST	50907	1:1000
Anti-Glutathione peroxidase 4 antibody	Abcam	125066	1:1000
Anti-beta Actin antibody (Mouse mAb)	ServiceBio	GB15001	1:3000
VDAC(D73D12) Rabbit mAb	CST	4661	1:1000
AIM2 antibody	CST	63660	1:1000
hnRNPA2B1 antibody	CST	25734	1:1000
Anti-cGAS antibody	Abcam	302617	1:1000

Antibody	Supplier	Cat. Num.	Dilution
PE Anti-Mouse CD80	Proteintech	PE-65076	1:200
APC Anti-Mouse CD86	Proteintech	APC-65068	1:500
CD8α (RPA-T8) Mouse mAb (APC Conjugate)	CST	64915	1:20
CD3 (UCHT1) Mouse mAb (PE Conjugate)	CST	46233	1:20
CD4(RM4-5) Rabbit mAb (FITC Conjugate)	CST	96127	1:200
PerCP/Cyanine5.5 anti-mouse CD45 Antibody	Biolegend	103132	1:80
PE anti-mouse NK1.1 Antibody	Biolegend	108707	1:80
DFNA5(G-9)	Santa	393162	1:500
Anti-NKR-P1C antibody	Abcam	289542	1:50
InvivoMAb mouse IgG2α isotype control	Biocell	BE0085	N/A
InvivoMAb anti-mouse NK1.1	Biocell	BE0036	N/A

Table S3 siRNA sequences targeting DHODH

Target	Sequence (5'-3')
Mouse si#1 sense	GACGGACUGAUCAUCACAA(dT)(dT)
Mouse si#1 antisense	UUGUGAUGAUCAGUCCGUC(dT)(dT)
Mouse si#2 sense	GGCUAGCUGUUCGAGUCAU(dT)(dT)
Mouse si#2 antisense	AUGACUCGAACAGCUAGCC(dT)(dT)
Mouse si#3 sense	GCUGUGGACGGACUCUAUA(dT)(dT)
Mouse si#3 antisense	UAUAGAGUCCGUCCACAGC(dT)(dT)
Human si#1 sense	GGUAUGGAUUUAACAGUCA(dT)(dT)
Human si#1 antisense	UGACUGUUAAAUCCAUACC(dT)(dT)
Human si#2 sense	GAUGUAUGCACUCACCCAA(dT)(dT)
Human si#2 antisense	UUGGGUGAGUGCAUACAUC(dT)(dT)
Human si#3 sense	GUUGAGAUAGGAAGUGUGA(dT)(dT)
Human si#3 antisense	UCACACUUCCUAUCUCAAC(dT)(dT)

Table S4 shRNA sequences targeting STING

Target	Sequence
Mouse shSTING#1	CCGGATGATTCTACTATCGTCTTATCTCGAGATAAGA CGATAGTAGAATCATTTTTT
Mouse shSTING#2	CCGGCAACATTCGATTCCGAGATATCTCGAGATATCT CGGAATCGAATGTTGTTTTT
Mouse shSTING#3	CCGGAGAGGTCACCGCTCCAAATATCTCGAGATATTT GGAGCGGTGACCTCTTTTTT

Table S5 shRNA sequences targeting GSDME

Target	Sequence
Mouse shGSDME#1	5'- GATGATGGAGTATCTGATCTT-3'
Mouse shGSDME#2	5'- GCGGTCCTATTTGATGATGAA-3'
Mouse shGSDME#3	5'- GCATGATGAATGACCTGACTT-3

Brequinar derivatives synthesis and characterization

Chemicals and Materials. All commercial reagents and synthesized materials obtained from Energy-Chemical, Adamas-Beta, or Topbiochem were used without purification unless otherwise specified. Flash-column chromatography was realized with 200-300 mesh silica gel (Qingdao Haiyang Chemical, China). ^{1}H and ^{13}C NMR spectra were recorded on a Bruker ARX 600 MHz spectrometer: chemical shifts and coupling constants (J) were shown in parts per million and in hertz, respectively. HRMS spectra were measured on a Bruker micrOTOF Q spectrometer.

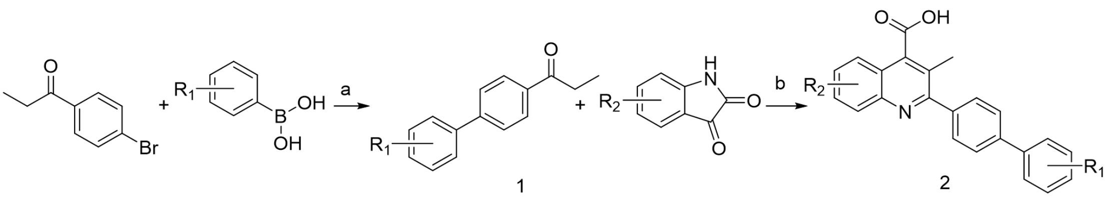

Reaction and conditions: a $(PPh_3)_4Pd$, K_2HPO_4, dioxane:H_2O 3:1; **b** KOH, EtOH:H_2O 3:1.

General synthesis for intermediate 1

1-(4-Bromophenyl) propan-1-one(1 equiv), the corresponding phenylboronic acid (1 equiv), $(PPH_3)_4Pd$ (0.05 equiv) and K_2HPO_4 (2 equiv) were added in 3:1 1,4- dioxane / H_2O under inert atmosphere . The reaction was heated to 130 ^{o}C for reflux for 24 h. After the reaction was complete, the obtained mixture was concentrated, and the mixture was extracted with ethyl acetate three times (3×). The organic layer was dried with MgSO4. The residue was purified by column chromatography.

General synthesis for product 2

The corresponding isatin, KOH, were added in 3:1 EtOH/H_2O. The reaction was heated to 100 ^{o}C for reflux for 0.5 h. The product from the first step was added. The reaction was heated to 80 ^{o}C for reflux for 9 h. The mixture was concentrated, and the mixture was extracted with ethyl acetate three times (3×). The aqueous layer was acidified with HCl until pH 2-3 was reached, the sediment is

filtered and washed in deionized water and product was dried under vacuum.

1-(3',5'-difluoro-[1,1'-biphenyl]-4-yl)propan-1-one

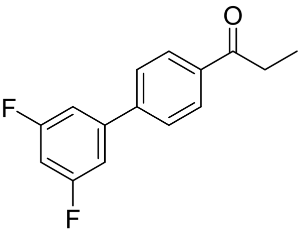

A2 The synthesis of general synthesis for intermediate 1, white solid, 75%. ^{1}H NMR (62 MHz, DMSO-*d6*): δ 8.24 – 7.74 (m, 4H), 7.71 – 7.53 (m, 1H), 7.52 – 7.04 (m, 2H), 3.10 (q, *J* = 7.1 Hz, 2H), 1.11 (t, *J* = 7.1 Hz, 3H). ^{13}C NMR (101 MHz, DMSO-*d6*): δ 200.45, 164.65, 164.51, 162.20, 162.07, 143.08, 142.98, 142.88, 142.06, 142.04, 142.01, 136.85, 128.96, 127.67, 110.80, 110.73, 110.61, 110.54, 104.30, 104.05, 103.79, 31.83, 8.53. HRMS (ESI) calcd for $C_{15}H_{12}F_2O$ $[M+H]^+$ 247.0934, found 247.0928.

1-(2'-chloro-[1,1'-biphenyl]-4-yl)propan-1-one

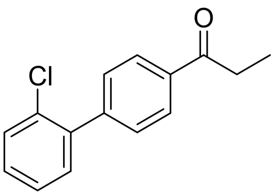

A3 The synthesis of general synthesis for intermediate 1, white solid, 77%. ^{1}H NMR (62 MHz, DMSO-*d6*): δ 8.24 – 7.87 (m, 2H), 7.75 – 7.29 (m, 6H), 3.10 (q, *J* = 7.1 Hz, 2H), 1.11 (t, *J* = 7.1 Hz, 3H). ^{13}C NMR (101 MHz, DMSO-*d6*): δ 200.52, 143.54, 139.34, 136.25, 131.84, 131.62, 130.43, 130.25, 130.08, 128.23, 128.11, 31.78, 8.57. HRMS (ESI) calcd for $C_{15}H_{13}ClO$ $[M+H]^+$ 245.0733, found 245.0728.

1-(3'-fluoro-[1,1'-biphenyl]-4-yl)propan-1-one

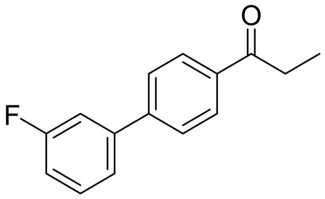

A4 The synthesis of general synthesis for intermediate 1, white solid, 79%. ^{1}H NMR (62 MHz, DMSO-*d6*): δ 8.26 – 6.98 (m, 8H), 3.08 (q, *J* = 7.1 Hz, 2H), 1.10 (t, *J* = 7.1 Hz, 3H). ^{13}C NMR (101 MHz, DMSO-*d6*): δ 200.43, 164.39, 161.97, 143.31, 143.29, 141.87, 141.80, 136.37, 131.54, 131.45, 128.99, 127.53, 123.55, 123.52, 115.62, 115.41, 114.30, 114.08, 31.78, 8.56. HRMS (ESI) calcd for $C_{15}H_{13}ClO$ $[M+H]^+$ 229.1029, found 229.1031.

1-(2'-methyl-[1,1'-biphenyl]-4-yl)propan-1-one

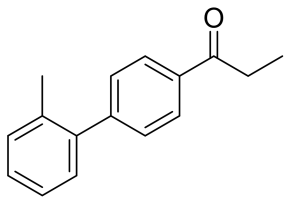

A6 The synthesis of general synthesis for intermediate 1, white solid, 79%. ^{1}H NMR (500 MHz, DMSO-*d6*): δ 8.03 (d, J = 8.3 Hz, 2H), 7.49 (d, J = 8.3 Hz, 2H), 7.34 – 7.26 (m, 3H), 7.24 – 7.21 (m, 1H), 3.09 (q, J = 7.2 Hz, 2H), 2.24 (s, 3H), 1.11 (t, J = 7.2 Hz, 3H).8.03 (d, J = 8.3 Hz, 2H), 7.49 (d, J = 8.3 Hz, 2H), 7.34 – 7.26 (m, 3H), 7.24 – 7.21 (m, 1H), 3.09 (q, J = 7.2 Hz, 2H), 2.24 (s, 3H), 1.11 (t, J = 7.2 Hz, 3H). ^{13}C NMR (101 MHz, DMSO-*d6*) δ: 200.46, 146.33, 140.76, 135.59, 135.13, 130.96, 129.80, 129.77, 128.34, 128.28, 126.53, 31.69, 20.55, 8.59. HRMS (ESI) calcd for $C_{16}H_{16}O$ $[M+H]^+$ 225.1279, found 225.1269.

1-(2',4'-difluoro-[1,1'-biphenyl]-4-yl)propan-1-one

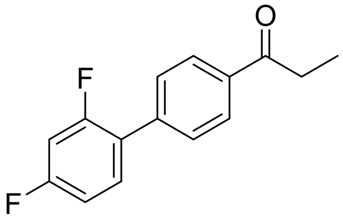

A8 The synthesis of general synthesis for intermediate 1, white solid, 79%. ^{1}H NMR (62 MHz, DMSO-*d6*): δ 8.26 – 7.91 (m, 2H), 7.88 – 7.00 (m, 5H), 3.09 (q, *J* = 7.1 Hz, 2H), 1.10 (t, *J* = 7.1 Hz, 3H). ^{13}C NMR (101 MHz, DMSO-*d6*): δ 200.47, 163.94, 163.82, 161.48, 161.35, 160.97, 160.85, 158.49, 158.37, 139.07, 139.05, 136.26, 132.57, 132.52, 132.47, 132.43, 129.52, 129.49, 128.63, 124.44, 124.41, 124.31, 124.28, 112.87, 112.83, 112.66, 112.62, 105.43, 105.17, 104.91, 31.78, 8.55. HRMS (ESI) calcd for $C_{15}H_{12}F_2O$ $[M+H]^+$ 247.0934, found 247.0931

1-(2',6'-difluoro-[1,1'-biphenyl]-4-yl)propan-1-one

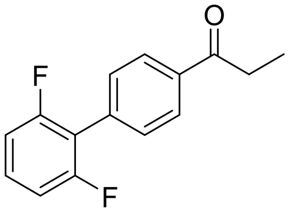

A11 The synthesis of general synthesis for intermediate 1, white solid, 72%. ^{1}H NMR (62 MHz, DMSO-*d6*):δ 8.21 – 7.94 (m, 2H), 7.77 – 7.03 (m, 5H), 3.10 (q, J = 7.1 Hz, 2H), 1.11 (t, J = 7.1 Hz, 3H). ^{13}C NMR (101 MHz, DMSO-*d6*): δ 200.52, 160.95, 160.88, 158.49, 158.42, 136.80, 133.55, 131.29, 131.18, 131.08, 130.98, 130.96, 130.94, 128.36, 117.51, 117.32, 117.14, 112.76, 112.70, 112.57, 112.51, 31.79, 8.52.

HRMS (ESI) calcd for $C_{15}H_{12}F_2O$ $[M+H]^+$ 247.0934, found 247.0916.

1-(2'-chloro-6'-fluoro-[1,1'-biphenyl]-4-yl)propan-1-one

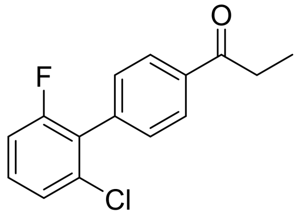

A12 The synthesis of general synthesis for intermediate 1, white solid, 72%. ^{1}H NMR (400 MHz, DMSO-*d*6): δ 8.07 (d, J = 8.6 Hz, 2H), 7.53 – 7.43 (m, 4H), 7.38 – 7.31 (m, 1H), 3.09 (q, J = 7.1 Hz, 2H), 1.11 (t, J = 7.1 Hz, 3H). ^{13}C NMR (101 MHz, DMSO-*d*6): δ 200.40, 161.17, 158.71, 137.01, 136.83, 133.45, 133.41, 131.21, 131.12, 130.84, 130.82, 128.25, 127.99, 127.80, 126.21, 126.18, 115.41, 115.19, 31.77, 8.46. HRMS (ESI) calcd for $C_{15}H_{12}ClFO$ $[M+H]^+$ 263.0639, found 263.0616.

1-(2'-chloro-6'-fluoro-[1,1'-biphenyl]-4-yl)propan-1-one

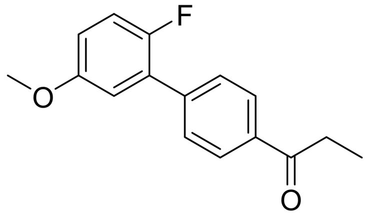

A14 The synthesis of general synthesis for intermediate 1, white solid, 79%. ^{1}H NMR (62 MHz, DMSO-*d*6): δ 8.06 (d, *J* = 8.5 Hz, 2H), 7.70 (m, 2H), 7.48 – 6.82 (m, 3H), 3.81 (s, 3H), 3.09 (q, *J* = 7.1 Hz, 2H), 1.11 (t, *J* = 7.1 Hz, 3H). ^{13}C NMR (101 MHz, DMSO-*d*6): δ 200.52, 156.24, 156.23, 155.03, 152.66, 139.95, 139.94, 136.28, 129.60, 129.57, 128.55, 128.28, 128.14, 117.57, 117.33, 115.83, 115.75, 115.63, 115.60, 56.21, 31.79, 8.57. HRMS (ESI) calcd for $C_{16}H_{15}FO_2$ $[M+H]^+$ 259.1134, found 259.1122.

2-(3',5'-difluoro-[1,1'-biphenyl]-4-yl)-6-fluoro-3-methylquinoline-4-carboxylic acid

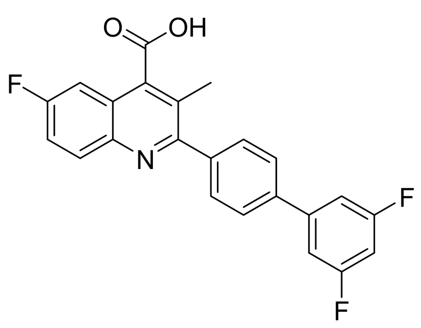

AA2 The synthesis of General synthesis for product 2, white solid, 55%. ^{1}H NMR (400 MHz, DMSO-*d*6): δ 8.01 (dd, J = 9.3, 5.6 Hz, 1H), 7.88 (d, J = 8.4 Hz, 2H), 7.68 (d, J = 8.4 Hz, 2H), 7.65 – 7.50 (m, 4H), 7.27 (tt, J = 9.3, 2.4 Hz, 1H), 2.38 (s, 3H). ^{13}C NMR (101 MHz, DMSO-*d*6): δ 164.69, 164.55, 162.25, 162.11, 161.26, 159.47, 158.84, 143.92, 143.82, 143.72,

143.59, 141.50, 137.64, 132.07, 131.97, 130.17, 127.06, 124.58, 124.48, 123.23, 119.20, 118.95, 110.46, 110.40, 110.28, 110.21, 109.90, 109.67, 103.64, 103.38, 103.13, 18.08. 394.1148. HRMS (ESI) calcd for $C_{23}H_{14}F_3NO_2$ $[M+H]^+$ 394.1055, found 394.1148. HPLC purity at 254 nm, 99.52%.

2-(2'-chloro-[1,1'-biphenyl]-4-yl)-6-fluoro-3-methylquinoline-4-carboxylic acid

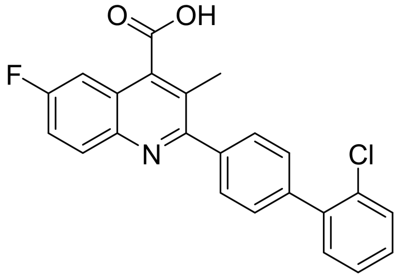

AA3 The synthesis of General synthesis for product 2, yellow solid, 50%. ^1H NMR (400 MHz, DMSO-*d6*): δ 8.02 (dd, *J* = 9.2, 5.8 Hz, 1H), 7.67 (d, *J* = 8.2 Hz, 2H), 7.65 – 7.58 (m, 3H), 7.56 (d, *J* = 8.2 Hz, 2H), 7.51 (dd, *J* = 7.2, 2.4 Hz, 1H), 7.45 (dtd, *J* = 12.9, 7.2, 1.9 Hz, 2H), 2.41 (s, 3H). ^{13}C NMR (101 MHz, DMSO-*d6*): δ 170.66, 161.32, 159.71, 159.68, 158.89, 143.59, 140.53, 139.92, 138.87, 132.12, 132.03, 131.83, 130.41, 129.82, 129.40, 129.34, 128.09, 124.48, 124.38, 123.41, 119.27, 119.02, 109.73, 109.50, 18.11. HRMS (ESI) calcd for $C_{23}H_{15}ClFNO_2$ $[M+H]^+$ 392.0854, found 392.0832. HPLC purity at 254 nm, 99.16%

2-(2'-chloro-[1,1'-biphenyl]-4-yl)-6-methoxy-3-methylquinoline-4-carboxylic acid

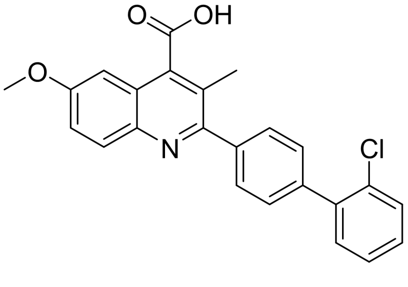

BA3 The synthesis of General synthesis for product 2, yellow solid, 44%. ^1H NMR (400 MHz, DMSO-*d6*): δ 7.94 (d, *J* = 9.2 Hz, 1H), 7.71 – 7.67 (m, 2H), 7.61 (dd, *J* = 7.1, 2.1 Hz, 1H), 7.58 – 7.55 (m, 2H), 7.54 – 7.39 (m, 5H), 7.13 (d, *J* = 2.8 Hz, 1H), 3.89 (s, 3H), 2.41 (s, 3H). ^{13}C NMR (101 MHz, DMSO-*d6*): δ 169.94, 158.14, 157.44, 142.41, 140.30, 139.92, 138.84, 132.04, 131.83, 131.16, 130.41, 129.82, 129.46, 129.42, 128.09, 124.20, 124.03, 121.99, 103.23, 55.93, 18.09. HRMS (ESI) calcd for $C_{24}H_{18}ClNO_3$ $[M+H]^+$ 404.1053, found 404.1037. HPLC purity at 254 nm, 98.01%.

2-(3'-fluoro-[1,1'-biphenyl]-4-yl)-6-methoxy-3-methylquinoline-4-carboxylic acid

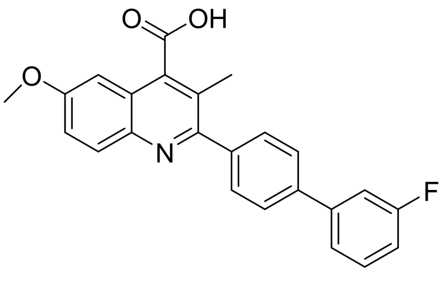

BA4 The synthesis of General synthesis for product 2, white solid, 47%. ^{1}H NMR (400 MHz, DMSO-*d6*): δ 7.98 (d, *J* = 9.2 Hz, 1H), 7.87 – 7.83 (m, 2H), 7.72 (dt, *J* = 6.4, 1.9 Hz, 2H), 7.62 (ddd, *J* = 7.5, 4.2, 1.8 Hz, 2H), 7.55 (td, *J* = 8.1, 6.2 Hz, 1H), 7.45 (dd, *J* = 9.2, 2.7 Hz, 1H), 7.27 – 7.21 (m, 1H), 7.08 (d, *J* = 2.7 Hz, 1H), 3.91 (s, 3H), 2.43 (s, 3H).^{13}C NMR (101 MHz, DMSO-*d6*): δ 169.50, 167.43, 164.45, 162.03, 158.43, 157.35, 142.64, 142.56, 142.37, 140.67, 140.33, 138.94, 138.92, 132.19, 131.98, 131.46, 131.38, 130.29, 129.13, 127.01, 124.68, 124.01, 123.31, 123.29, 122.23, 114.97, 114.76, 114.05, 113.83, 102.72, 55.96, 18.11. HRMS (ESI) calcd for $C_{24}H_{18}FNO_3$ $[M+H]^+$ 388.1349, found 388.1325. HPLC purity at 254 nm, 91.66%.

2-(3',5'-difluoro-[1,1'-biphenyl]-4-yl)-3,6-dimethylquinoline-4-carboxylic acid

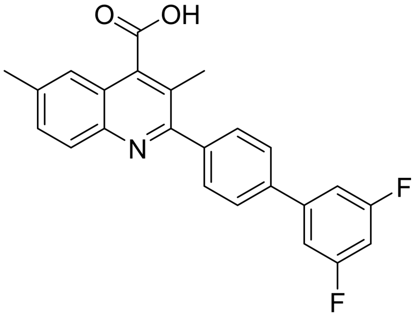

CA2 The synthesis of General synthesis for product 2, white solid, 50%. ^{1}H NMR (400 MHz, DMSO-*d6*): δ 7.96 (d, *J* = 8.5 Hz, 1H), 7.91 – 7.87 (m, 2H), 7.75 – 7.71 (m, 2H), 7.62 (dd, *J* = 8.7, 1.9 Hz, 1H), 7.58 – 7.53 (m, 3H), 7.27 (tt, *J* = 9.3, 2.3 Hz, 1H), 2.55 – 2.53 (m, 3H), 2.43 (s, 3H). ^{13}C NMR (101 MHz, DMSO-*d6*): δ 169.47, 164.69, 164.55, 162.25, 162.11, 158.90, 144.83, 143.84, 143.75, 143.65, 141.18, 140.90, 137.89, 137.86, 137.84, 137.68, 132.12, 131.97, 130.28, 129.48, 129.13, 127.12, 124.22, 123.46, 122.93, 110.49, 110.42, 110.31, 110.24, 103.68, 103.42, 103.17, 21.87, 18.01. HRMS (ESI) calcd for $C_{24}H_{17}F_2NO_2$ $[M+H]^+$ 390.1306, found 390.1293. HPLC purity at 254 nm, 89.85%.

2-(3'-fluoro-[1,1'-biphenyl]-4-yl)-3,6-dimethylquinoline-4-carboxylic acid

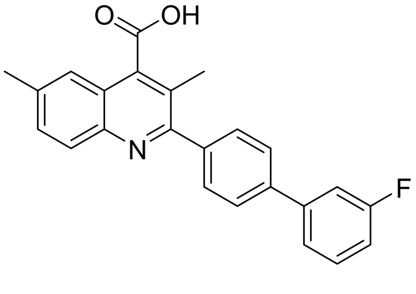

CA4 The synthesis of General synthesis for product 2, yellow solid,

59%. ^{1}H NMR (400 MHz, DMSO-*d6*): δ 7.82 (dd, *J* = 8.4, 1.5 Hz, 3H), 7.69 – 7.65 (m, 3H), 7.61 (ddd, *J* = 7.7, 4.0, 1.8 Hz, 2H), 7.55 (td, *J* = 8.0, 6.1 Hz, 1H), 7.49 (dd, *J* = 8.6, 2.0 Hz, 1H), 7.26 – 7.21 (m, 1H), 2.47 (s, 3H), 2.36 (s, 3H). ^{13}C NMR (101 MHz, DMSO-*d6*): δ 171.13, 164.45, 162.03, 158.99, 145.05, 142.77, 142.69, 141.41, 138.56, 135.39, 131.46, 131.37, 131.01, 130.17, 128.90, 126.88, 125.59, 123.73, 123.28, 123.25, 121.73, 114.88, 114.67, 114.00, 113.78, 21.83, 18.04. HRMS (ESI) calcd for $C_{24}H_{18}FNO_2$ $[M+H]^+$ 372.1400, found 372.1388. HPLC purity at 254 nm, 96.11%.

2-(2',4'-difluoro-[1,1'-biphenyl]-4-yl)-3,6-dimethylquinoline-4-carboxylic acid

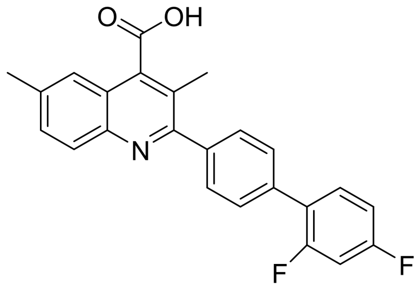

CA8 The synthesis of General synthesis for product 2, yellow solid, 54%. ^{1}H NMR (400 MHz, DMSO-*d6*): δ 7.95 (d, *J* = 8.5 Hz, 1H), 7.74 – 7.72 (m, 2H), 7.67 (dq, *J* = 8.9, 1.7 Hz, 3H), 7.63 (dd, *J* = 8.7, 1.9 Hz, 1H), 7.56 (t, *J* = 1.4 Hz, 1H), 7.42 (ddd, *J* = 11.6, 9.3, 2.6 Hz, 1H), 7.27 – 7.22 (m, 1H), 2.54 (s, 3H), 2.43 (s, 3H). ^{13}C NMR (101 MHz, DMSO-*d6*): δ 169.49, 167.43, 159.03, 144.83, 140.19, 137.63, 134.62, 132.53, 132.48, 132.43, 132.38, 132.12, 131.99, 129.91, 129.46, 129.14, 128.99, 128.96, 125.01, 124.88, 124.16, 123.49, 122.92, 112.79, 112.58, 105.36, 105.10, 104.84, 21.87, 18.04. HRMS (ESI) calcd for $C_{24}H_{17}F_2NO_2$ $[M+H]^+$ 390.1306, found 390.1292. HPLC purity at 254 nm, 97.10%.

6-chloro-2-(3',5'-difluoro-[1,1'-biphenyl]-4-yl)-3-methylquinoline-4-carboxylic acid

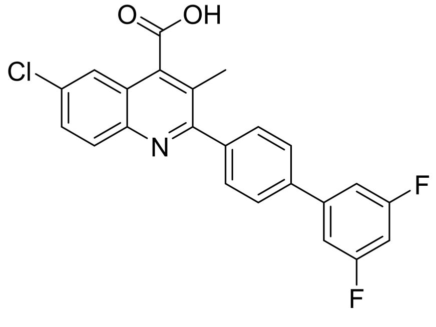

EA2 The synthesis of General synthesis for product 2, white solid, 54%. ^{1}H NMR (400 MHz, DMSO-*d6*): δ 8.00 (d, *J* = 2.4 Hz, 1H), 7.95 (d, *J* = 9.0 Hz, 1H), 7.89 – 7.85 (m, 2H), 7.69 – 7.64 (m, 3H), 7.54 (dt, *J* = 7.3, 2.2 Hz, 2H), 7.27 (tt, *J* = 9.3, 2.3 Hz, 1H),

2.38 (s, 3H). ^{13}C NMR (101 MHz, DMSO-*d*6): δ 170.80, 164.68, 164.55, 162.24, 162.11, 160.44, 149.43, 144.81, 143.89, 143.79, 143.70, 141.52, 137.66, 131.29, 130.63, 130.15, 129.37, 127.04, 125.75, 124.69, 123.03, 110.45, 110.38, 110.27, 110.20, 103.65, 103.39, 103.14, 18.09. HRMS (ESI) calcd for $C_{23}H_{14}ClF_2NO_2$ [M-H]$^-$ 408.0603, found 408.0576. HPLC purity at 254 nm, 99.21%.

6-chloro-2-(2'-chloro-[1,1'-biphenyl]-4-yl)-3-methylquinoline-4-carboxylic acid

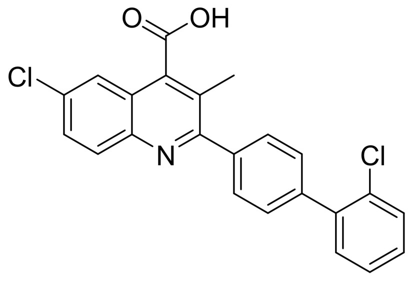

EA3 The synthesis of General synthesis for product 2, white solid, 64%. ^{1}H NMR (400 MHz, DMSO-*d*6): δ 7.97 (d, J = 2.4 Hz, 1H), 7.94 (d, J = 8.9 Hz, 1H), 7.69 – 7.63 (m, 3H), 7.61 (dd, J = 7.3, 1.9 Hz, 1H), 7.59 – 7.54 (m, 2H), 7.53 – 7.43 (m, 3H), 2.40 (s, 3H). ^{13}C NMR (101 MHz, DMSO-*d*6): δ 170.45, 160.70, 144.85, 140.75, 139.94, 138.84, 132.03, 131.82, 131.24, 130.42, 129.83, 129.38, 129.32, 129.25, 128.10, 125.88, 124.77, 122.70, 18.10. HRMS (ESI) calcd for $C_{23}H_{15}Cl_2NO_2$ [M-H]$^-$ 406.0402, found 406.0362. HPLC purity at 254 nm, 97.17%.

6-chloro-2-(3'-fluoro-[1,1'-biphenyl]-4-yl)-3-methylquinoline-4-carboxylic acid

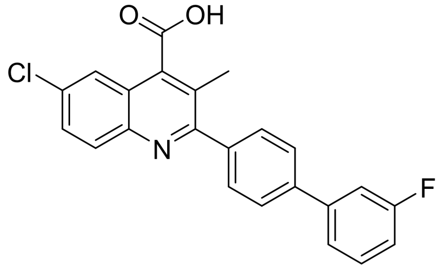

EA4 The synthesis of General synthesis for product 2, white solid, 64%. ^{1}H NMR (400 MHz, DMSO-*d*6): δ 8.13 – 8.08 (m, 1H), 7.87 (d, J = 8.4 Hz, 2H), 7.82 (d, J = 8.5 Hz, 2H), 7.75 (d, J = 8.3 Hz, 2H), 7.66 – 7.61 (m, 2H), 7.55 (td, J = 7.8, 5.9 Hz, 1H), 7.28 – 7.22 (m, 1H), 2.46 (s, 3H). ^{13}C NMR (101 MHz, DMSO-*d*6): δ 168.78, 164.45, 162.03, 160.73, 144.61, 142.52, 142.45, 140.66, 139.83, 139.36, 139.34, 132.53, 131.99, 131.49, 131.40, 130.56, 130.26, 127.09, 126.07, 123.68, 123.44, 123.36, 123.34, 115.07, 114.86, 114.10, 113.88, 18.25. HRMS (ESI) calcd for $C_{23}H_{15}ClFNO_2$ [M+H]$^+$ 392.0854, found 392.0846. HPLC purity at 254 nm, 89.58%.

6-chloro-3-methyl-2-(2'-methyl-[1,1'-biphenyl]-4-yl)quinoline-4-carboxylic acid

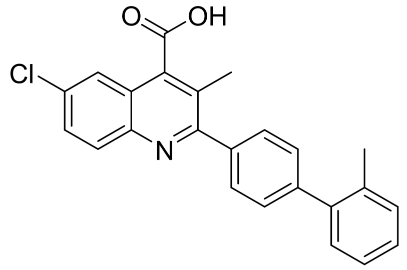

EA6 The synthesis of General synthesis for product 2, white solid, 49%. ^{1}H NMR (500 MHz, DMSO-*d*6): δ 8.12 (d, J = 8.8 Hz, 1H), 7.84 (dd, J = 8.8, 2.3 Hz, 1H), 7.81 (d, J = 2.3 Hz, 1H), 7.73 – 7.70 (m, 2H), 7.52 – 7.49 (m, 2H), 7.36 – 7.29 (m, 4H), 2.49 (s, 3H), 2.32 (s, 3H). ^{13}C NMR (101 MHz, DMSO-*d*6): δ 170.82, 160.87, 149.91, 144.84, 141.45, 141.38, 139.89, 135.26, 131.23, 130.90, 130.41, 130.01, 129.32, 129.24, 129.08, 127.94, 126.51, 125.86, 124.72, 122.88, 20.73, 18.15. HRMS (ESI) calcd for $C_{24}H_{18}ClNO_2$ $[M-H]^-$ 386.0948, found 386.0900. HPLC purity at 254 nm, 94.94%.

6-chloro-2-(2',4'-difluoro-[1,1'-biphenyl]-4-yl)-3-methylquinoline-4-carboxylic acid

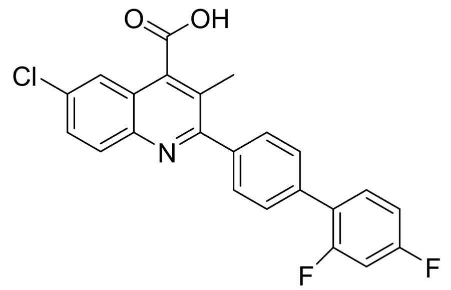

EA8 The synthesis of General synthesis for product 2, white solid, 49%. ^{1}H NMR (400 MHz, DMSO-*d*6): 8.10 (d, *J* = 8.8 Hz, 1H), 7.84 – 7.80 (m, 2H), 7.75 (d, *J* = 8.3 Hz, 2H), 7.69 (ddt, *J* = 8.8, 4.0, 2.3 Hz, 3H), 7.42 (ddd, *J* = 11.5, 9.3, 2.6 Hz, 1H), 7.28 – 7.22 (m, 1H), 2.47 (s, 3H). ^{13}C NMR (101 MHz, DMSO-*d*6): δ 168.73, 161.08, 160.73, 144.60, 140.56, 139.69, 134.96, 132.58, 132.50, 132.00, 130.60, 129.90, 129.06, 129.03, 126.10, 123.67, 123.42, 112.77, 112.56, 105.37, 105.11, 104.85, 18.25. HRMS (ESI) calcd for $C_{23}H_{14}ClF_2NO_2$ $[M+H]^+$ 410.0759, found 410.0722. HPLC purity at 254 nm, 97.28%.

2-(2',6'-difluoro-[1,1'-biphenyl]-4-yl)-7-methoxy-3-methylquinoline-4-carboxylic acid

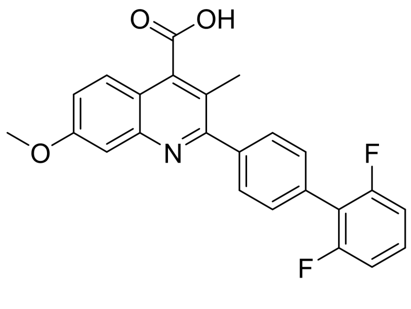

FA11 The synthesis of General synthesis for product 2, white solid, 46%. ^{1}H NMR (400 MHz, DMSO-*d*6): δ 14.19 (s, 1H), 7.77 – 7.69 (m, 3H), 7.68 – 7.65 (m, 1H), 7.61 (d, *J* = 7.9 Hz, 1H), 7.52 (dt, *J* =

8.2, 1.8 Hz, 1H), 7.45 (t, *J* = 2.2 Hz, 1H), 7.33 (dt, *J* = 9.1, 2.8 Hz, 1H), 7.28 (t, *J* = 8.0 Hz, 1H), 7.02 – 6.90 (m, 1H), 3.93 (s, 3H), 2.41 (s, 3H). ^{13}C NMR (101 MHz, DMSO-*d6*): δ 172.47, 169.44, 167.43, 160.54, 160.51, 160.08, 147.91, 140.77, 131.99, 131.77, 130.68, 130.34, 129.64, 129.14, 129.04, 128.98, 126.10, 121.78, 120.86, 120.75, 118.01, 117.94, 112.75, 112.49, 107.94, 56.02, 17.74. HRMS (ESI) calcd for $C_{24}H_{17}F_2NO_3$ $[M+H]^+$ 406.1255, found 406.1239. HPLC purity at 254 nm, 96.21%.

2-(2'-chloro-6'-fluoro-[1,1'-biphenyl]-4-yl)-7-methoxy-3-methylquinoline-4-carboxylic acid

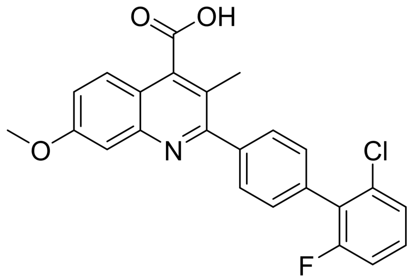

FA12 The synthesis of General synthesis for product 2, white solid, 59%. ^{1}H NMR (400 MHz, DMSO-*d6*): δ 7.75 (d, J = 9.1 Hz, 1H), 7.68 (q, J = 2.8, 2.1 Hz, 4H), 7.34 (d, J = 2.6 Hz, 1H), 7.31 – 7.26 (m, 1H), 7.17 (dd, J = 9.1, 2.6 Hz, 1H), 7.15 – 7.12 (m, 1H), 7.01 – 6.97 (m, 1H), 3.90 (s, 3H), 3.83 (s, 3H), 2.35 (s, 3H). ^{13}C NMR (101 MHz, DMSO-*d6*): δ 168.73, 160.73, 144.60, 140.56, 139.69, 134.96, 132.58, 132.00, 130.60, 129.90, 129.06, 129.03, 126.10, 123.67, 123.42, 112.77, 112.56, 105.37, 105.11, 104.85, 18.25. HRMS (ESI) calcd for $C_{24}H_{17}ClFNO_3$ $[M+H]^+$ 422.0959, found 422.0939. HPLC purity at 254 nm, 95.16%.

2-(2'-fluoro-5'-methoxy-[1,1'-biphenyl]-4-yl)-7-methoxy-3-methylquinoline-4-carboxylic acid

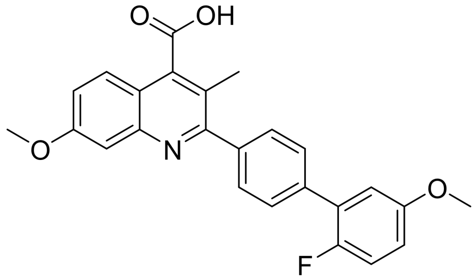

FA14 The synthesis of General synthesis for product 2, white solid, 70%. ^{1}H NMR (400 MHz, DMSO-*d6*): δ 7.75 (d, J = 9.1 Hz, 1H), 7.68 (q, J = 2.8, 2.1 Hz, 4H), 7.34 (d, J = 2.6 Hz, 1H), 7.31 – 7.26 (m, 1H), 7.17 (dd, J = 9.1, 2.6 Hz, 1H), 7.15 – 7.12 (m, 1H), 7.01 – 6.97 (m, 1H), 3.90 (s, 3H), 3.83 (s, 3H), 2.35 (s, 3H). ^{13}C NMR (101 MHz, DMSO-*d6*): δ 171.00, 159.99, 156.25, 156.23, 155.15, 152.78, 147.97, 141.12, 135.12, 129.72,

128.88, 128.85, 127.71, 120.15, 119.28, 118.69, 117.48, 117.23, 115.60, 115.57, 115.20, 115.13, 107.44, 56.18, 55.82, 17.77. HRMS (ESI) calcd for $C_{25}H_{20}FNO_4$ $[M+H]^+$ 418.1455, found 418.1427. HPLC purity at 254 nm, 97.53%.

6,7-difluoro-2-(2'-fluoro-5'-methoxy-[1,1'-biphenyl]-4-yl)-3-methylquinoline-4-carboxylic acid

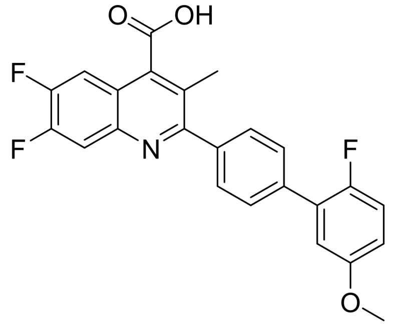

GA14 The synthesis of General synthesis for product 2, white solid, 60%. ^{1}H NMR (400 MHz, DMSO-*d6*): δ 7.71 – 7.65 (m, 4H), 7.56 (d, J = 12.4 Hz, 1H), 7.52 (d, J = 8.5 Hz, 1H), 7.28 (dd, J = 10.3, 9.0 Hz, 1H), 7.13 (dd, J = 6.4, 3.2 Hz, 1H), 6.99 (dt, J = 9.0, 3.5 Hz, 1H), 3.83 (s, 3H), 2.36 (s, 3H). ^{13}C NMR (101 MHz, DMSO-*d6*): δ 170.08, 159.53, 156.24, 155.15, 153.25, 152.78, 150.77, 149.20, 149.07, 144.59, 140.73, 135.24, 129.73, 128.92, 128.89, 128.77, 121.59, 118.00, 117.92, 117.49, 117.24, 115.60, 115.57, 115.24, 115.16, 110.91, 109.80, 56.18, 17.86. HPLC purity at 254 nm, 96.00%.

NMR Spectrum

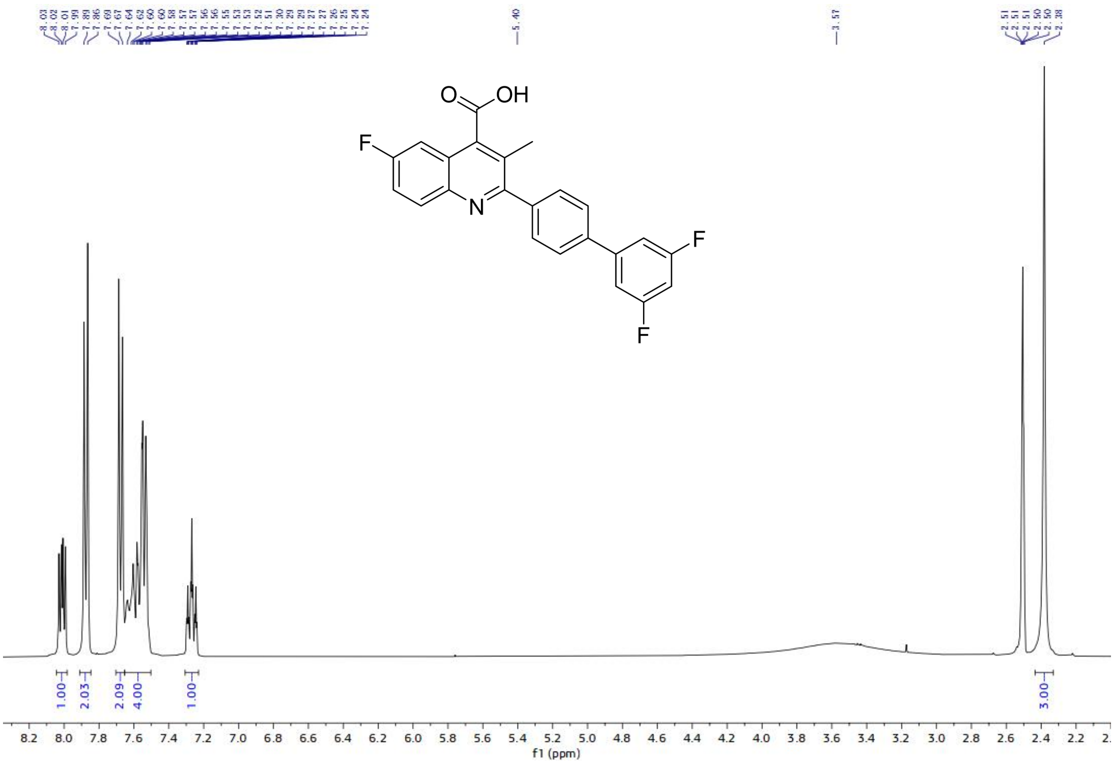

^{1}H-NMR spectrum of AA2

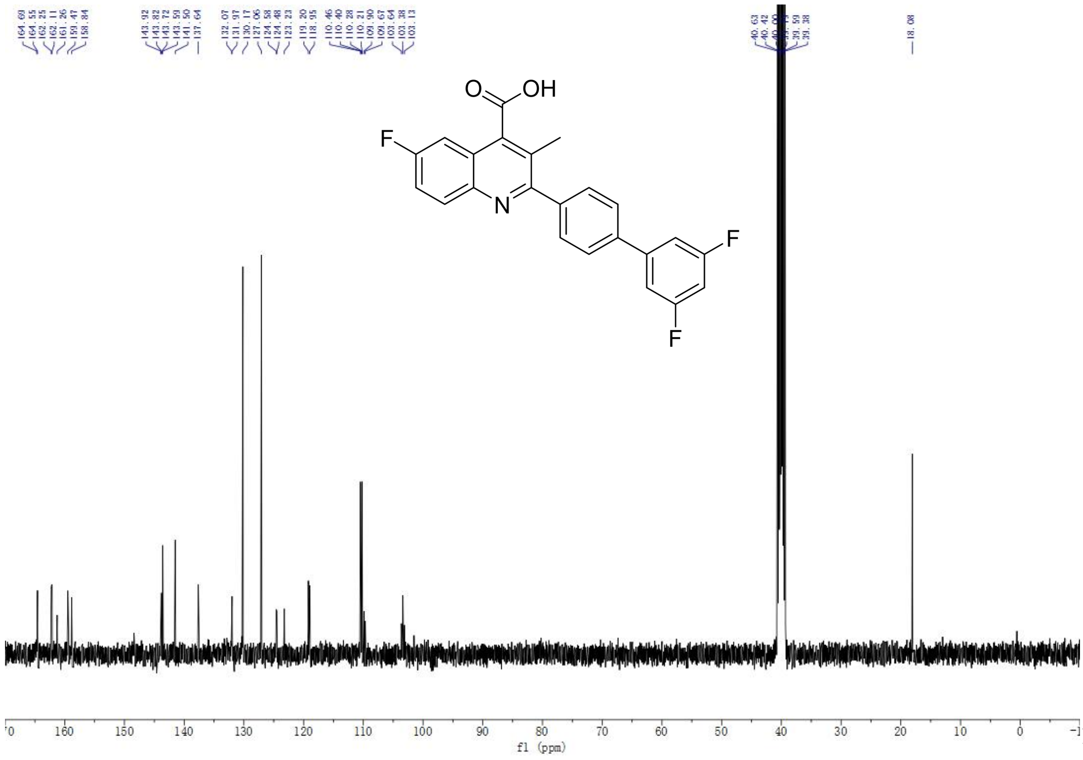

^{13}C-NMR spectrum of AA3

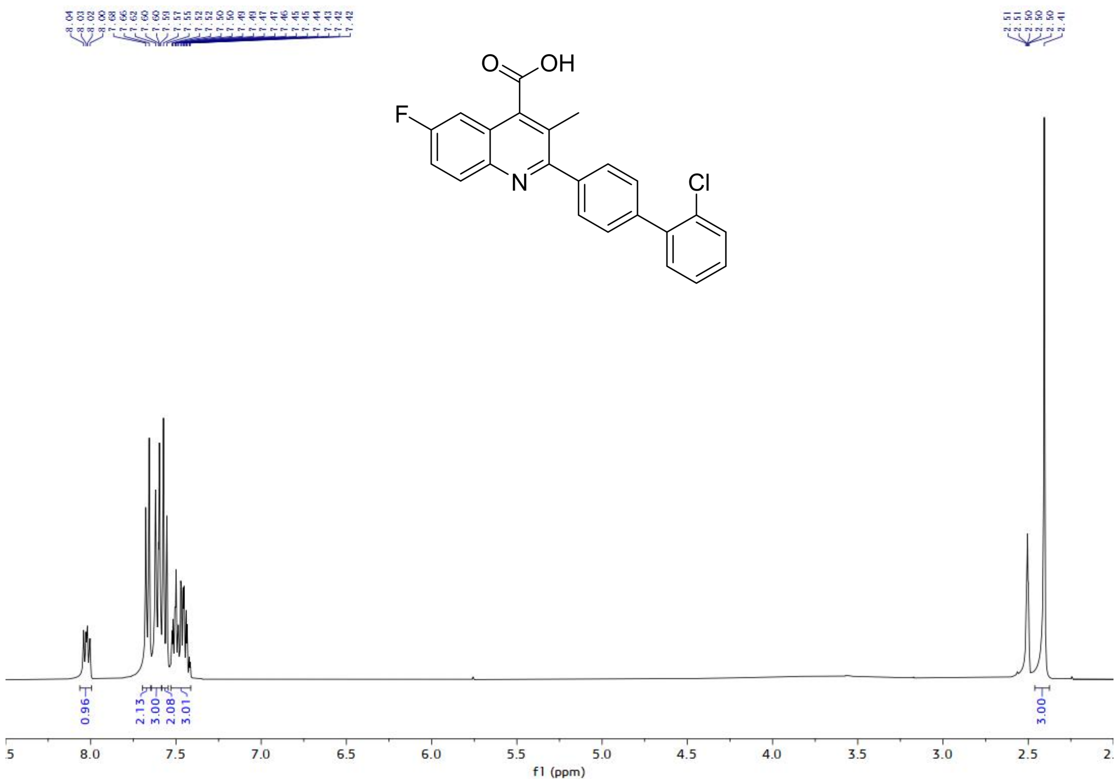

^{1}H-NMR spectrum of AA3

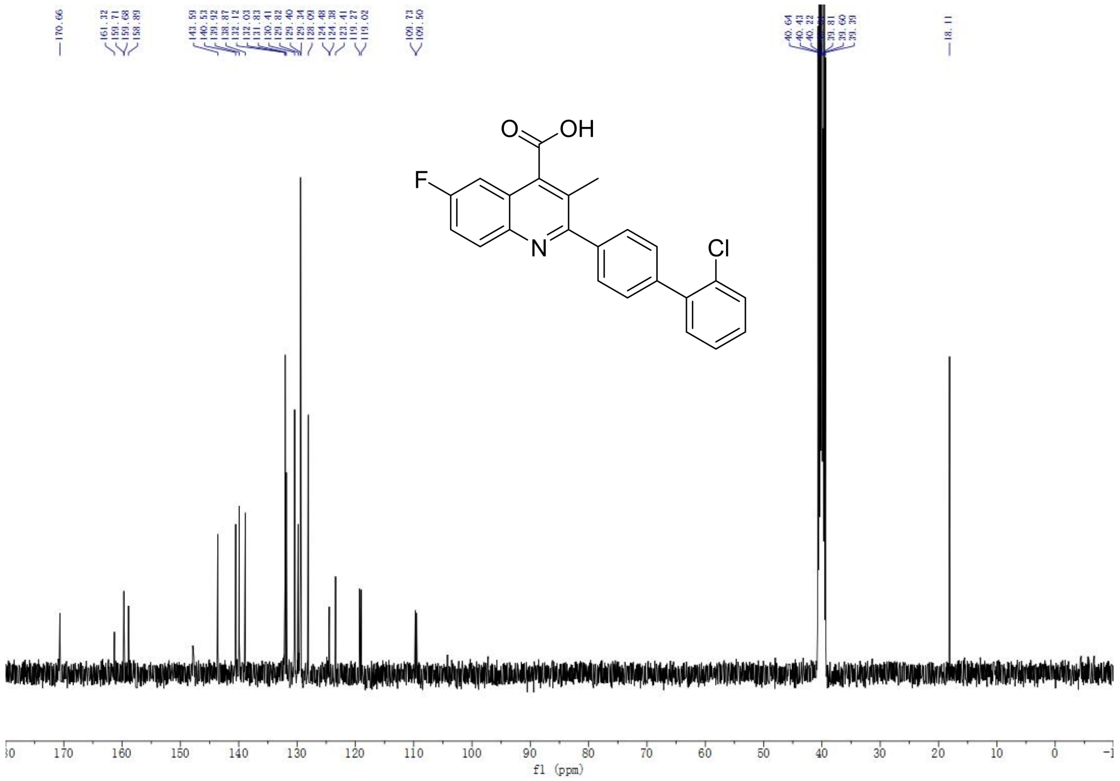

^{13}C-NMR spectrum of AA3

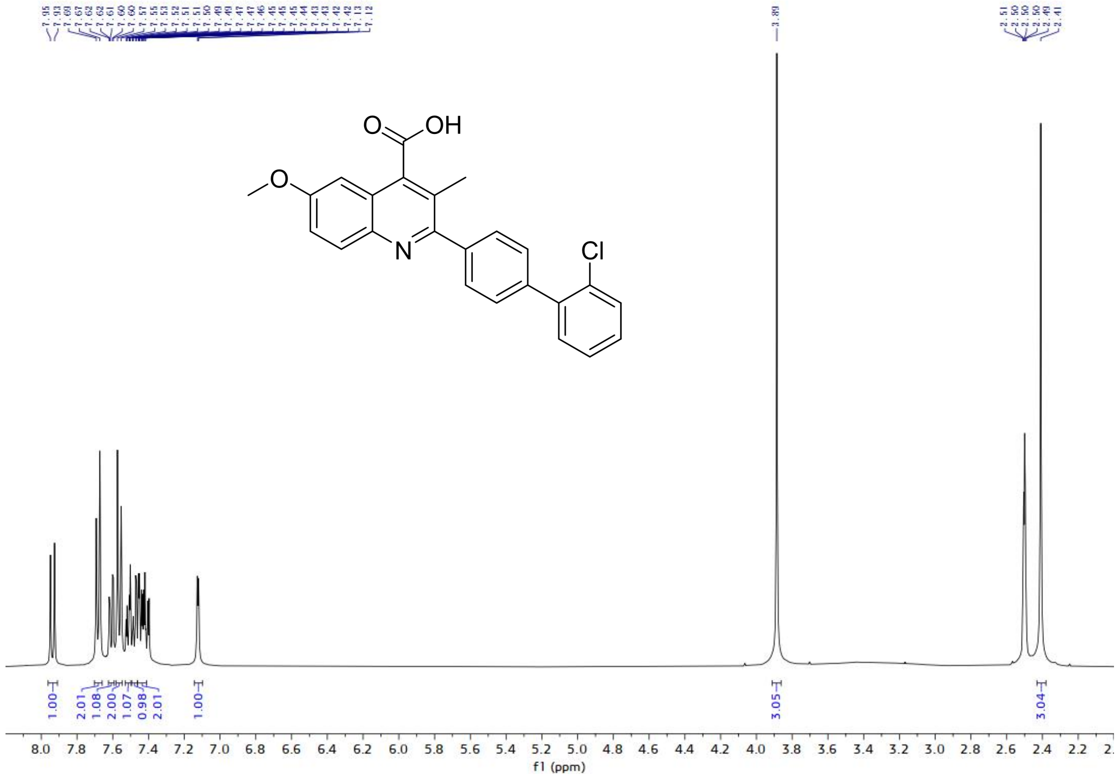

^{1}H-NMR spectrum of BA3

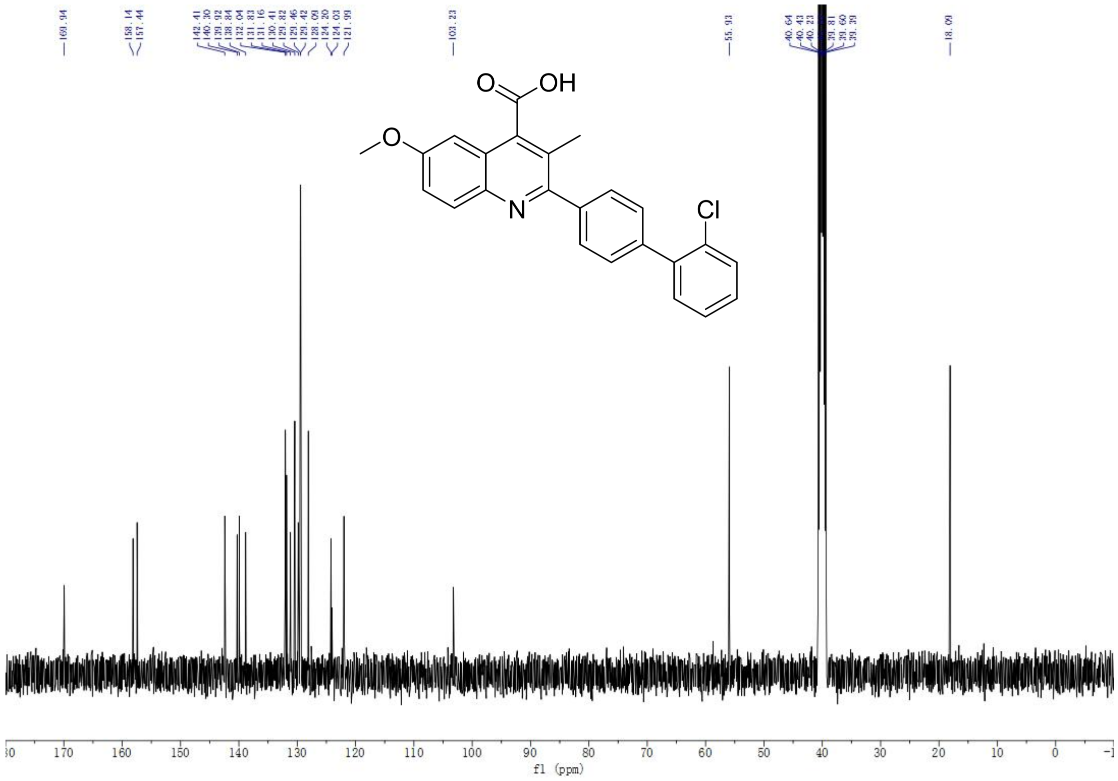

^{13}C-NMR spectrum of BA3

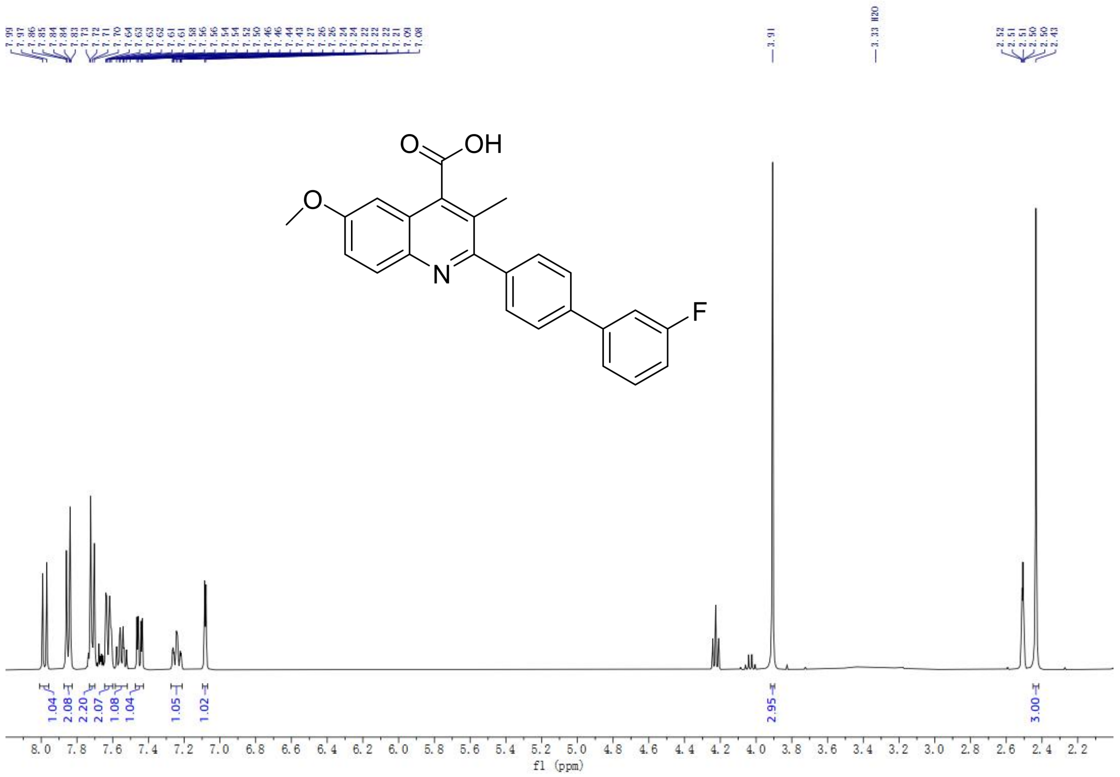

^{1}H-NMR spectrum of BA4

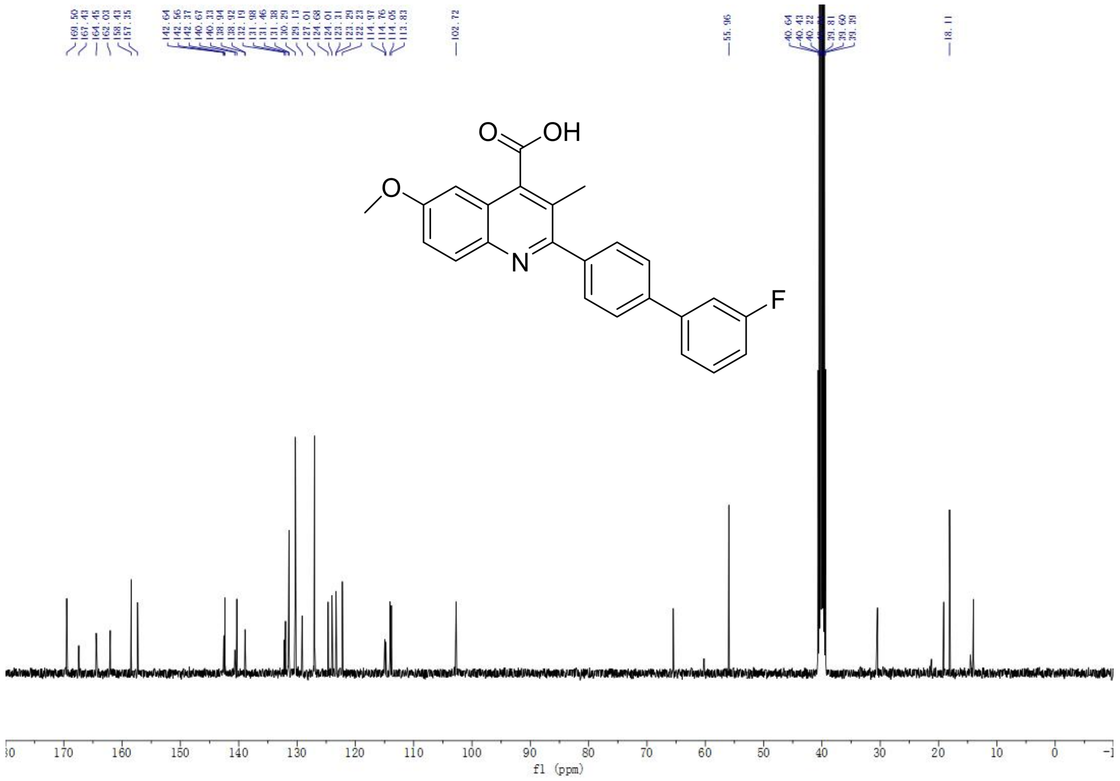

^{13}C-NMR spectrum of BA4

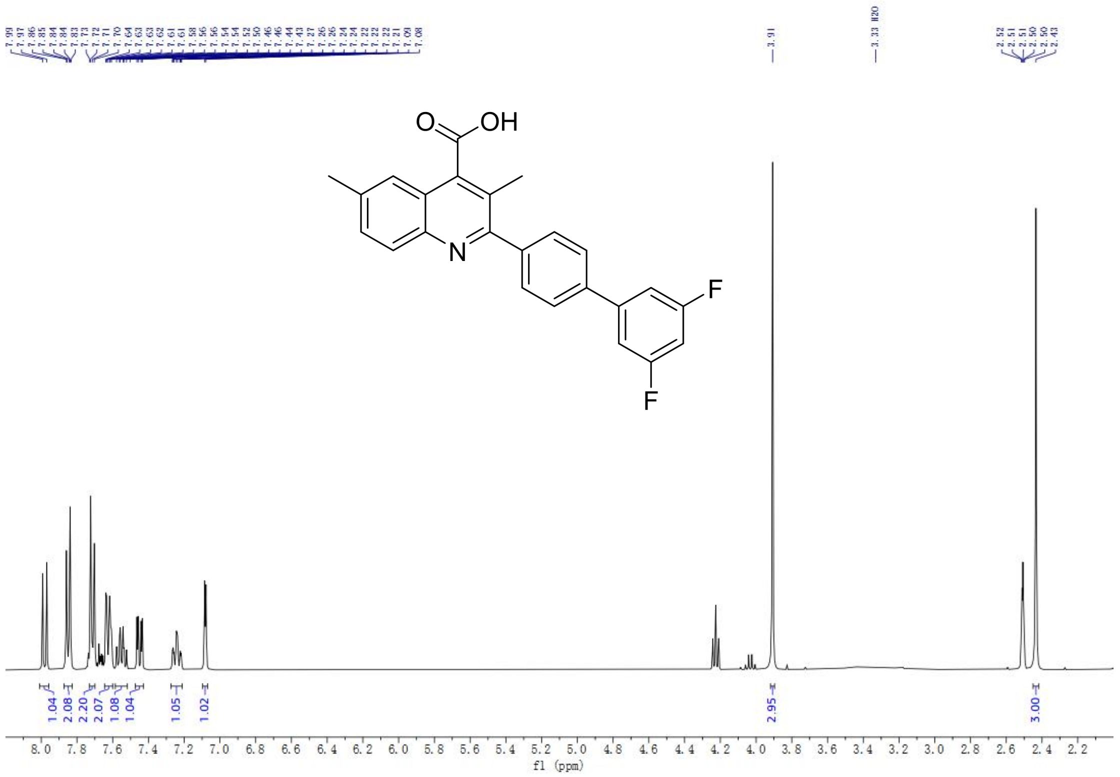

^{1}H-NMR spectrum of CA2

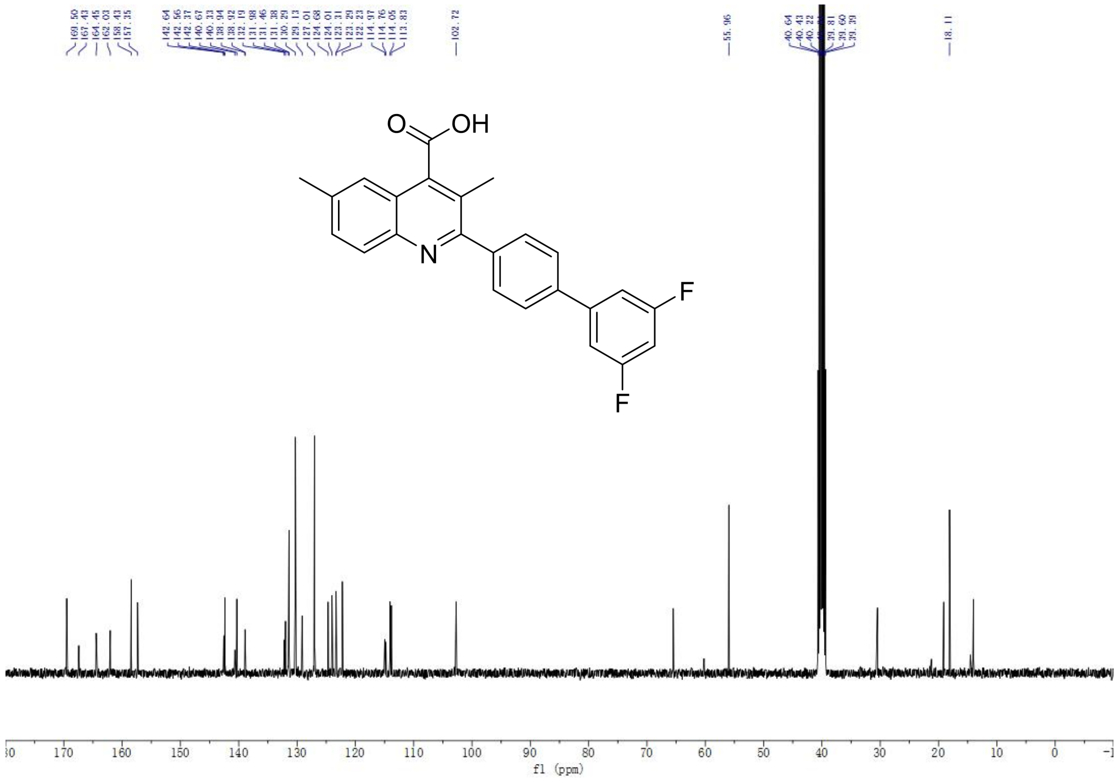

^{13}C-NMR spectrum of CA2

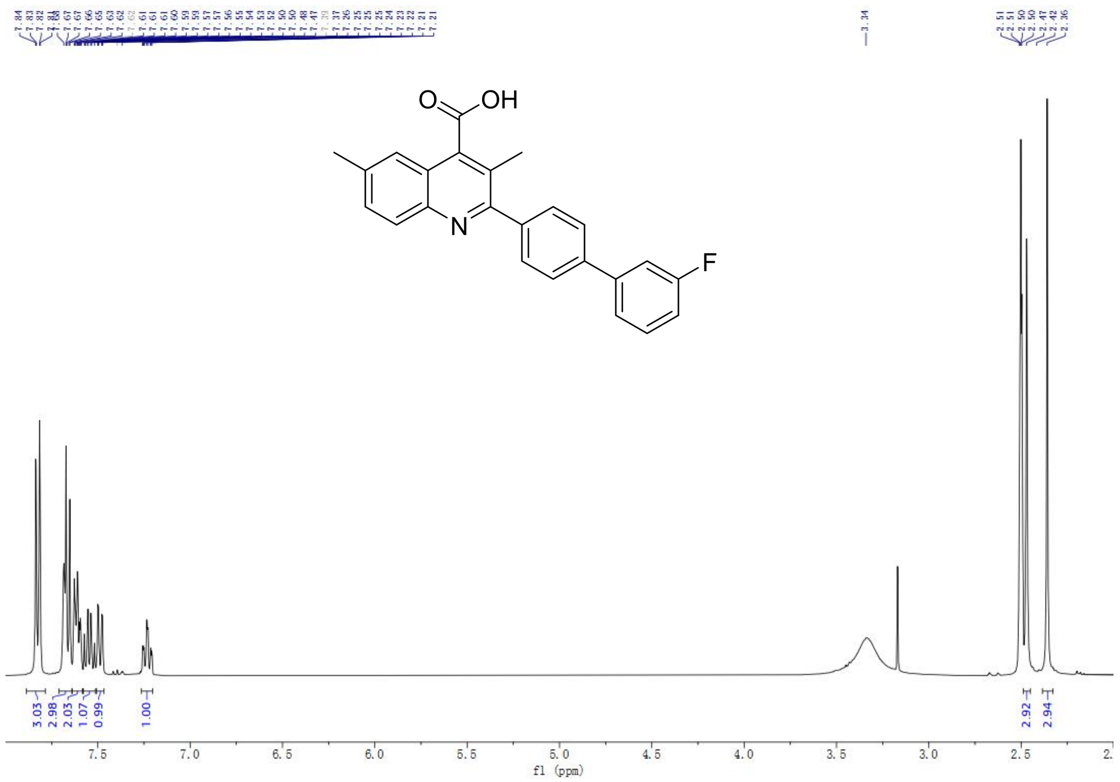

^{1}H-NMR spectrum of CA4

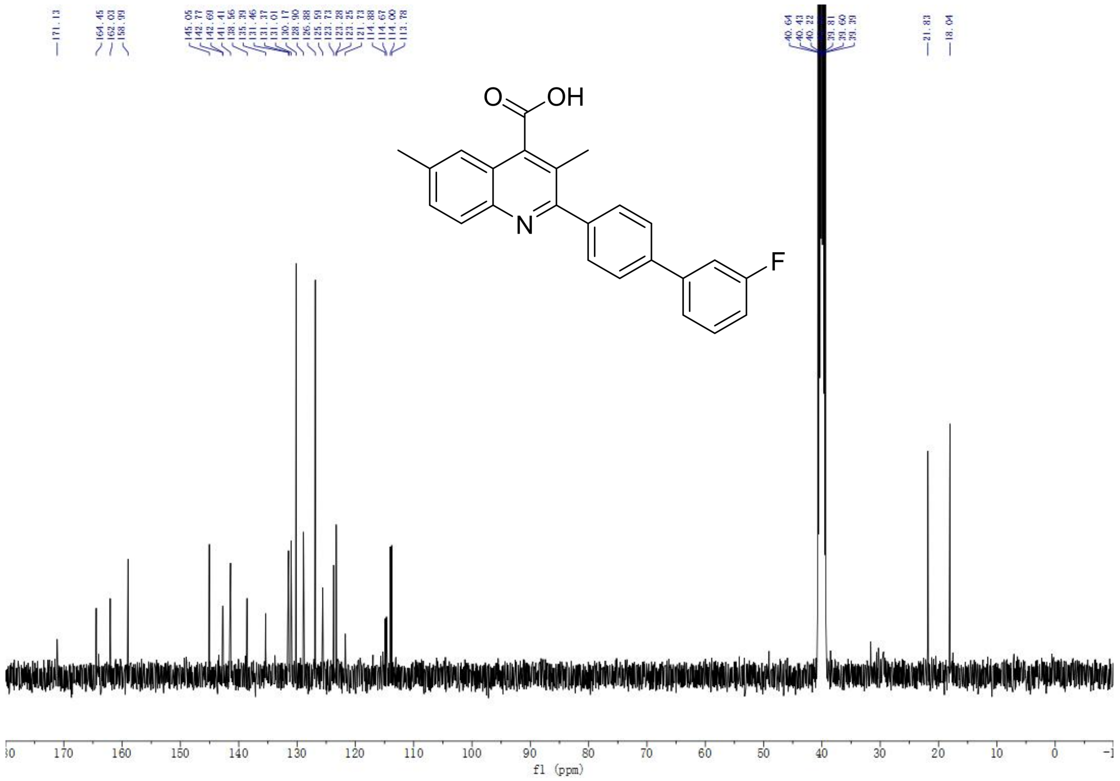

^{13}C-NMR spectrum of CA4

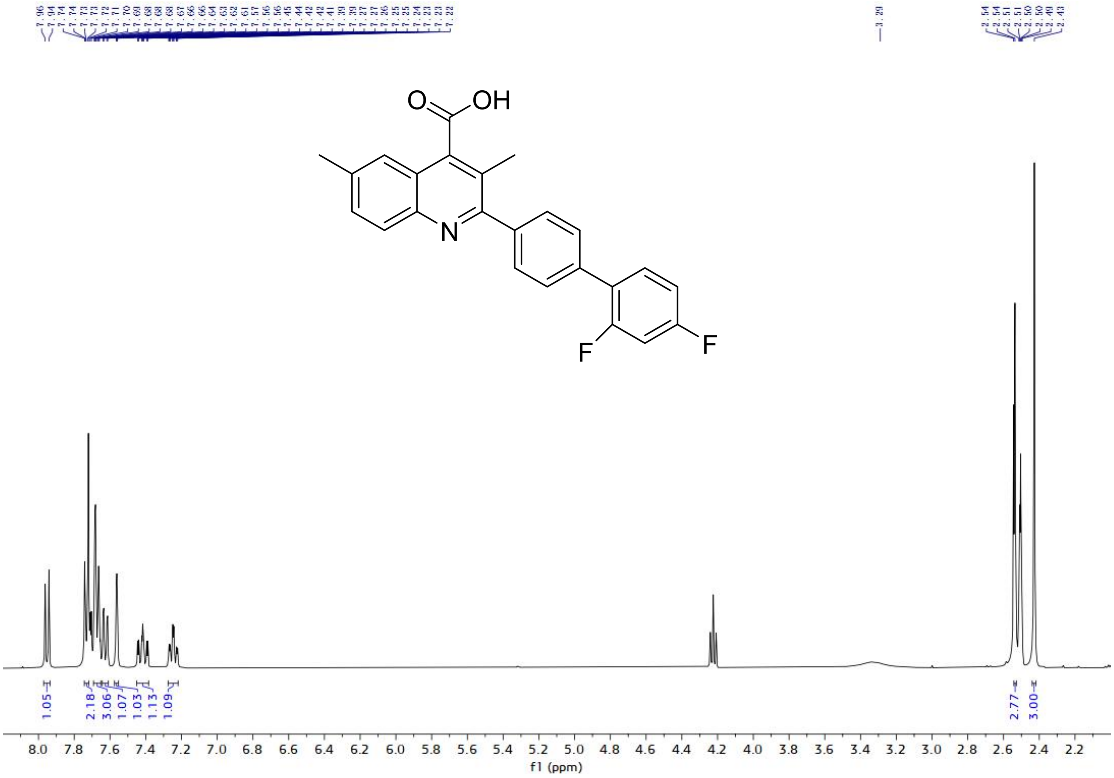

^{1}H-NMR spectrum of CA8

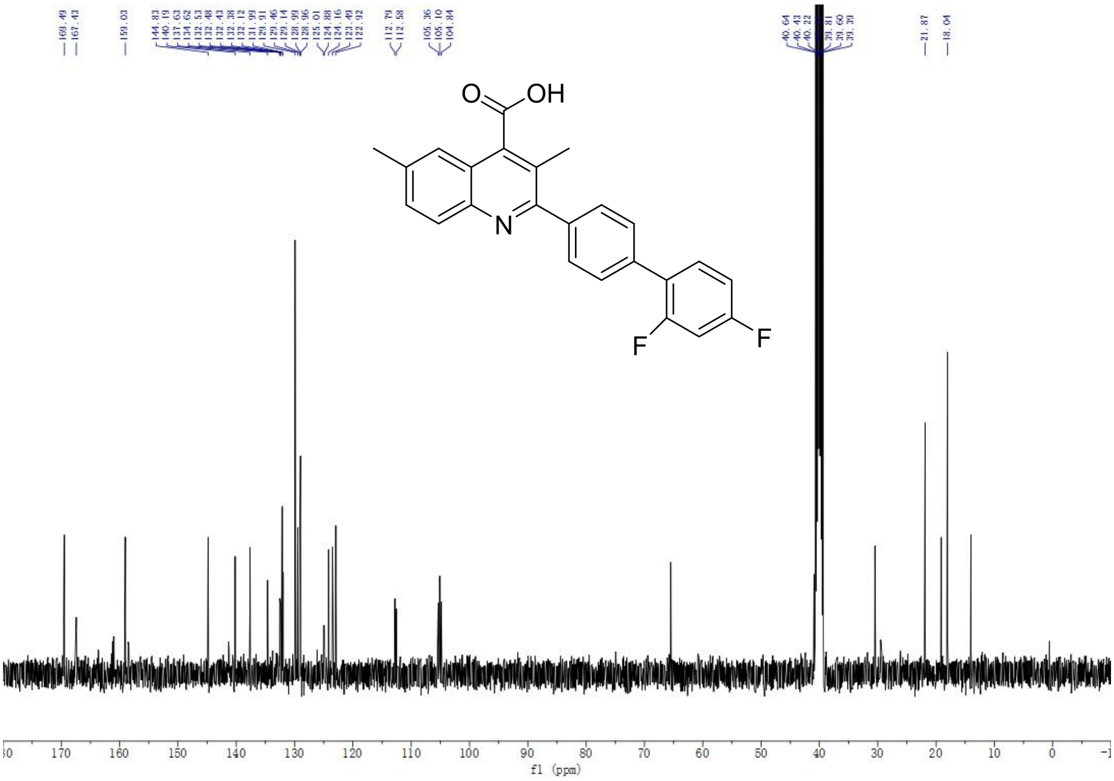

^{13}C-NMR spectrum of CA8

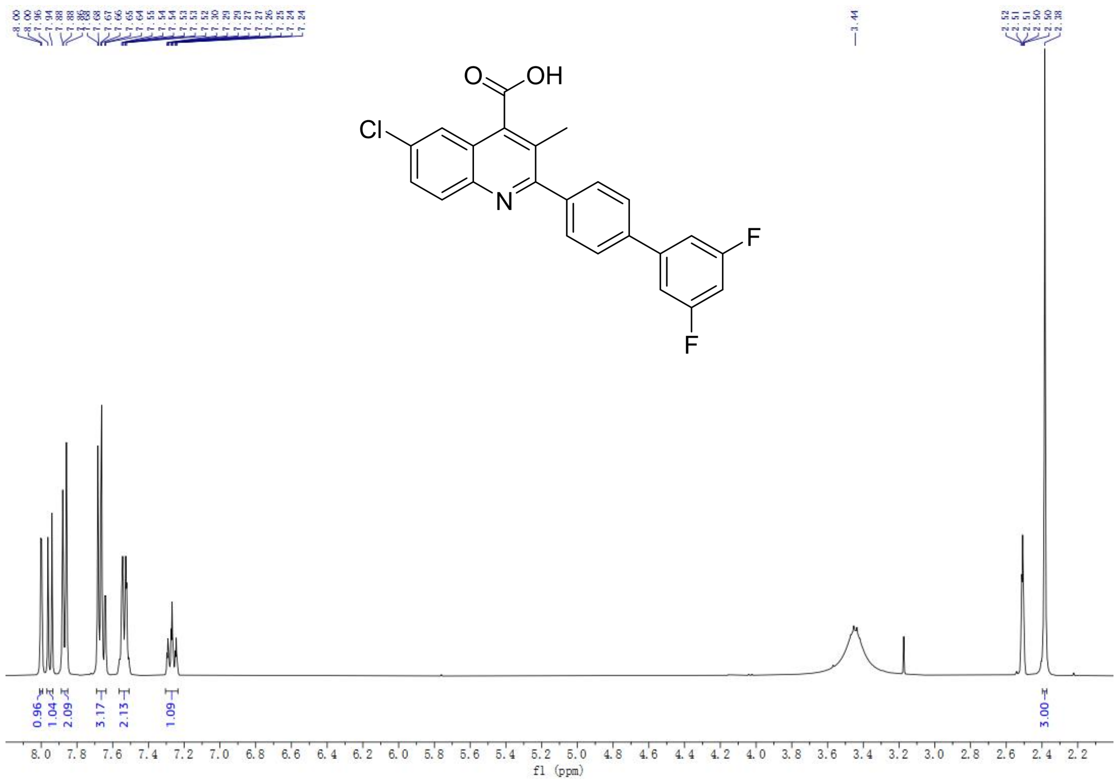

^{1}H-NMR spectrum of EA2

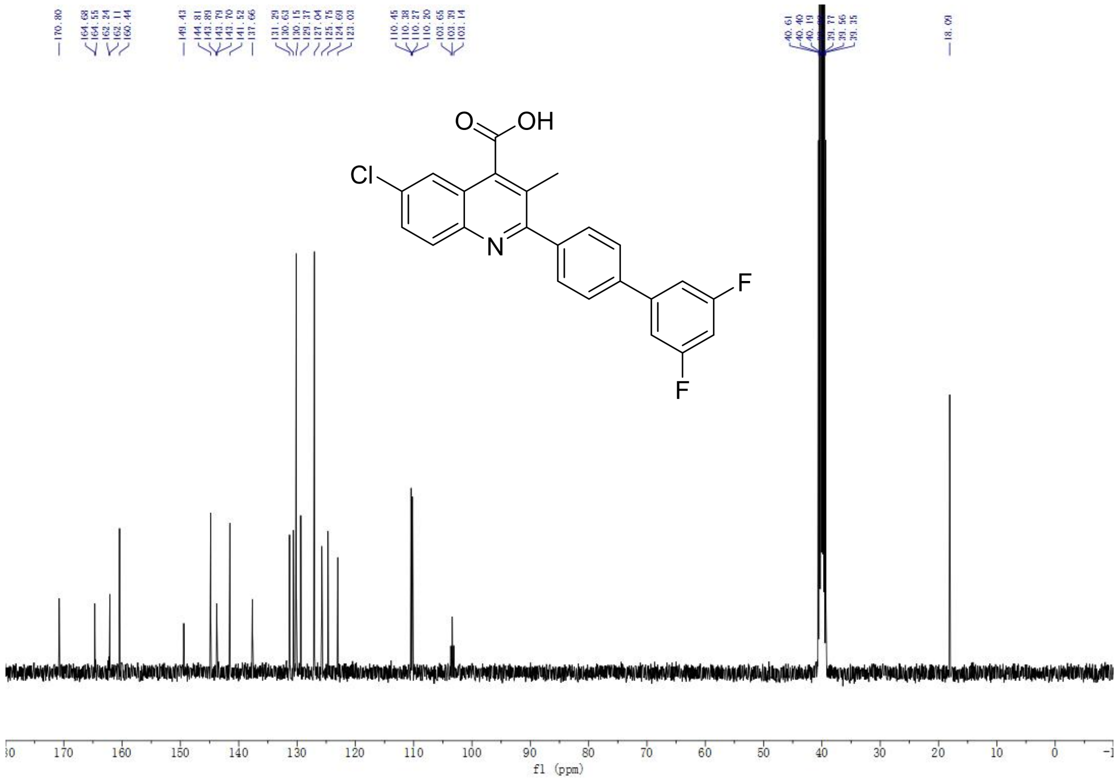

^{13}C-NMR spectrum of EA2

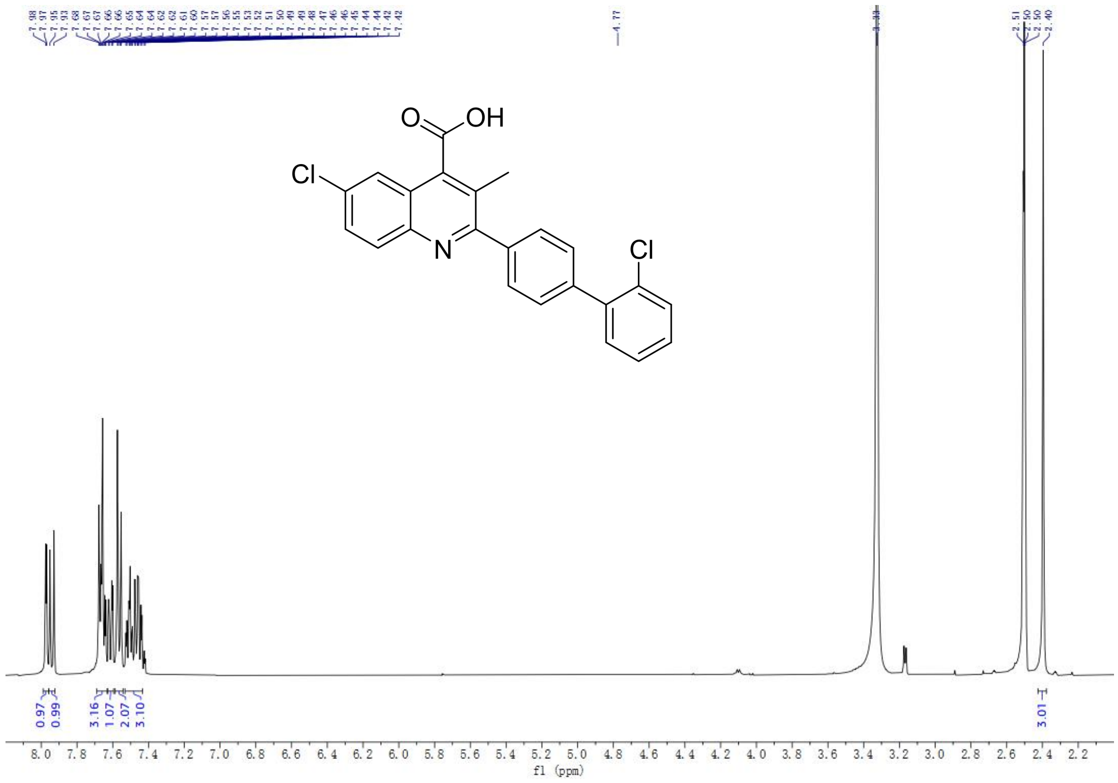

^{1}H-NMR spectrum of EA3

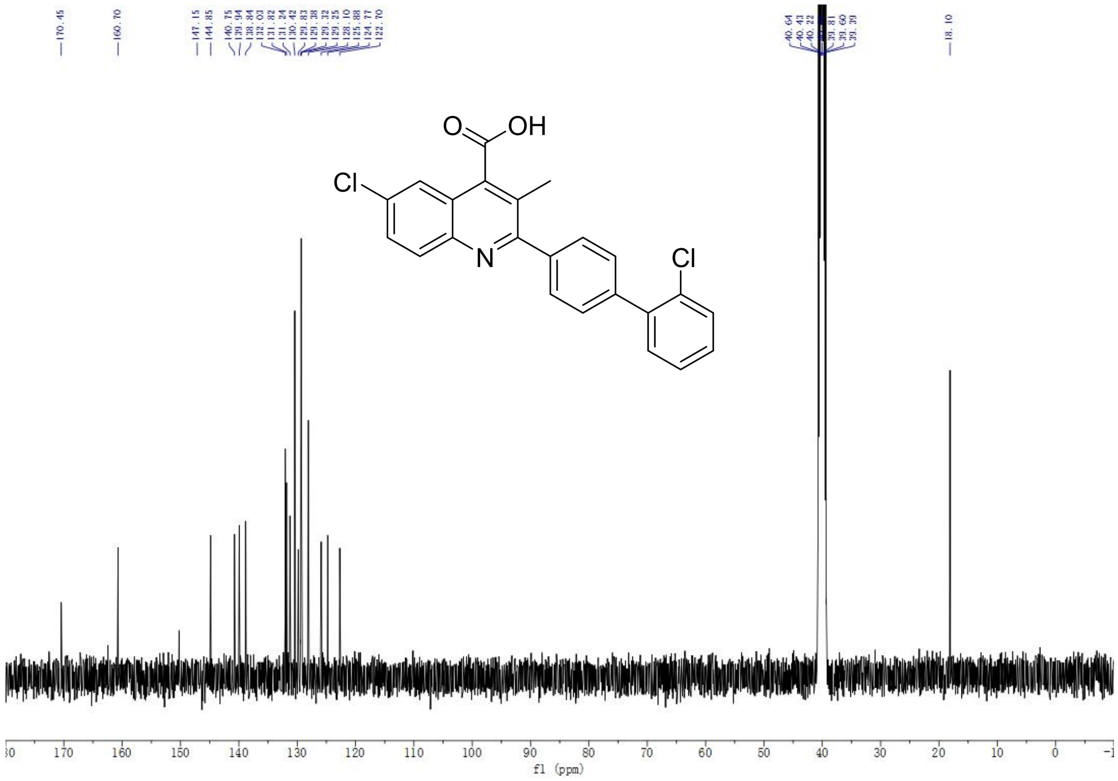

^{13}C-NMR spectrum of EA3

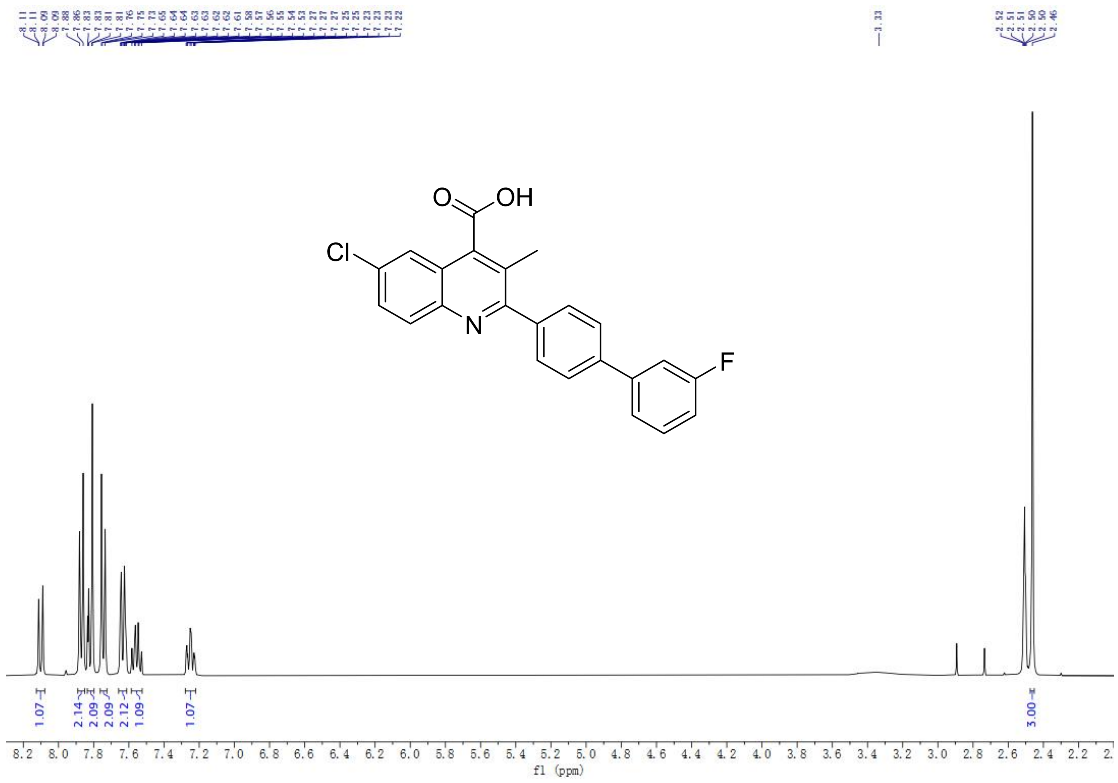

^{1}H-NMR spectrum of EA4

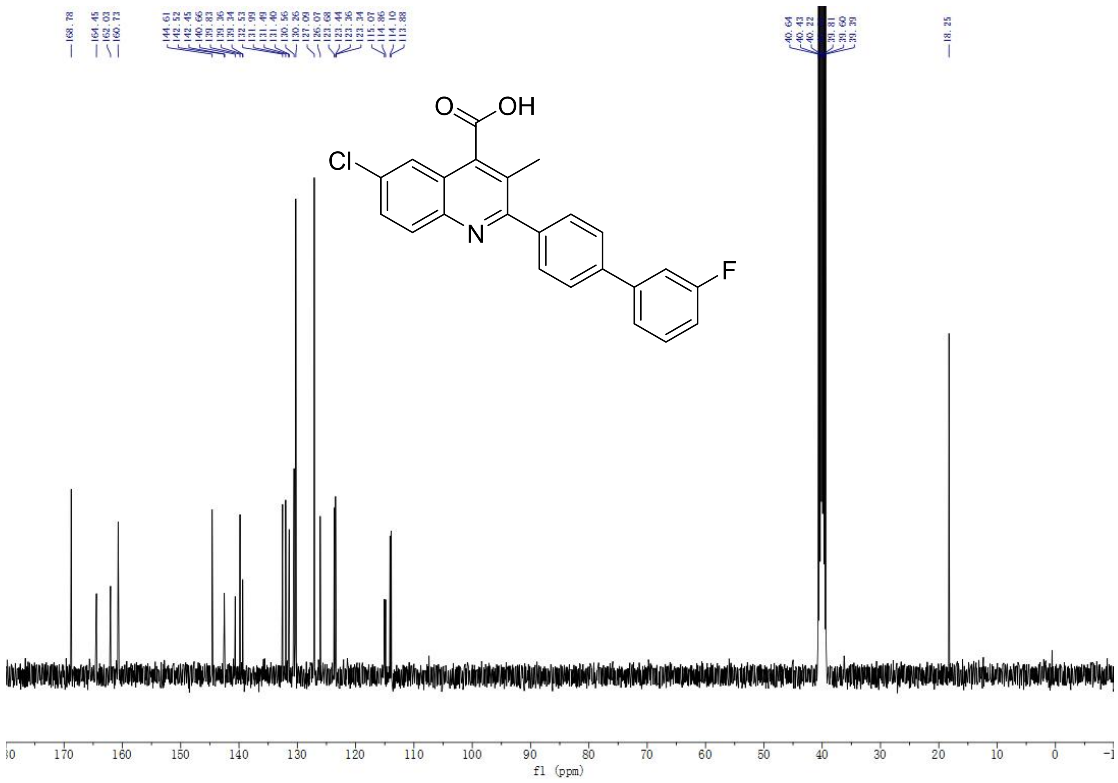

^{13}C-NMR spectrum of EA4

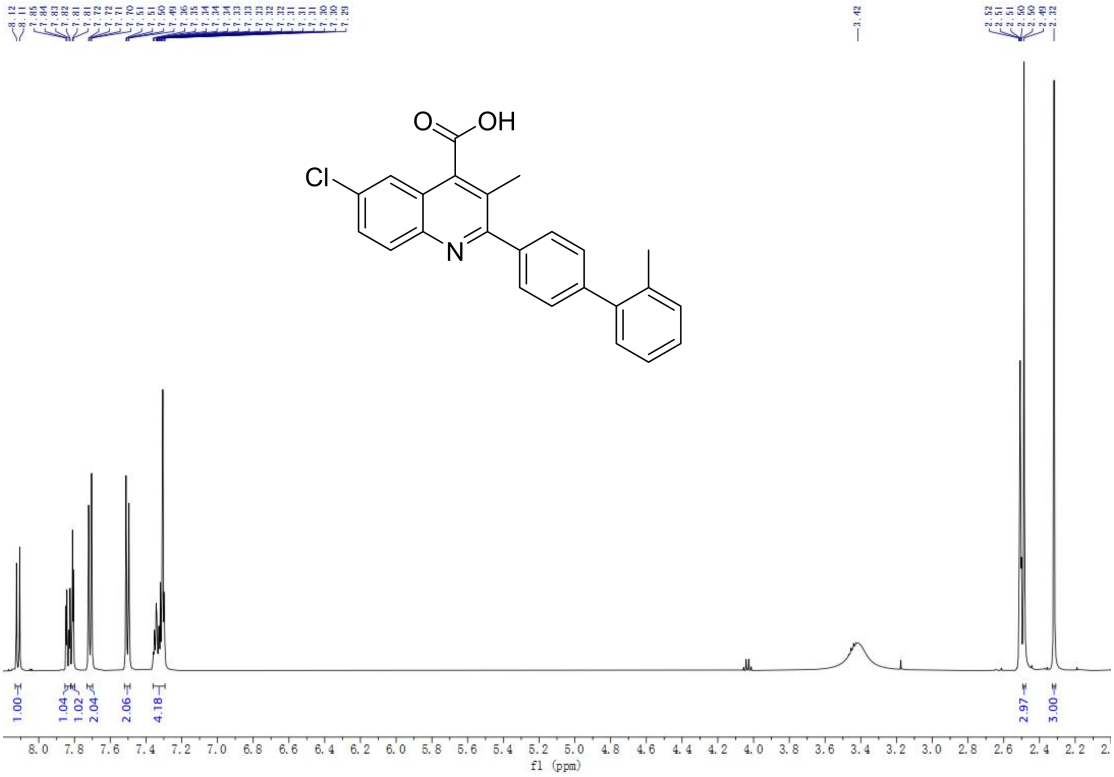

^{1}H-NMR spectrum of EA6

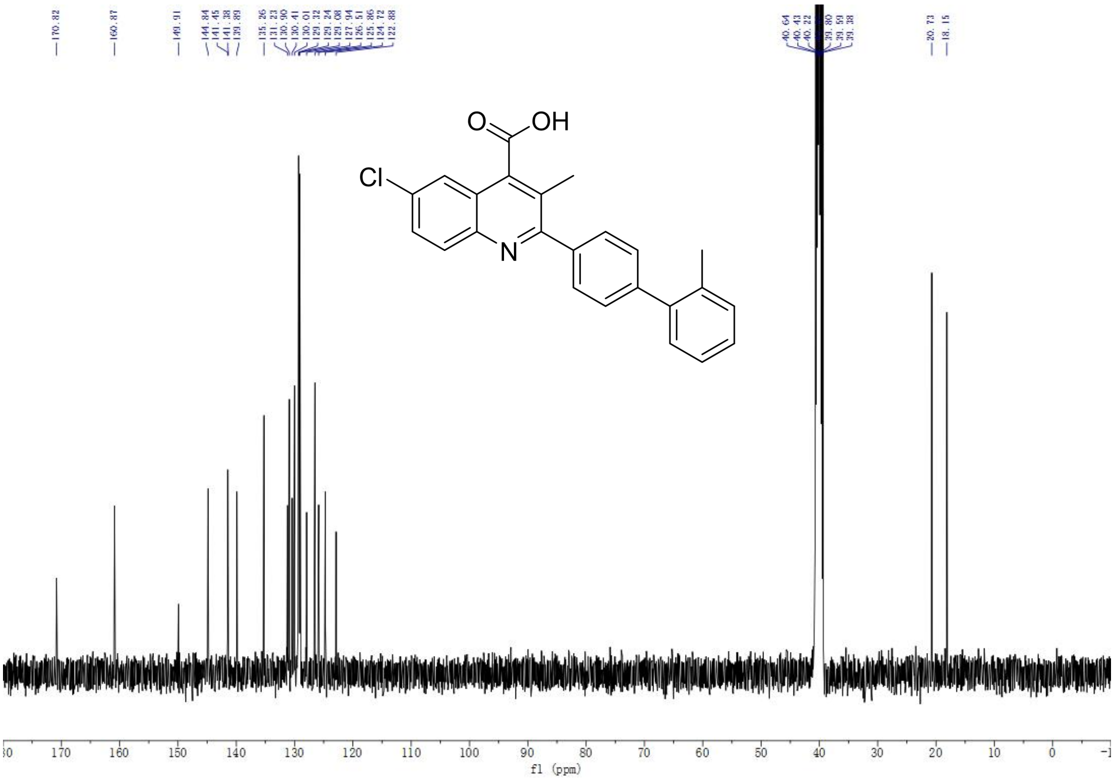

^{13}C-NMR spectrum of EA6

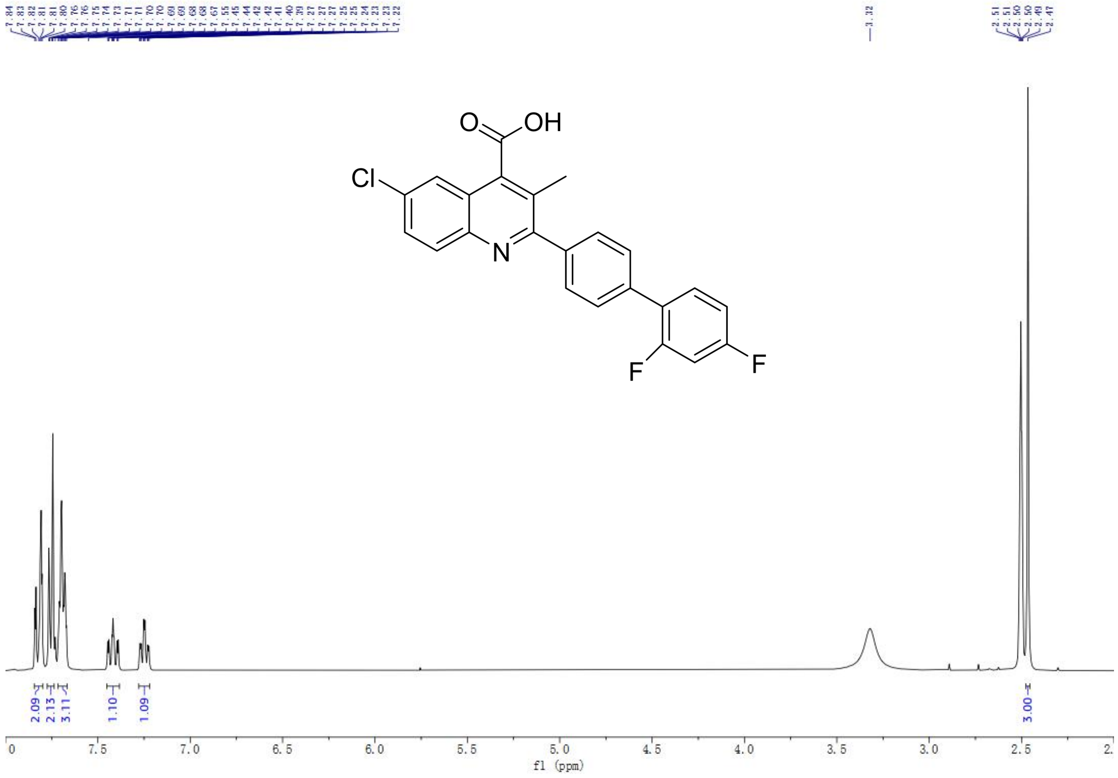

^{1}H-NMR spectrum of EA8

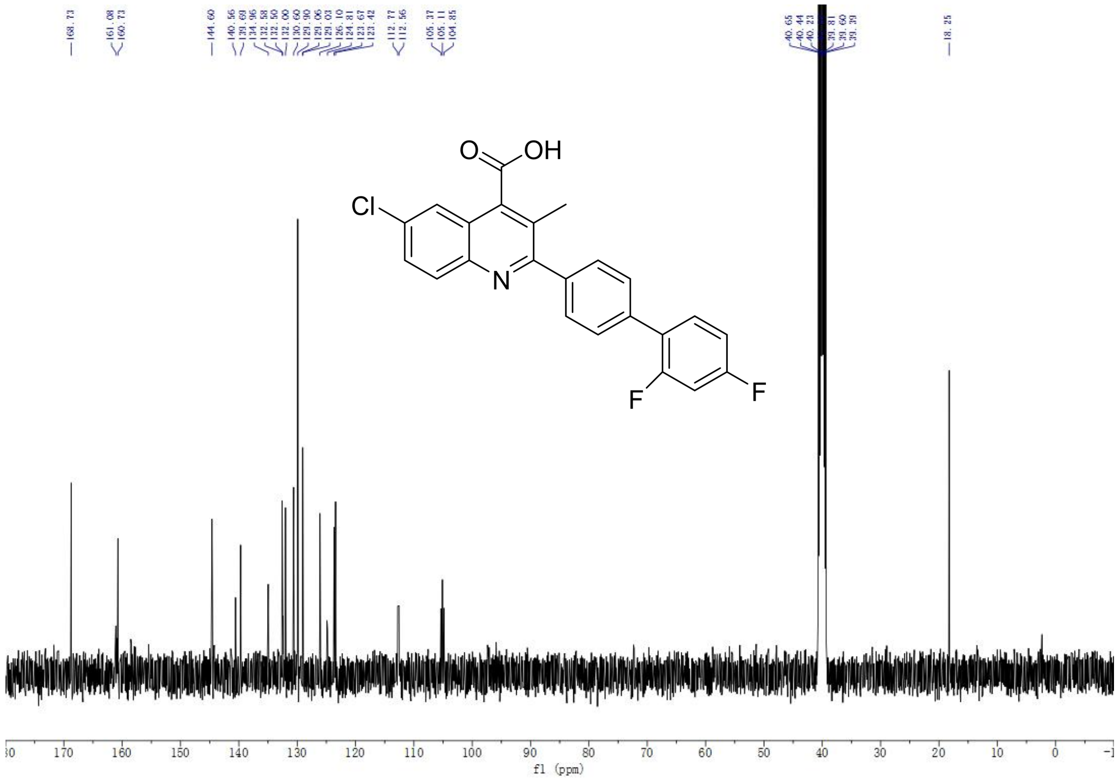

^{13}C-NMR spectrum of EA8

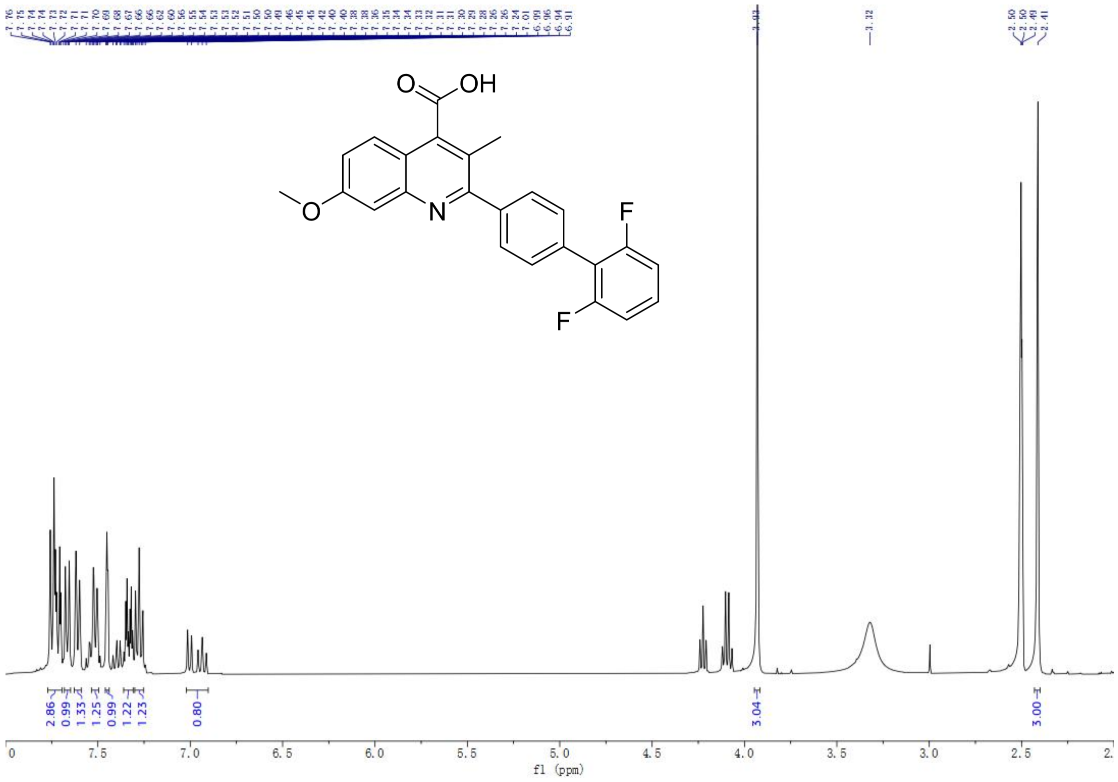

^{1}H-NMR spectrum of FA11

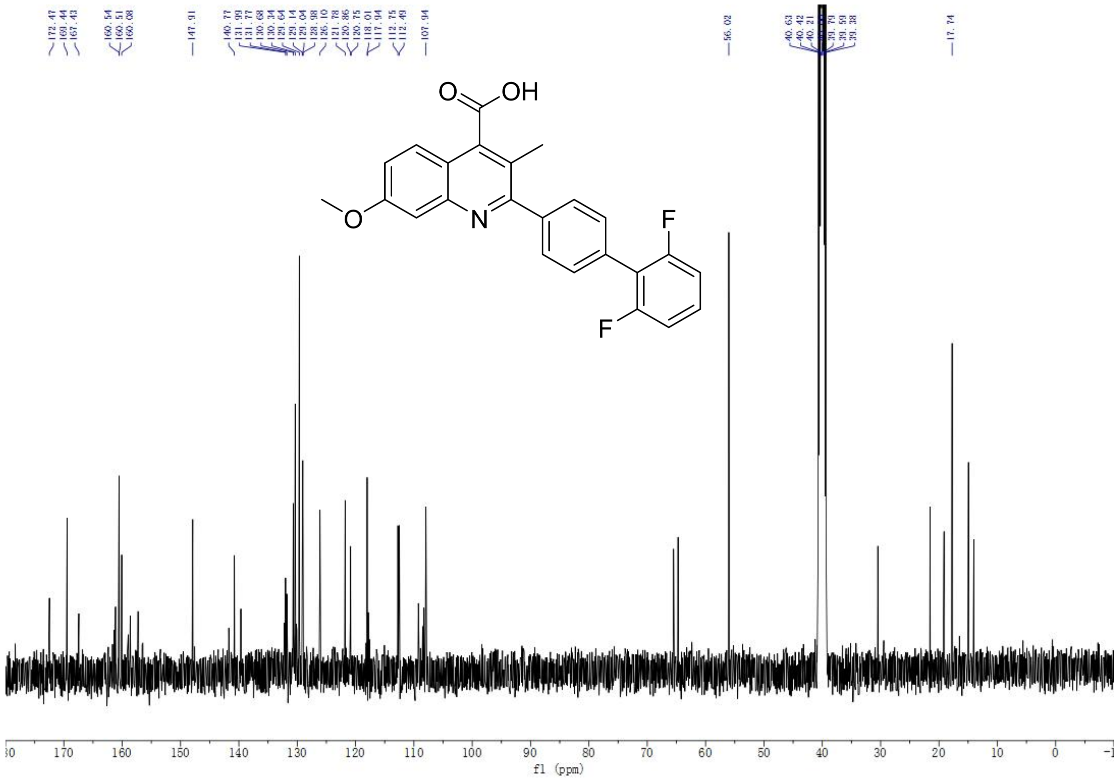

^{13}C-NMR spectrum of FA11

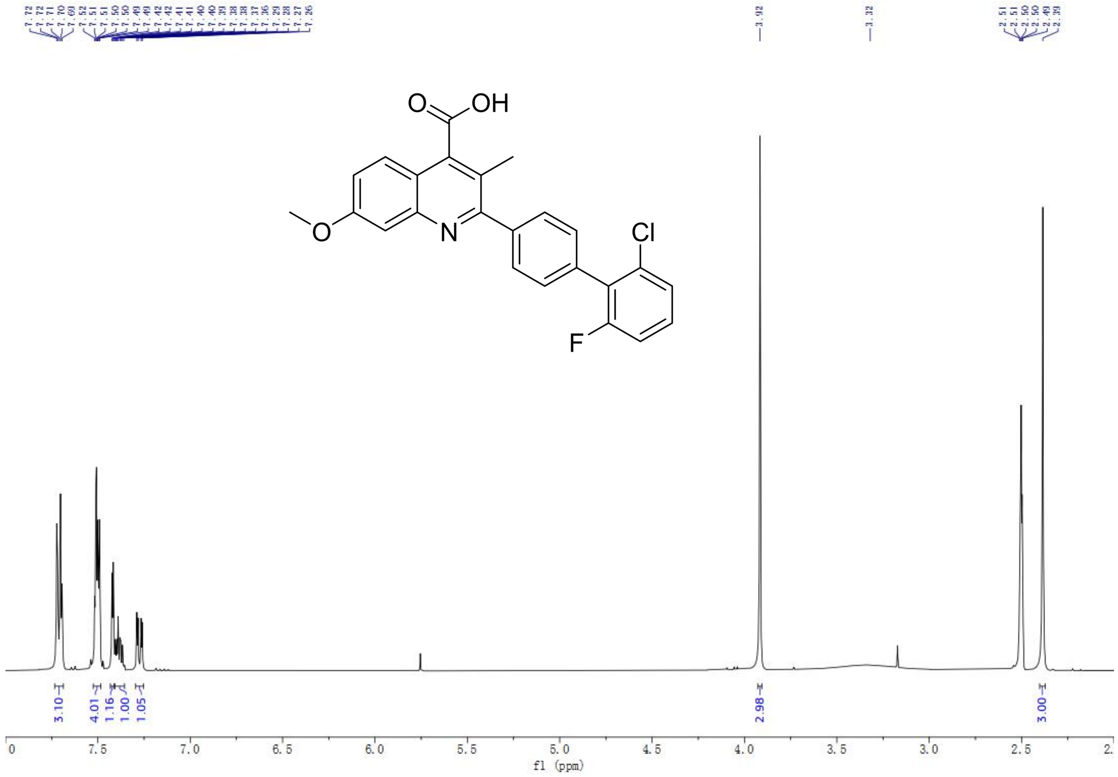

^{1}H-NMR spectrum of FA12

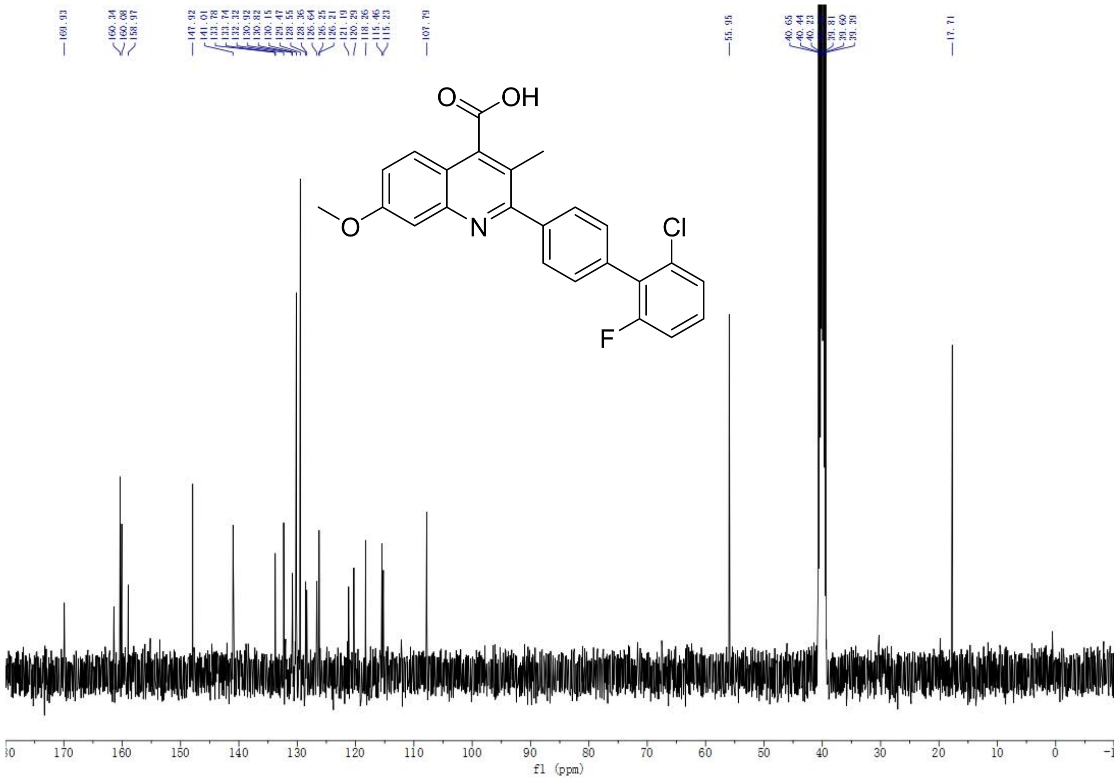

^{13}C-NMR spectrum of FA12

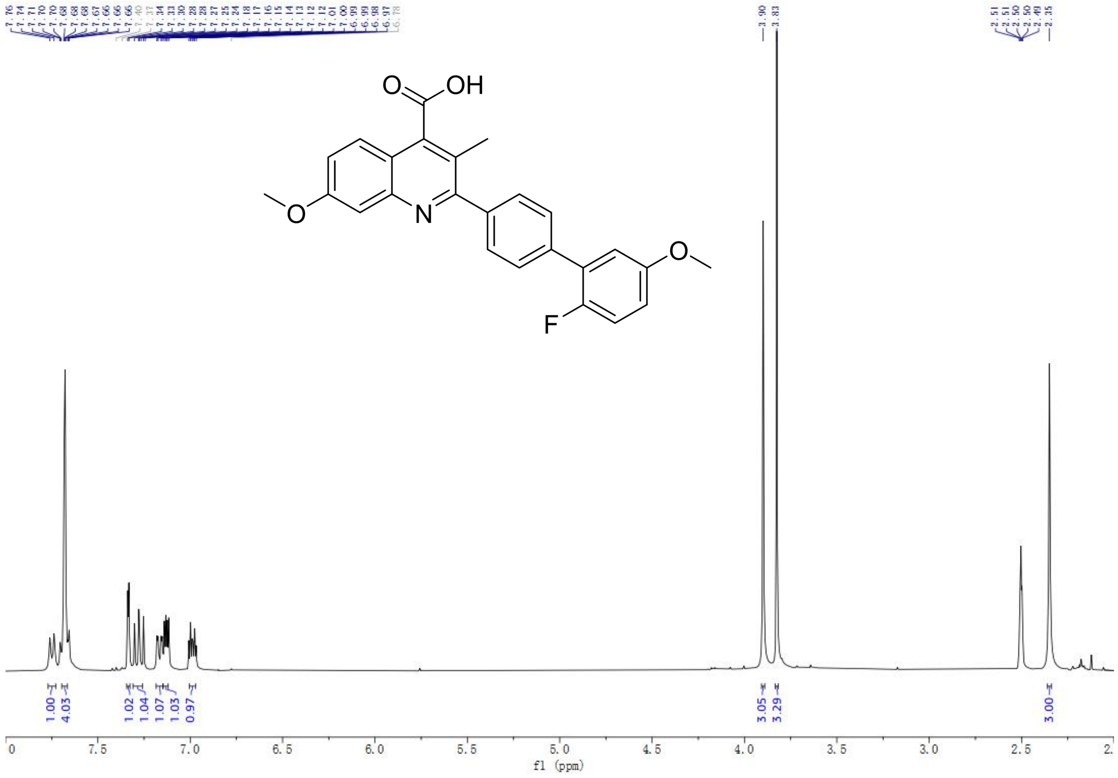

^{1}H-NMR spectrum of FA14

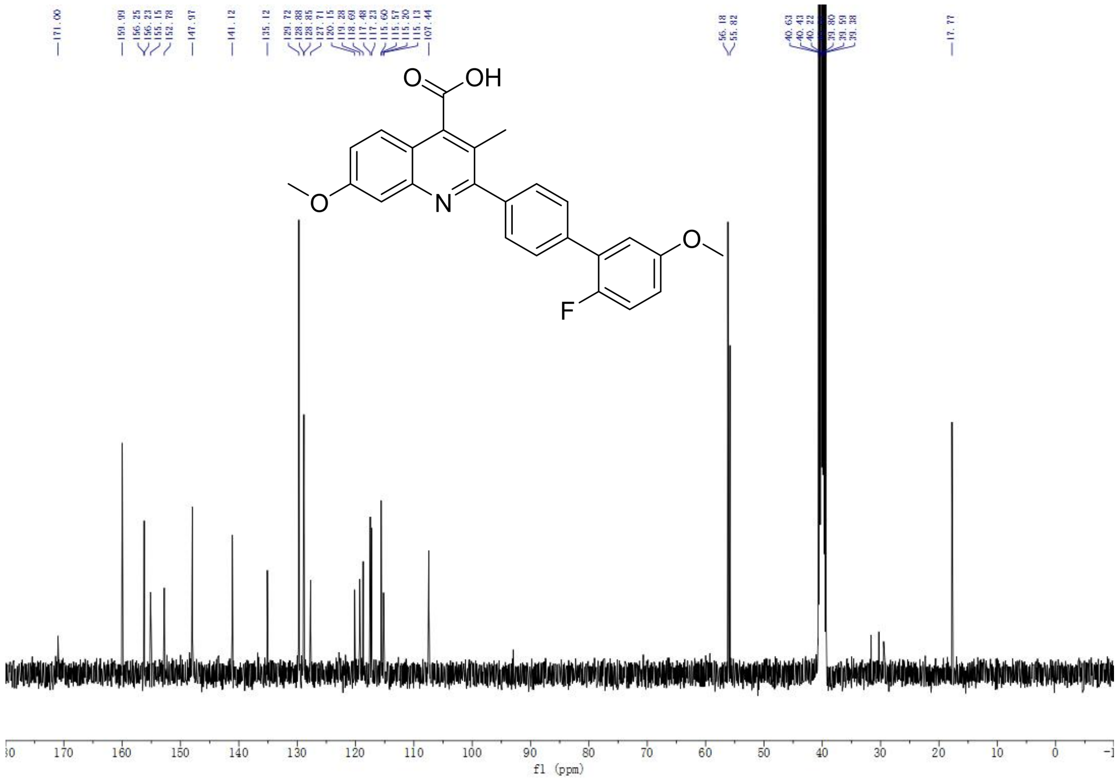

^{13}C-NMR spectrum of FA14

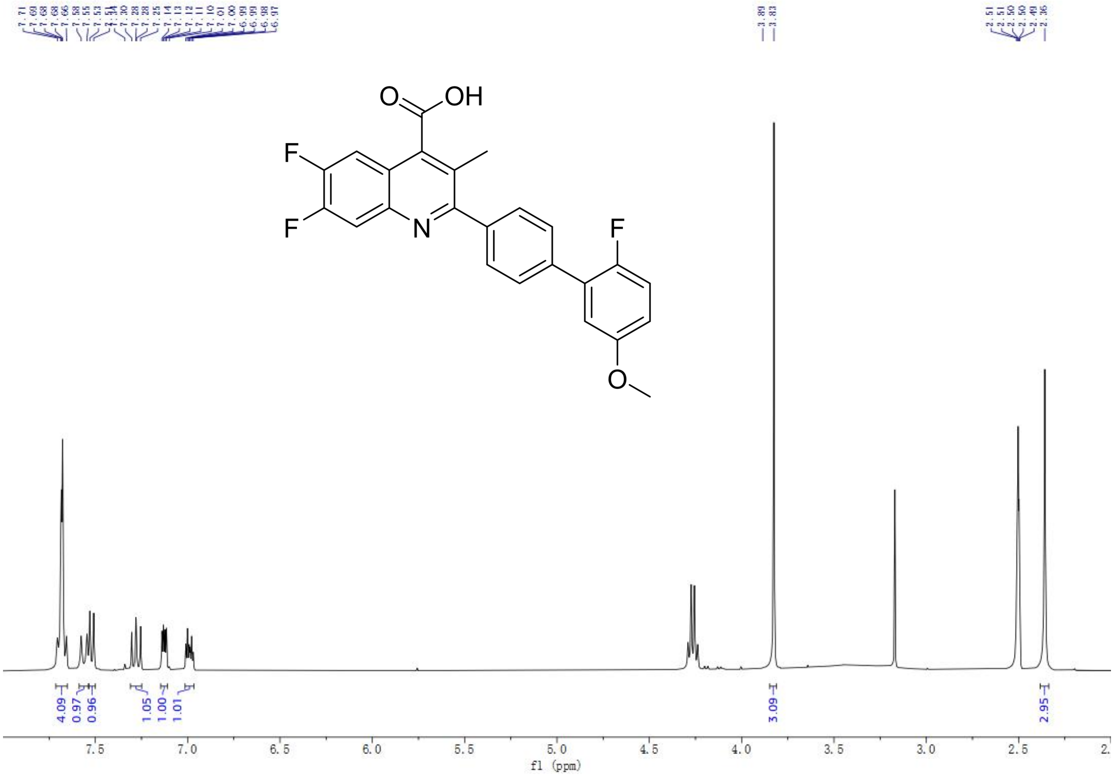

^{1}H-NMR spectrum of GA14

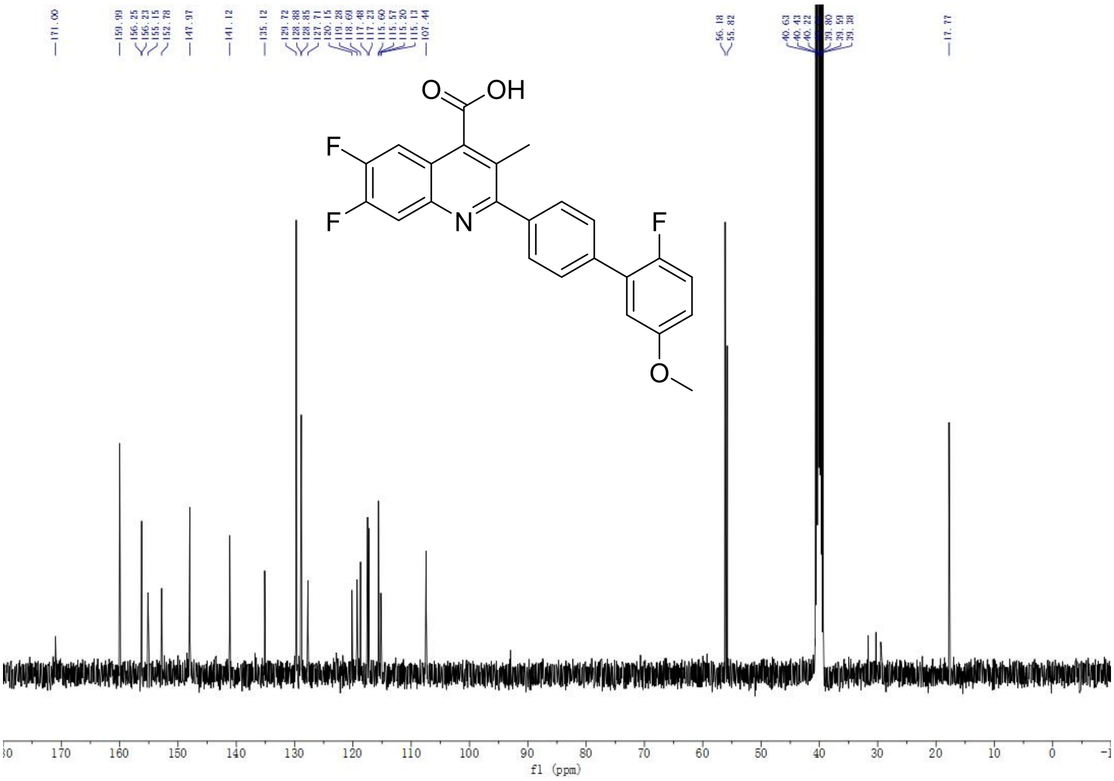

^{13}C-NMR spectrum of GA14

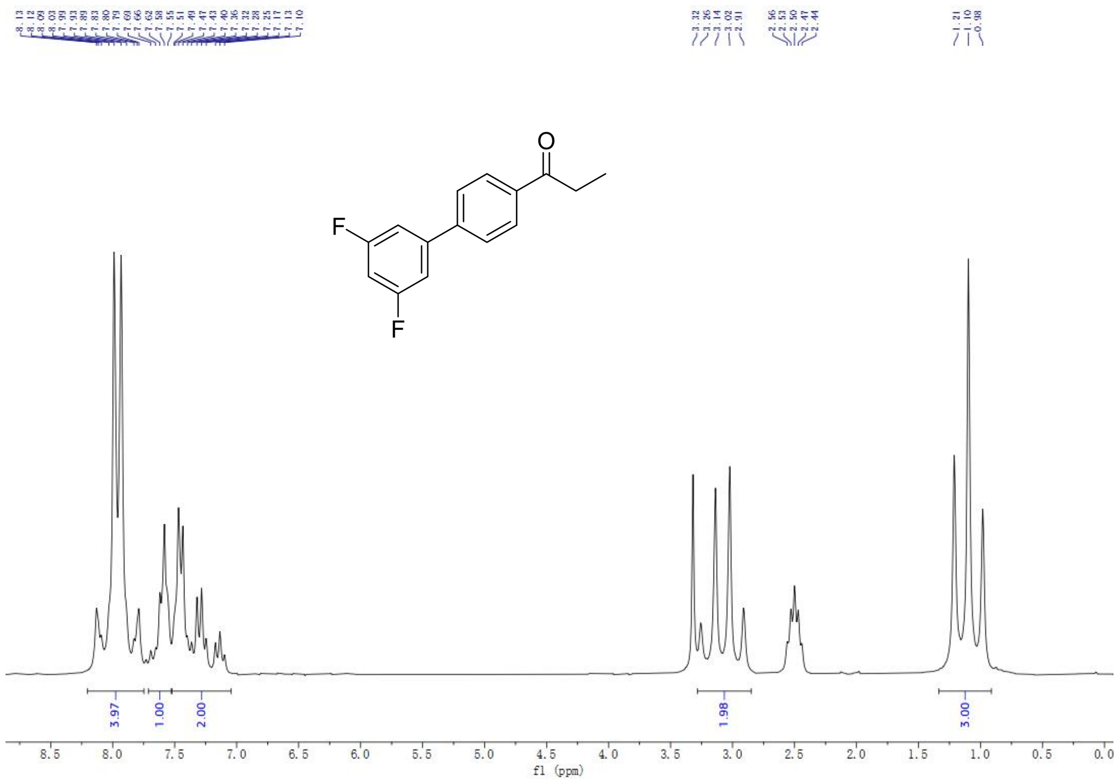

^{1}H-NMR spectrum of A2

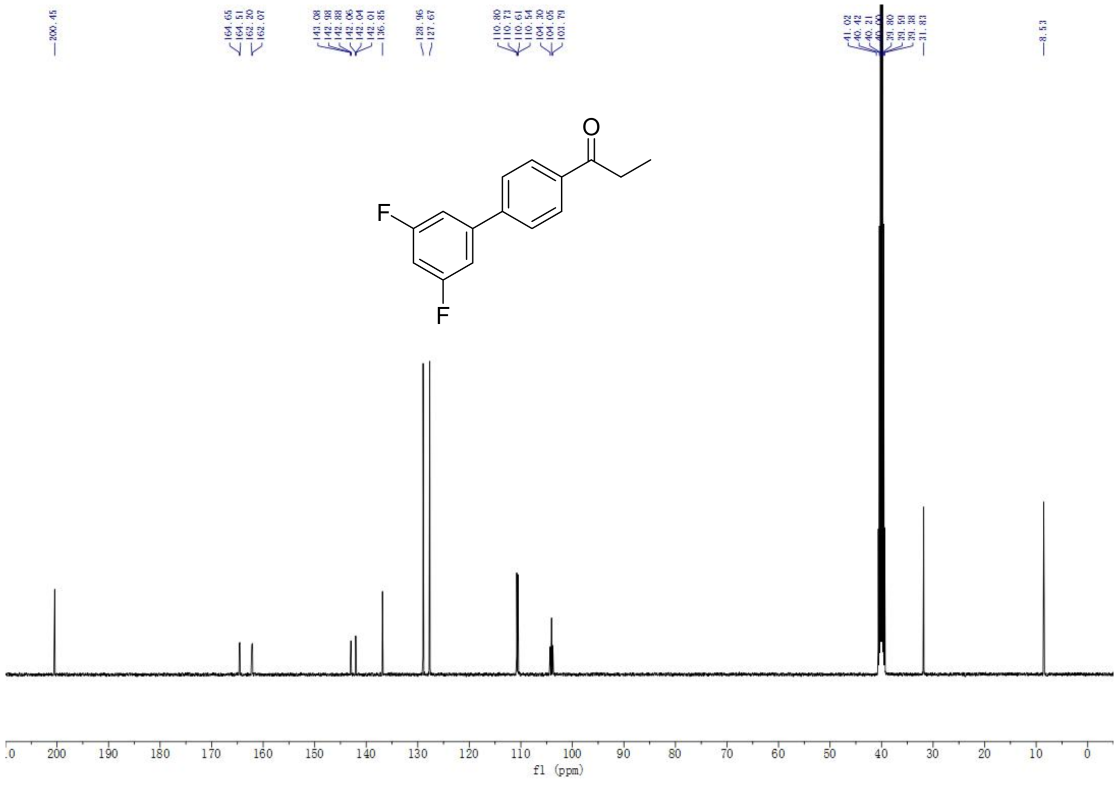

^{13}C-NMR spectrum of A2

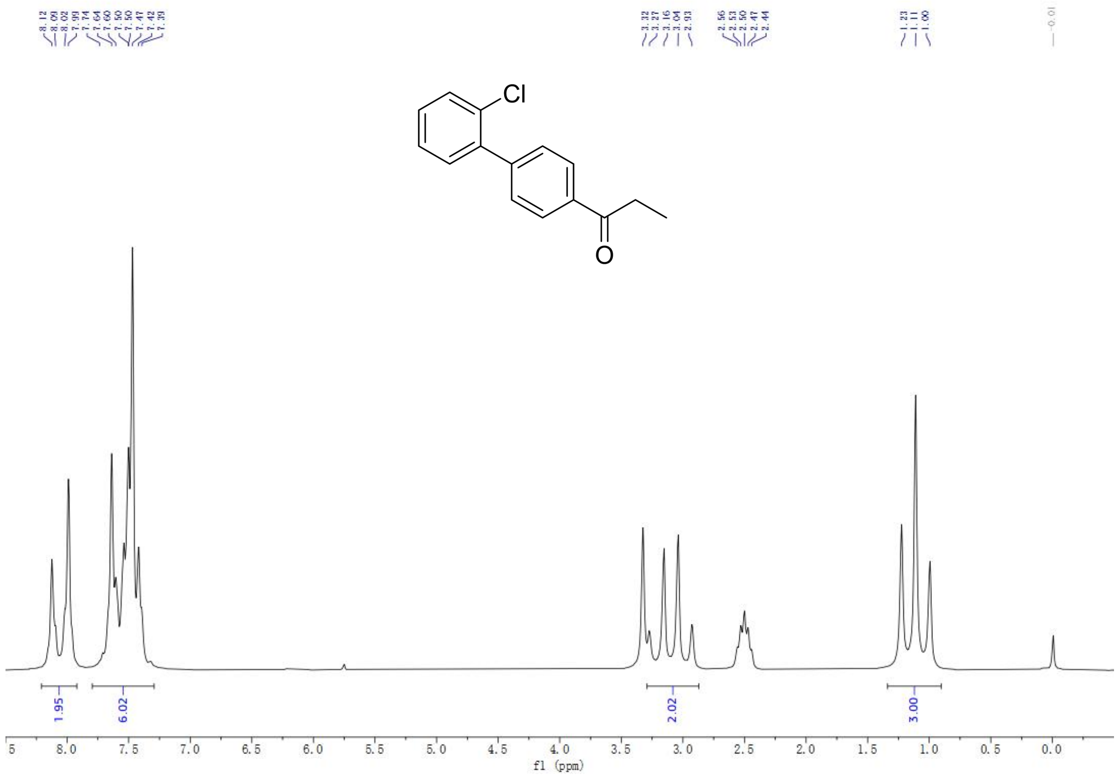

^{1}H-NMR spectrum of A3

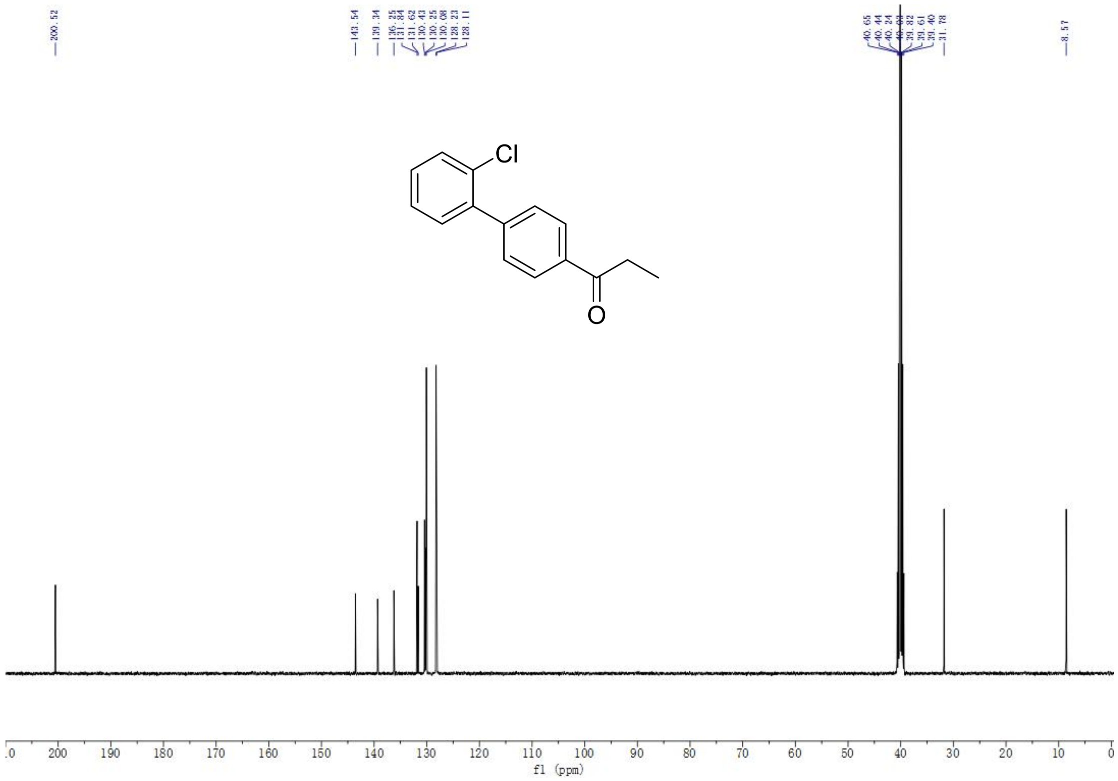

^{13}C-NMR spectrum of A3

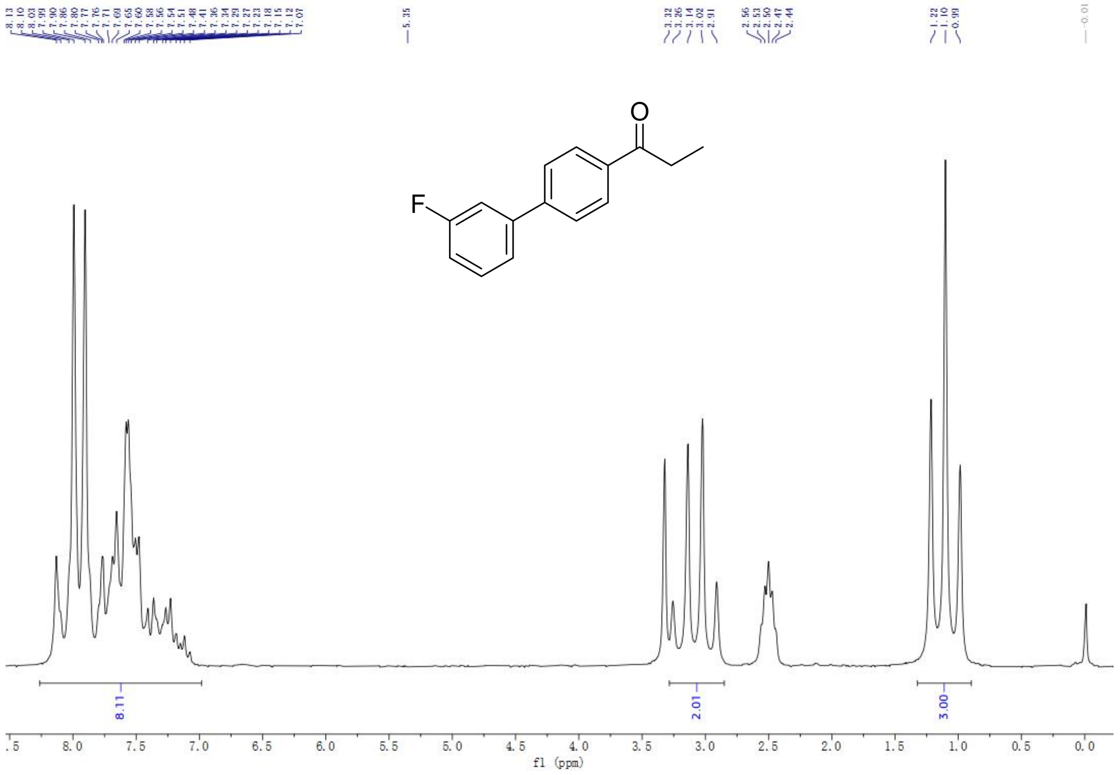

^{1}H-NMR spectrum of A4

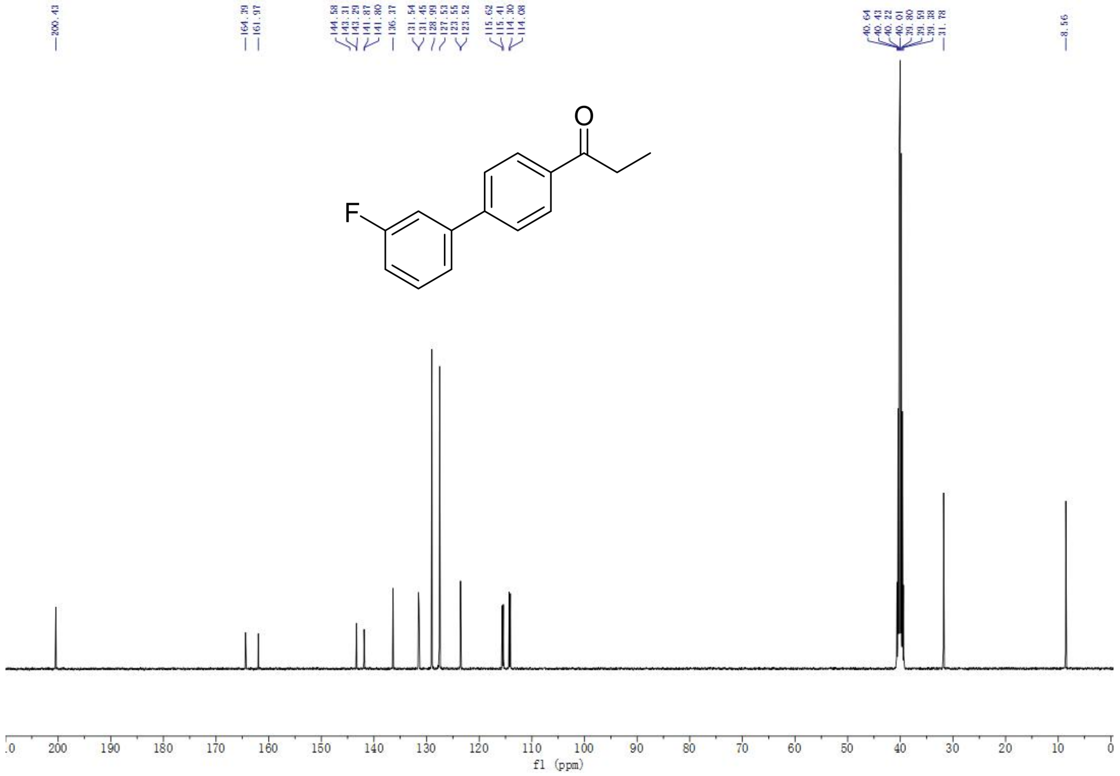

^{13}C-NMR spectrum of A4

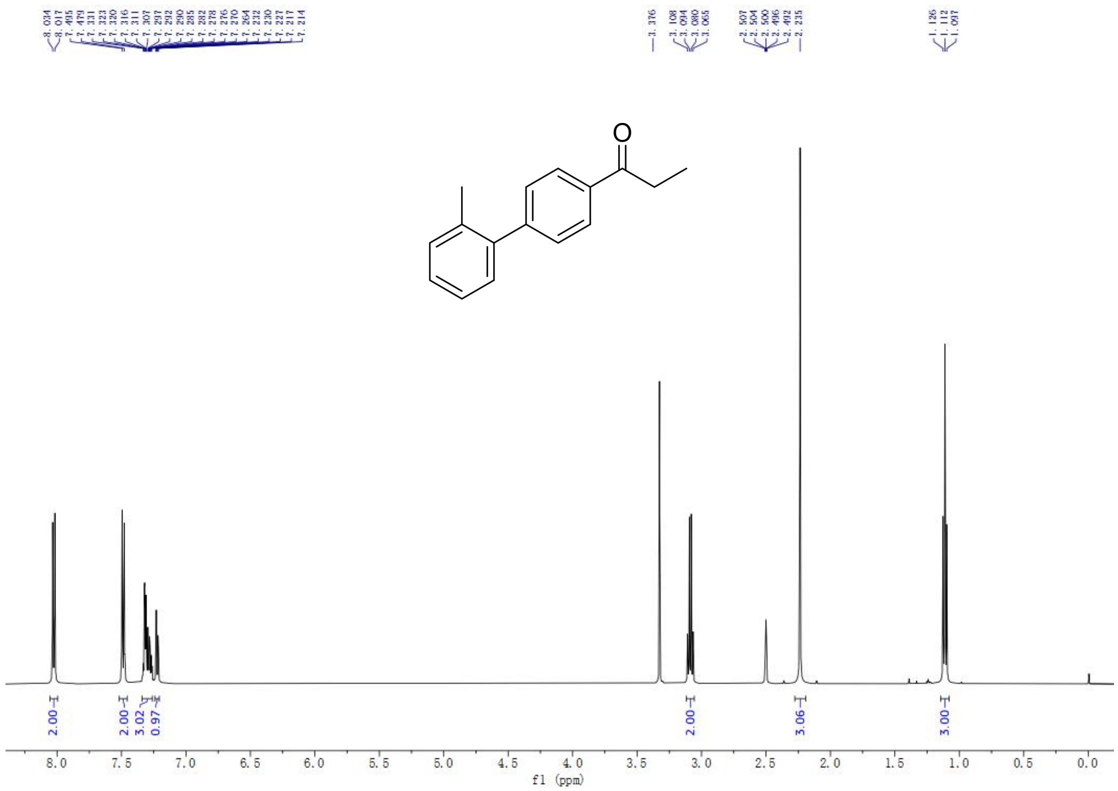

^{1}H-NMR spectrum of A6

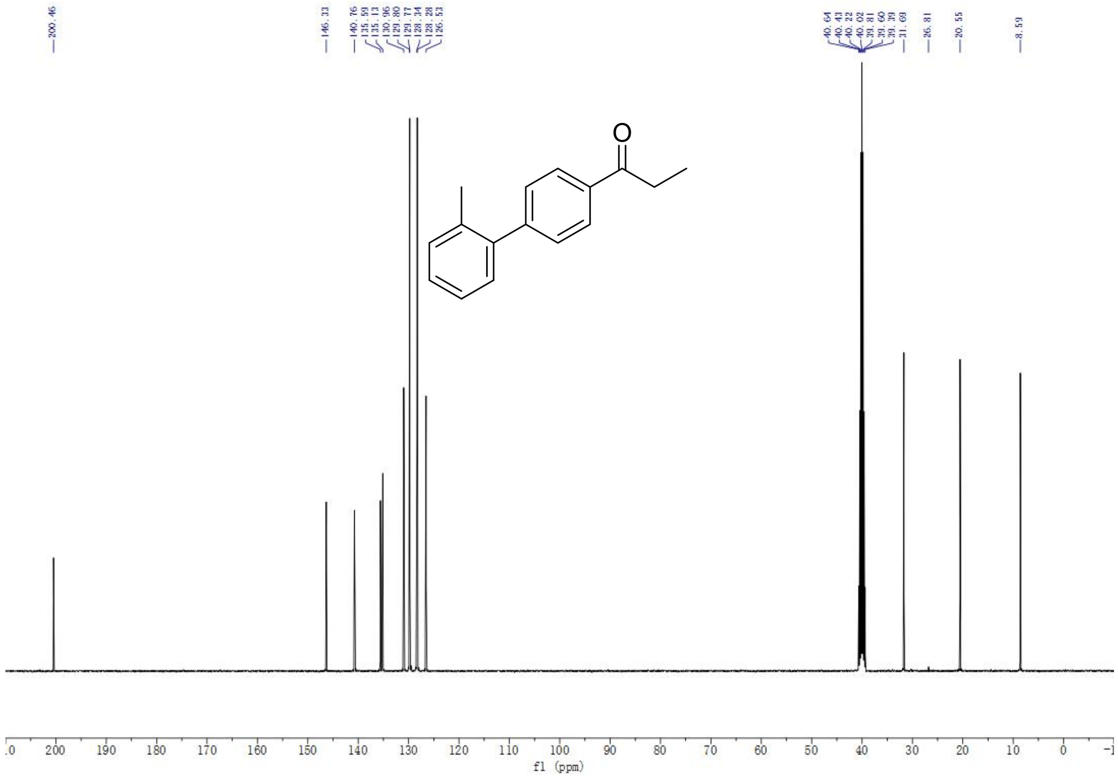

^{13}C-NMR spectrum of A6

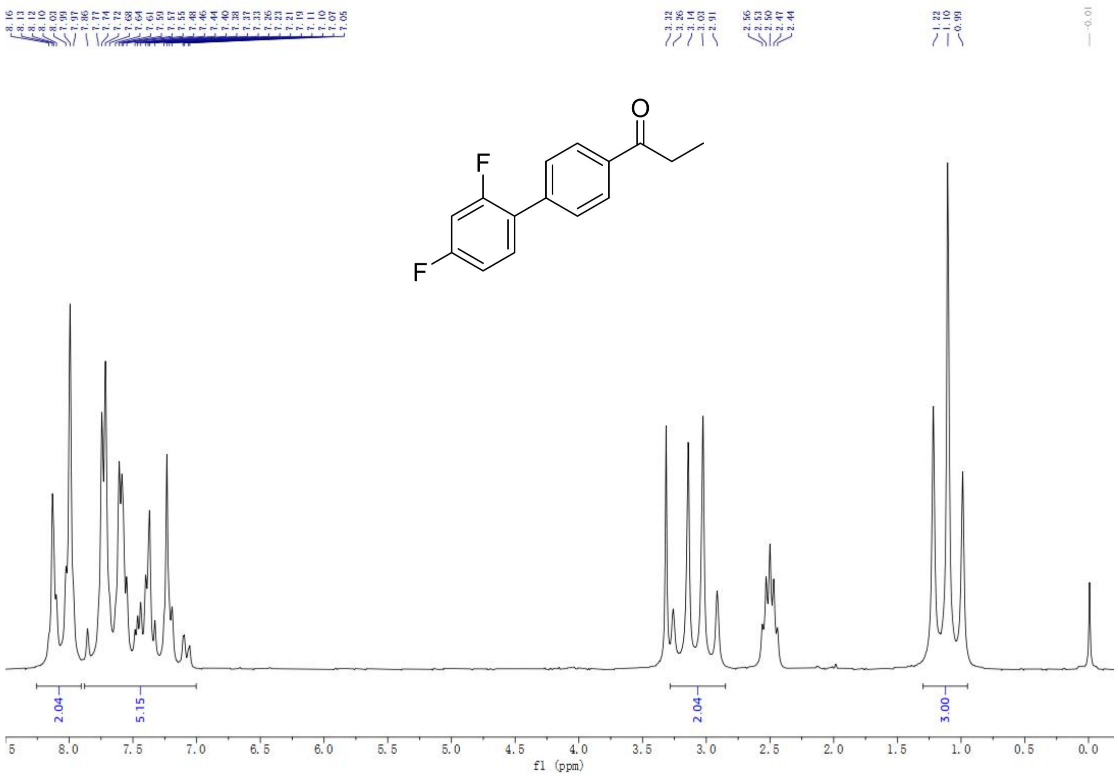

^{1}H-NMR spectrum of A8

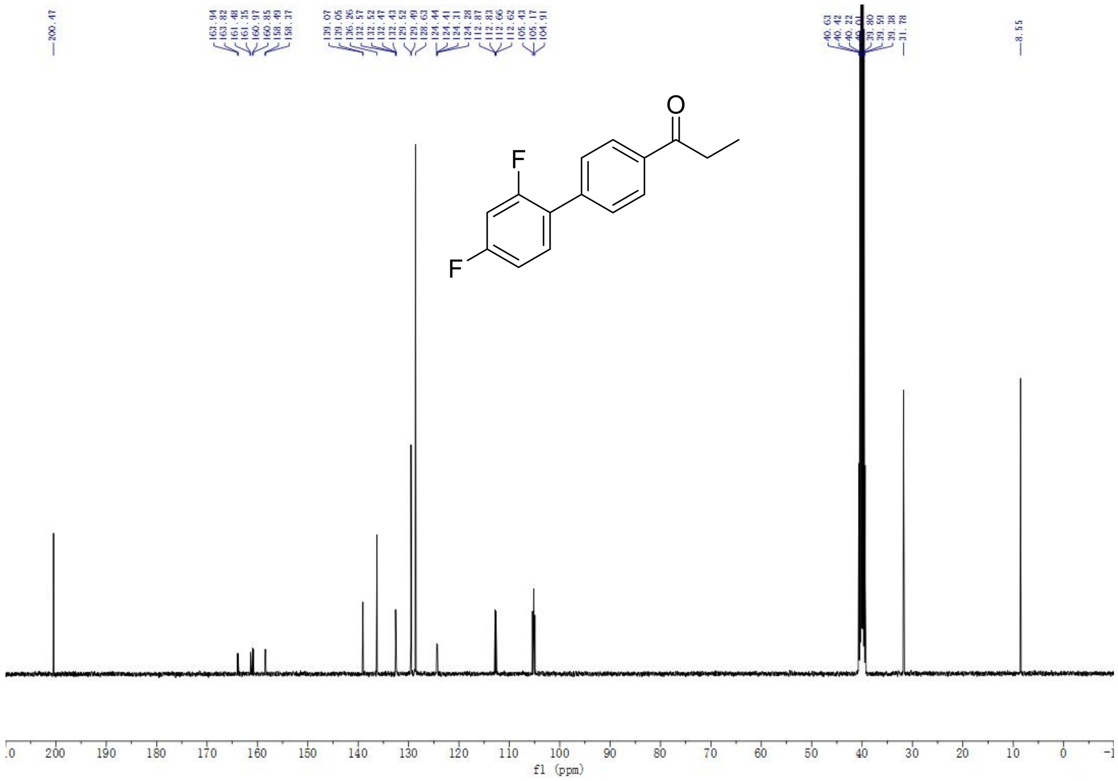

^{13}C-NMR spectrum of A8

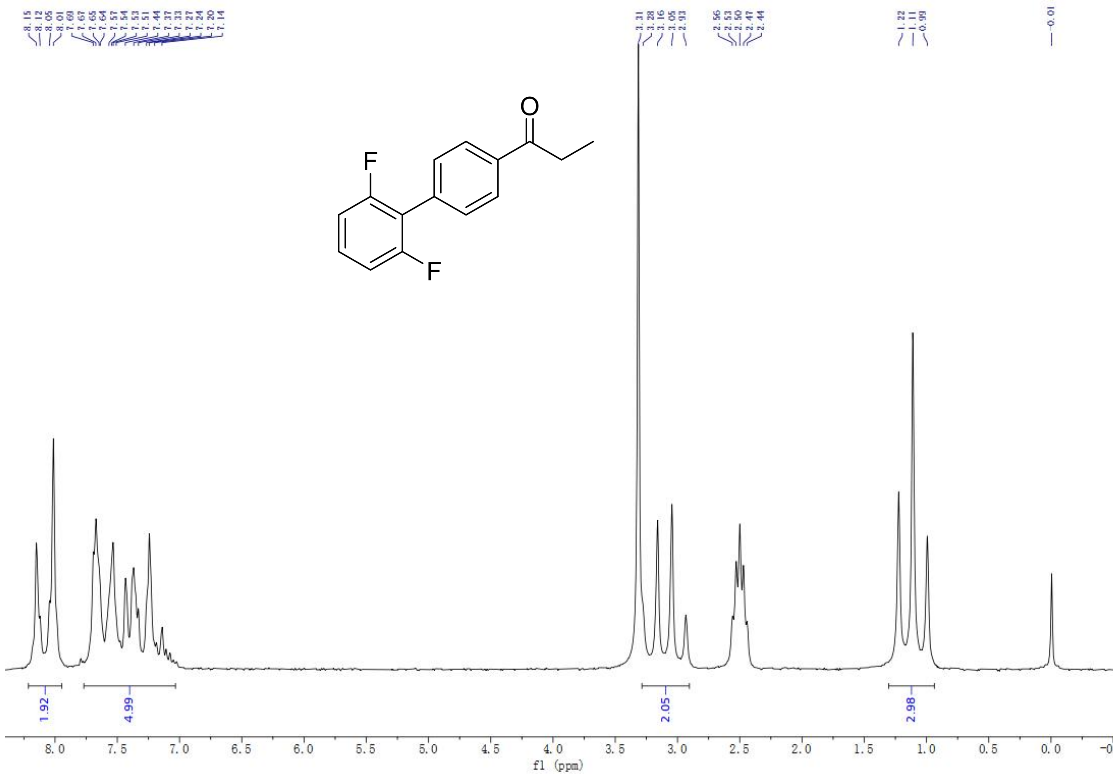

^{1}H-NMR spectrum of A11

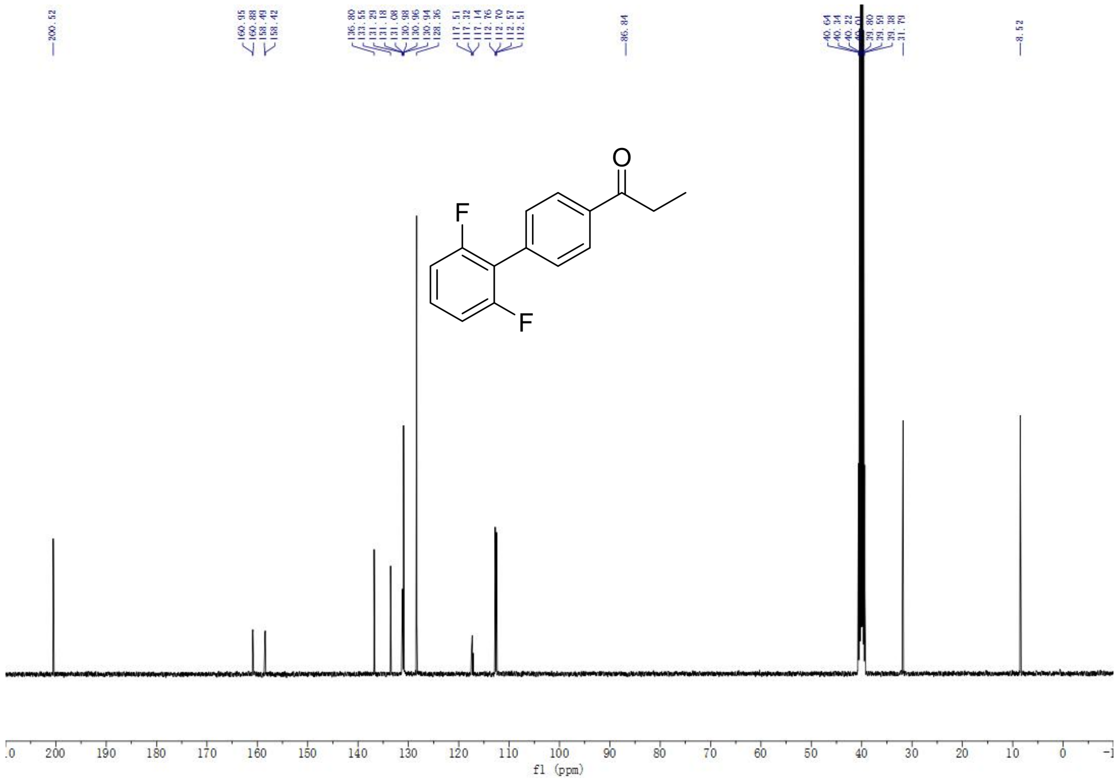

^{13}C-NMR spectrum of A11

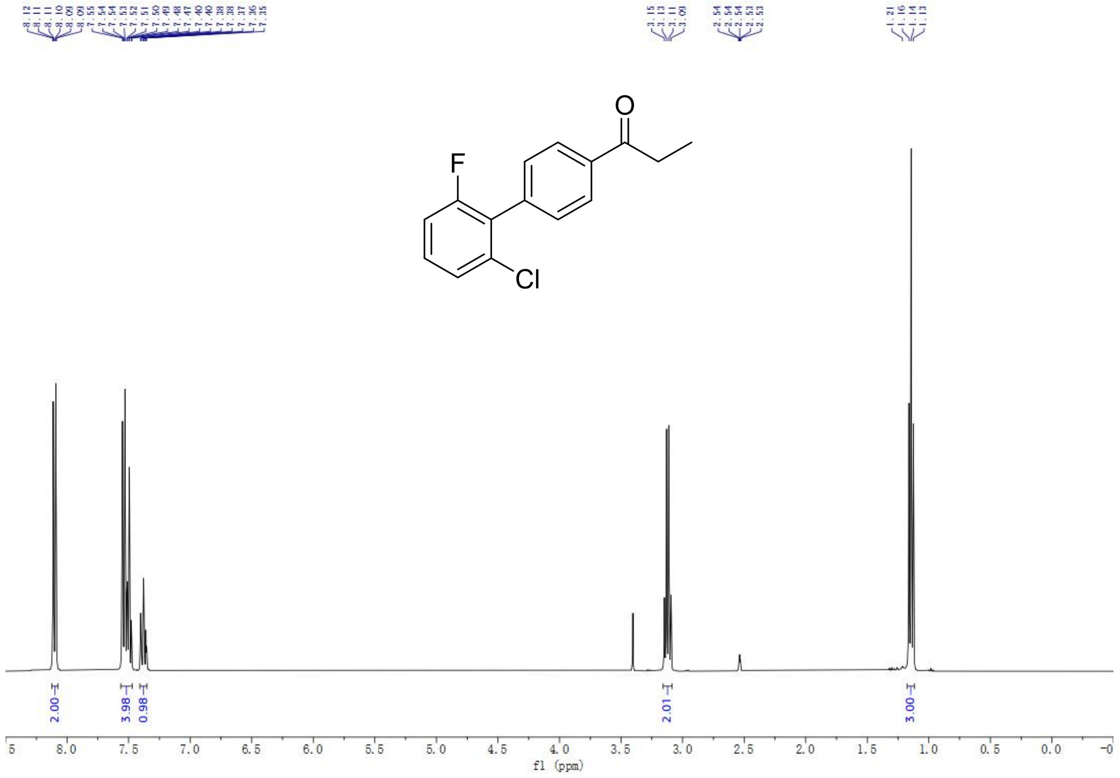

^{1}H-NMR spectrum of A12

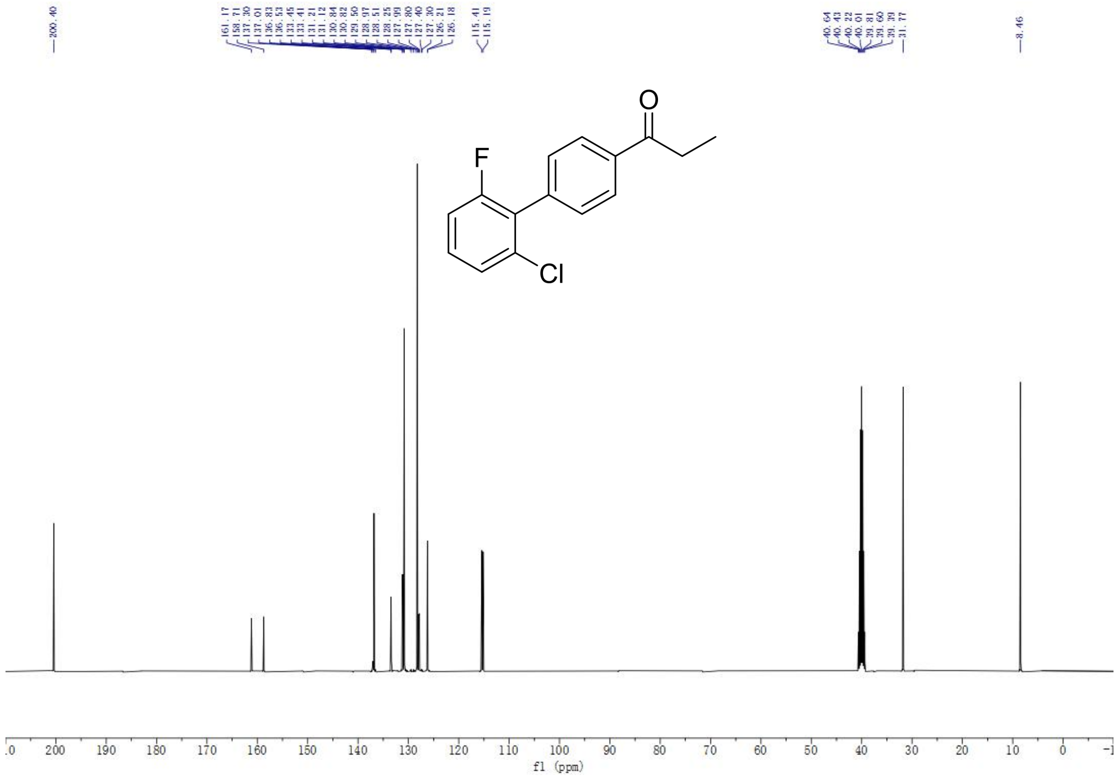

^{13}C-NMR spectrum of A12

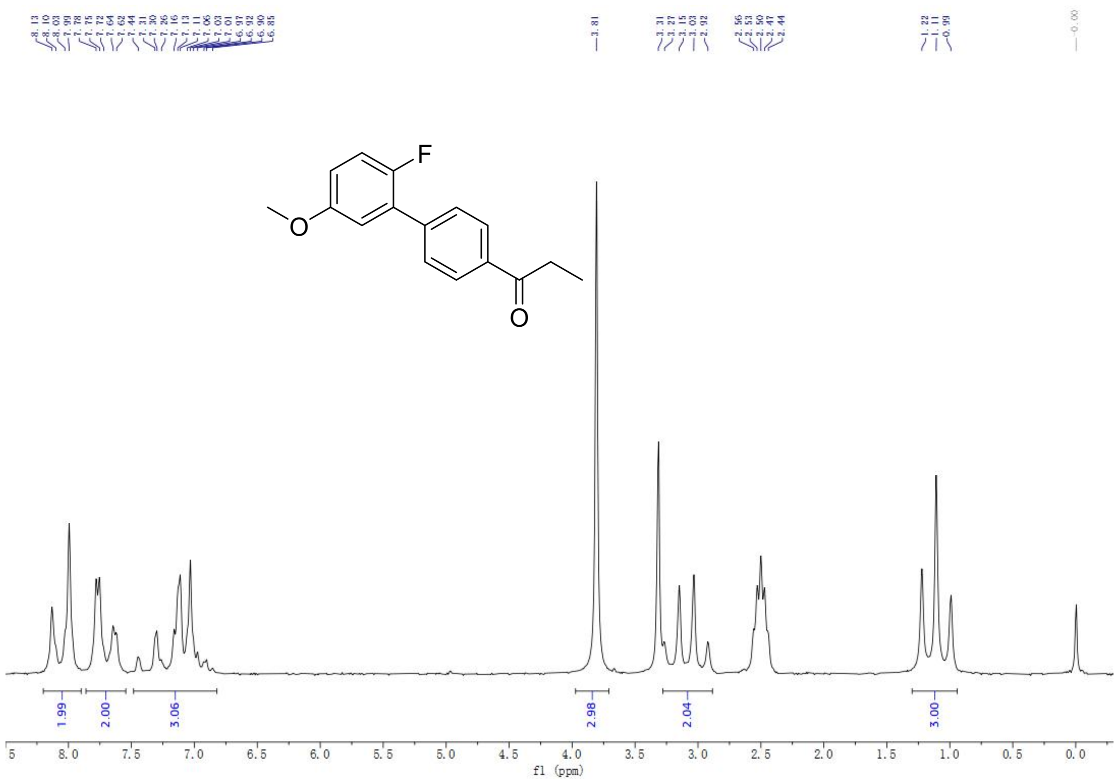

^{1}H-NMR spectrum of A14

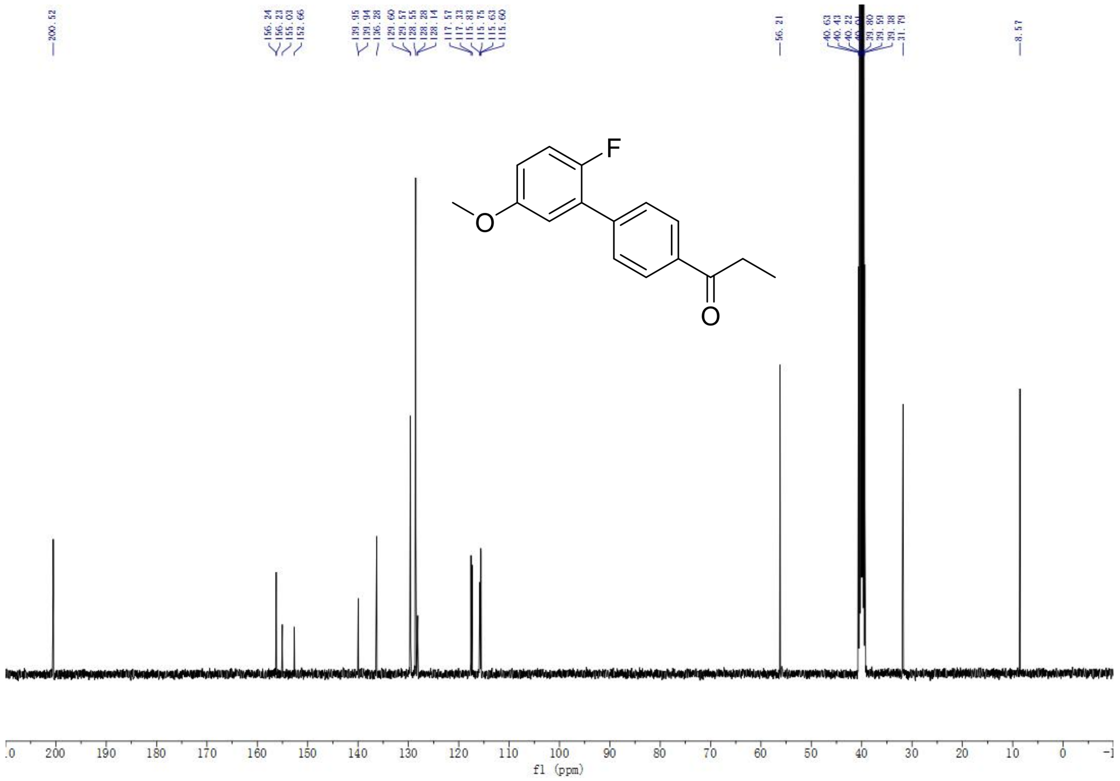

^{13}C-NMR spectrum of A14

HRMS Spectrum

Monoisotopic Mass, Even Electron Ions
1 formula(e) evaluated with 1 results within limits (up to 50 closest results for each mass)
Elements Used:
C: 23-23 H: 15-15 N: 1-1 O: 2-2 F: 3-3

AA2 6 (0.077)
1: TOF MS ES+

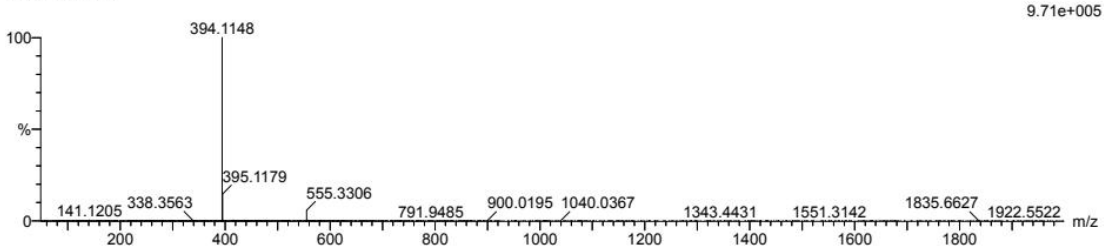

	Calc. Mass	mDa	PPM	DBE	i-FIT	Norm	Conf(%)	Formula
Minimum:				-1.5				
Maximum:		5.0	50.0	50.0				

Mass	Calc. Mass	mDa	PPM	DBE	i-FIT	Norm	Conf(%)	Formula
394.1148	394.1055	9.3	23.6	15.5	97.8	n/a	n/a	C23 H15 N O2 F3

HRMS Spectrum of AA2

Monoisotopic Mass, Even Electron Ions
1 formula(e) evaluated with 1 results within limits (up to 50 closest results for each mass)
Elements Used:
C: 23-23 H: 16-16 N: 1-1 O: 2-2 F: 1-1 Cl: 1-1
AA3 6 (0.076)
1: TOF MS ES+

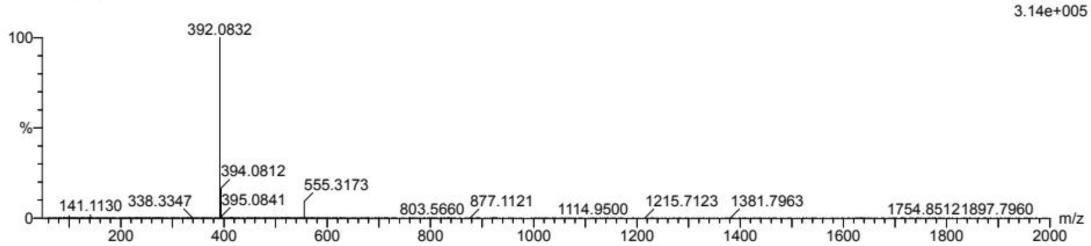

	Calc. Mass	mDa	PPM	DBE	i-FIT	Norm	Conf(%)	Formula
Minimum:				-1.5				
Maximum:		5.0	50.0	50.0				

Mass	Calc. Mass	mDa	PPM	DBE	i-FIT	Norm	Conf(%)	Formula
392.0832	392.0854	-2.2	-5.6	15.5	96.6	n/a	n/a	C23 H16 N O2 F Cl

HRMS Spectrum of AA3

Monoisotopic Mass, Even Electron Ions
1 formula(e) evaluated with 1 results within limits (up to 50 closest results for each mass)
Elements Used:
C: 24-24 H: 19-19 N: 1-1 O: 3-3 Cl: 1-1
BA3 6 (0.076)
1: TOF MS ES+

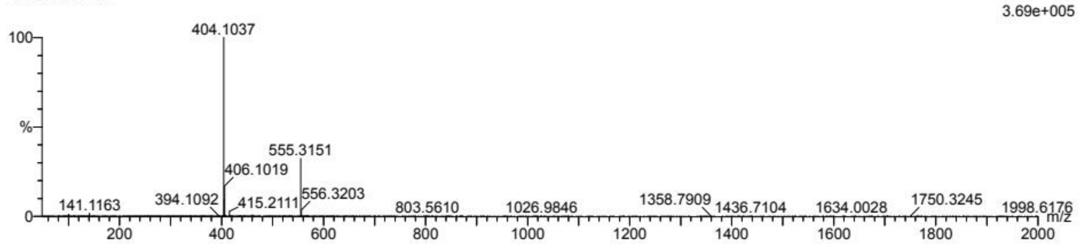

	Calc. Mass	mDa	PPM	DBE	i-FIT	Norm	Conf(%)	Formula
Minimum:				-1.5				
Maximum:		5.0	50.0	50.0				

Mass	Calc. Mass	mDa	PPM	DBE	i-FIT	Norm	Conf(%)	Formula
404.1037	404.1053	-1.6	-4.0	15.5	89.0	n/a	n/a	C24 H19 N O3 Cl

HRMS Spectrum of BA3

Monoisotopic Mass, Even Electron Ions
1 formula(e) evaluated with 1 results within limits (up to 50 closest results for each mass)
Elements Used:
C: 24-24 H: 19-19 N: 1-1 O: 3-3 F: 1-1

BA4 6 (0.076)
1: TOF MS ES+

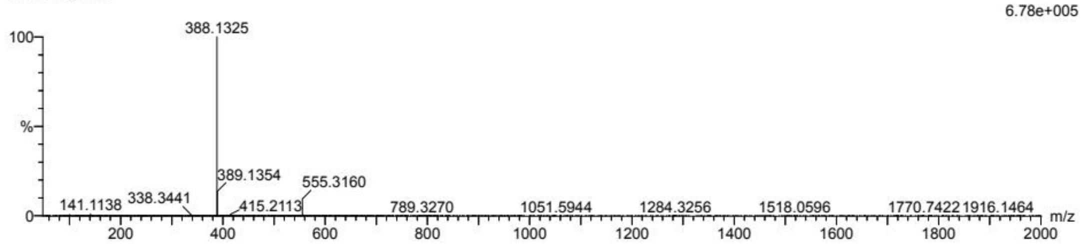

Minimum:				-1.5				
Maximum:		5.0	50.0	50.0				

Mass	Calc. Mass	mDa	PPM	DBE	i-FIT	Norm	Conf(%)	Formula
388.1325	388.1349	-2.4	-6.2	15.5	166.1	n/a	n/a	C24 H19 N O3 F

HRMS Spectrum of BA4

Monoisotopic Mass, Even Electron Ions
1 formula(e) evaluated with 1 results within limits (up to 50 closest results for each mass)
Elements Used:
C: 24-24 H: 18-18 N: 1-1 O: 2-2 F: 2-2
CA2 6 (0.076)
1: TOF MS ES+

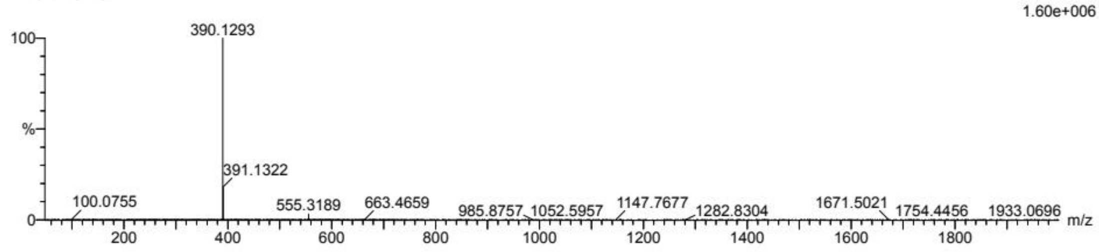

Minimum:				-1.5				
Maximum:		5.0	50.0	50.0				

Mass	Calc. Mass	mDa	PPM	DBE	i-FIT	Norm	Conf(%)	Formula
390.1293	390.1306	-1.3	-3.3	15.5	153.0	n/a	n/a	C24 H18 N O2 F2

HRMS Spectrum of CA2

Monoisotopic Mass, Even Electron Ions
1 formula(e) evaluated with 1 results within limits (up to 50 closest results for each mass)
Elements Used:
C: 24-24 H: 19-19 N: 1-1 O: 2-2 F: 1-1
CA4 6 (0.077)
1: TOF MS ES+

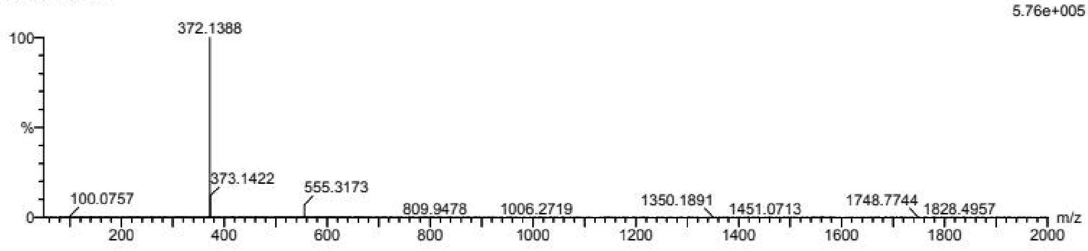

Minimum:				-1.5				
Maximum:		5.0	50.0	50.0				

Mass	Calc. Mass	mDa	PPM	DBE	i-FIT	Norm	Conf(%)	Formula
372.1388	372.1400	-1.2	-3.2	15.5	124.5	n/a	n/a	C24 H19 N O2 F

HRMS Spectrum of CA4

Monoisotopic Mass, Even Electron Ions
1 formula(e) evaluated with 1 results within limits (up to 50 closest results for each mass)
Elements Used:
C: 24-24 H: 18-18 N: 1-1 O: 2-2 F: 2-2
CA8 6 (0.076)
1: TOF MS ES+

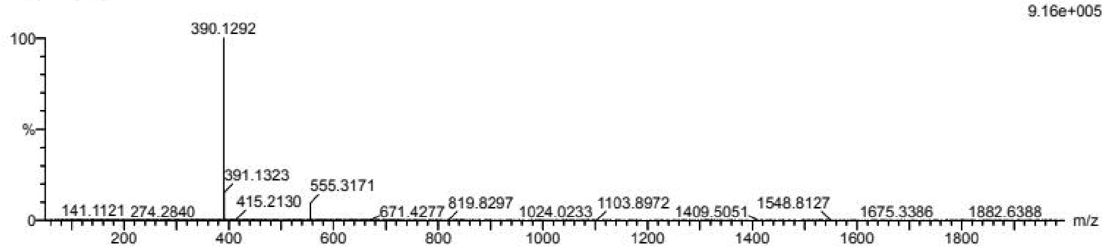

Minimum:				-1.5				
Maximum:		5.0	50.0	50.0				

Mass	Calc. Mass	mDa	PPM	DBE	i-FIT	Norm	Conf(%)	Formula
390.1292	390.1306	-1.4	-3.6	15.5	145.8	n/a	n/a	C24 H18 N O2 F2

HRMS Spectrum of CA8

Monoisotopic Mass, Even Electron Ions
7 formula(e) evaluated with 1 results within limits (up to 50 best isotopic matches for each mass)
Elements Used:
C: 23-23 H: 13-13 N: 1-1 O: 2-2 F: 2-2 S: 0-6 Cl: 1-1
EA2-0914 12 (0.088)
1: TOF MS ES-

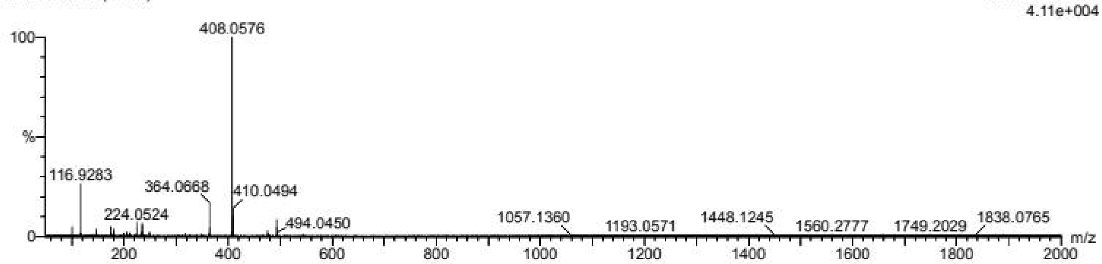

Minimum:				-1.5				
Maximum:		5.0	10.0	50.0				

Mass	Calc. Mass	mDa	PPM	DBE	i-FIT	Norm	Conf(%)	Formula
408.0576	408.0603	-2.7	-6.6	16.5	377.2	n/a	n/a	C23 H13 N O2 F2 Cl

HRMS Spectrum of EA2

Monoisotopic Mass, Even Electron Ions
1 formula(e) evaluated with 1 results within limits (up to 50 best isotopic matches for each mass)
Elements Used:
C: 23-23 H: 14-14 N: 1-1 O: 2-2 Cl: 2-2
EA3-0914 13 (0.093)
1: TOF MS ES-

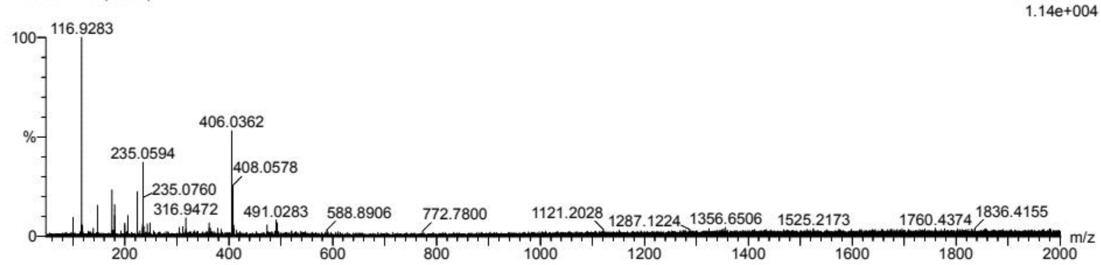

Minimum:				-1.5				
Maximum:		5.0	10.0	50.0				

Mass	Calc. Mass	mDa	PPM	DBE	i-FIT	Norm	Conf(%)	Formula
406.0362	406.0402	-4.0	-9.9	16.5	389.5	n/a	n/a	C23 H14 N O2 Cl2

HRMS Spectrum of EA3

Monoisotopic Mass, Even Electron Ions
1 formula(e) evaluated with 1 results within limits (up to 50 closest results for each mass)
Elements Used:
C: 23-23 H: 16-16 N: 1-1 O: 2-2 F: 1-1 Cl: 1-1
EA4 7 (0.085)
1: TOF MS ES+

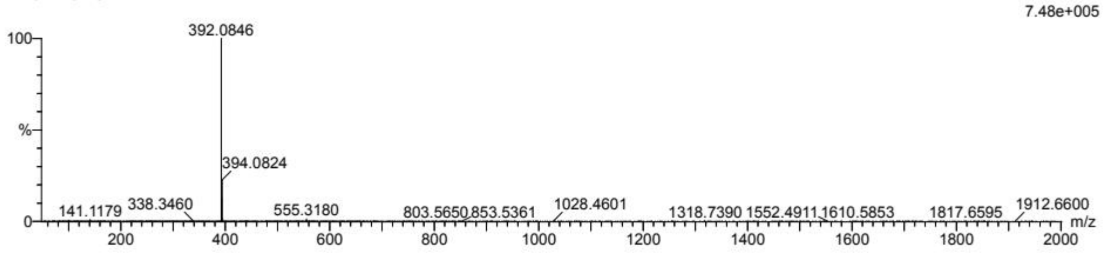

	Calc. Mass	mDa	PPM	DBE	i-FIT	Norm	Conf(%)	Formula
Minimum:				-1.5				
Maximum:		5.0	50.0	50.0				

Mass	Calc. Mass	mDa	PPM	DBE	i-FIT	Norm	Conf(%)	Formula
392.0846	392.0854	-0.8	-2.0	15.5	131.9	n/a	n/a	C23 H16 N O2 F Cl

HRMS Spectrum of EA4

Monoisotopic Mass, Even Electron Ions
1 formula(e) evaluated with 1 results within limits (up to 50 best isotopic matches for each mass)
Elements Used:
C: 24-24 H: 17-17 N: 1-1 O: 2-2 Cl: 1-1
EA6-0914 11 (0.083) 1: TOF MS ES-

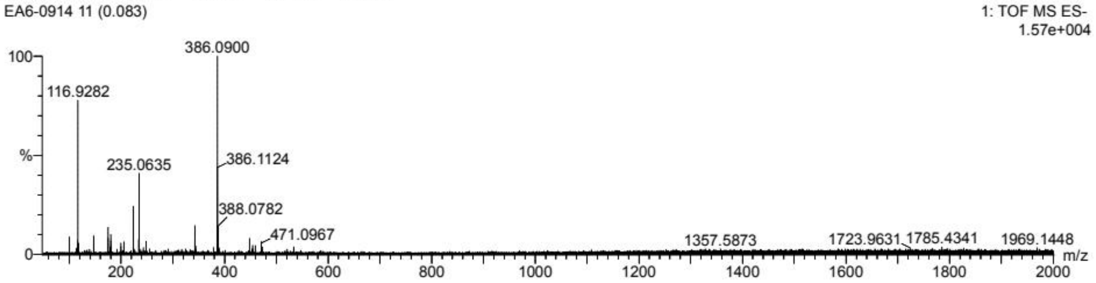

	Calc. Mass	mDa	PPM	DBE	i-FIT	Norm	Conf(%)	Formula
Minimum:				-1.5				
Maximum:		5.0	10.0	50.0				

Mass	Calc. Mass	mDa	PPM	DBE	i-FIT	Norm	Conf(%)	Formula
386.0900	386.0948	-4.8	-12.4	16.5	417.8	n/a	n/a	C24 H17 N O2 Cl

HRMS Spectrum of EA6

Monoisotopic Mass, Even Electron Ions
1 formula(e) evaluated with 1 results within limits (up to 50 closest results for each mass)
Elements Used:
C: 23-23 H: 15-15 N: 1-1 O: 2-2 F: 2-2 Cl: 1-1
EA8 7 (0.085)
1: TOF MS ES+

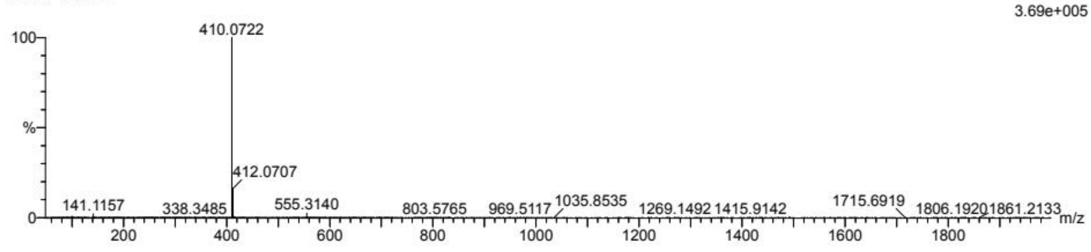

	Calc. Mass	mDa	PPM	DBE	i-FIT	Norm	Conf(%)	Formula
Minimum:				-1.5				
Maximum:		5.0	50.0	50.0				

Mass	Calc. Mass	mDa	PPM	DBE	i-FIT	Norm	Conf(%)	Formula
410.0722	410.0759	-3.7	-9.0	15.5	96.0	n/a	n/a	C23 H15 N O2 F2 Cl

HRMS Spectrum of EA8

Monoisotopic Mass, Even Electron Ions
1 formula(e) evaluated with 1 results within limits (up to 50 closest results for each mass)
Elements Used:
C: 24-24 H: 18-18 N: 1-1 O: 3-3 F: 2-2
FA11 5 (0.068)
1: TOF MS ES+

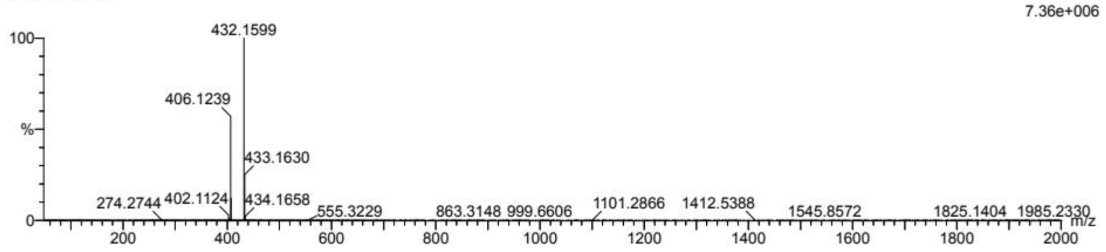

Minimum:				-1.5				
Maximum:		5.0	50.0	50.0				
Mass	Calc. Mass	mDa	PPM	DBE	i-FIT	Norm	Conf(%)	Formula
406.1239	406.1255	-1.6	-3.9	15.5	195.4	n/a	n/a	C24 H18 N O3 F2

HRMS Spectrum of FA11

Monoisotopic Mass, Even Electron Ions
1 formula(e) evaluated with 1 results within limits (up to 50 closest results for each mass)
Elements Used:
C: 24-24 H: 18-18 N: 1-1 O: 3-3 F: 1-1 Cl: 1-1
FA12 5 (0.068)
1: TOF MS ES+

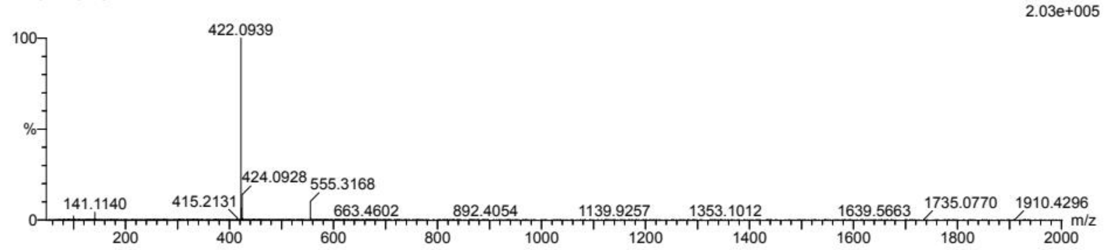

Minimum:				-1.5				
Maximum:		5.0	50.0	50.0				
Mass	Calc. Mass	mDa	PPM	DBE	i-FIT	Norm	Conf(%)	Formula
422.0939	422.0959	-2.0	-4.7	15.5	97.4	n/a	n/a	C24 H18 N O3 F Cl

HRMS Spectrum of FA12

Monoisotopic Mass, Even Electron Ions
1 formula(e) evaluated with 1 results within limits (up to 50 closest results for each mass)
Elements Used:
C: 25-25 H: 21-21 N: 1-1 O: 4-4 F: 1-1
FA14 5 (0.068)
1: TOF MS ES+

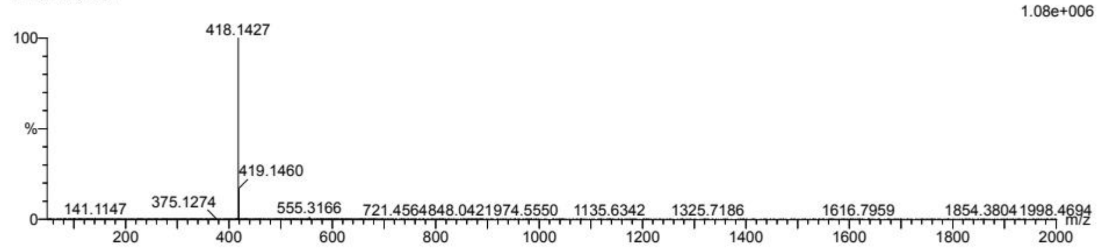

Minimum:				-1.5				
Maximum:		5.0	50.0	50.0				
Mass	Calc. Mass	mDa	PPM	DBE	i-FIT	Norm	Conf(%)	Formula
418.1427	418.1455	-2.8	-6.7	15.5	161.3	n/a	n/a	C25 H21 N O4 F

HRMS Spectrum of FA14

Monoisotopic Mass, Even Electron Ions
1 formula(e) evaluated with 1 results within limits (up to 50 closest results for each mass)
Elements Used:
C: 15-15 H: 13-13 O: 1-1 F: 2-2
H-A2 7 (0.085)
1: TOF MS ES+

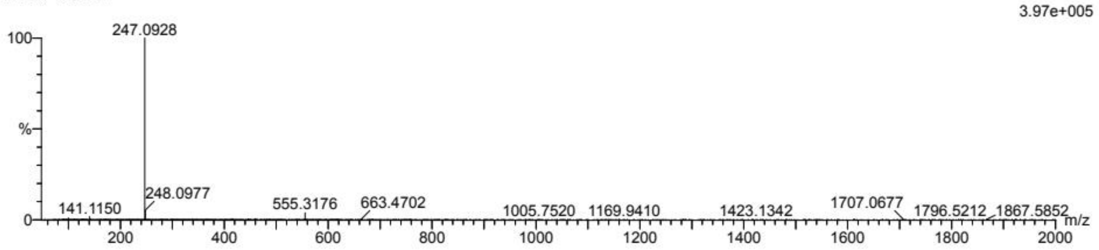

Minimum:				-1.5
Maximum:		5.0	50.0	50.0

Mass	Calc. Mass	mDa	PPM	DBE	i-FIT	Norm	Conf(%)	Formula
247.0928	247.0934	-0.6	-2.4	8.5	146.0	n/a	n/a	C15 H13 O F2

HRMS Spectrum of A2

Monoisotopic Mass, Even Electron Ions
1 formula(e) evaluated with 1 results within limits (up to 50 closest results for each mass)
Elements Used:
C: 15-15 H: 14-14 O: 1-1 Cl: 1-1
H-A3 7 (0.085)
1: TOF MS ES+

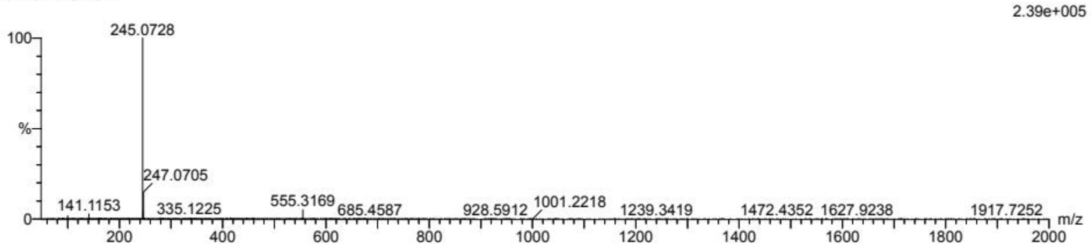

Minimum:				-1.5
Maximum:		5.0	50.0	50.0

Mass	Calc. Mass	mDa	PPM	DBE	i-FIT	Norm	Conf(%)	Formula
245.0728	245.0733	-0.5	-2.0	8.5	81.1	n/a	n/a	C15 H14 O Cl

HRMS Spectrum of A3

Monoisotopic Mass, Even Electron Ions
1 formula(e) evaluated with 1 results within limits (up to 50 best isotopic matches for each mass)
Elements Used:
C: 15-15 H: 14-14 O: 1-1 F: 1-1

H-A4 7 (0.085)
1: TOF MS ES+

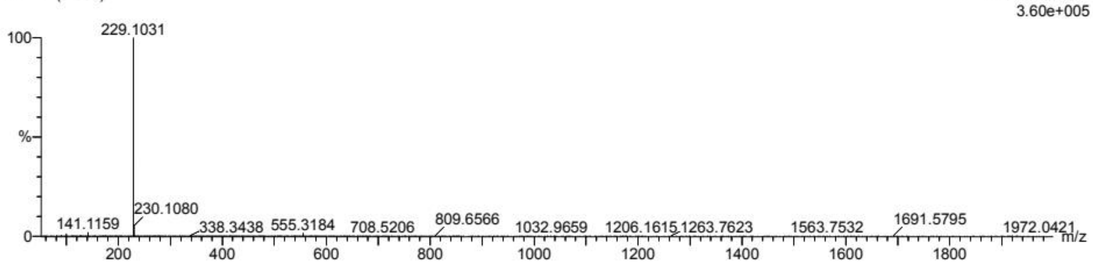

Minimum:				-1.5
Maximum:		5.0	10.0	50.0

Mass	Calc. Mass	mDa	PPM	DBE	i-FIT	Norm	Conf(%)	Formula
229.1031	229.1029	0.2	0.9	8.5	115.9	n/a	n/a	C15 H14 O F

HRMS Spectrum of A4

Monoisotopic Mass, Even Electron Ions
1 formula(e) evaluated with 1 results within limits (up to 50 closest results for each mass)
Elements Used:
C: 16-16 H: 17-17 O: 1-1
H-A6 7 (0.085)
1: TOF MS ES+

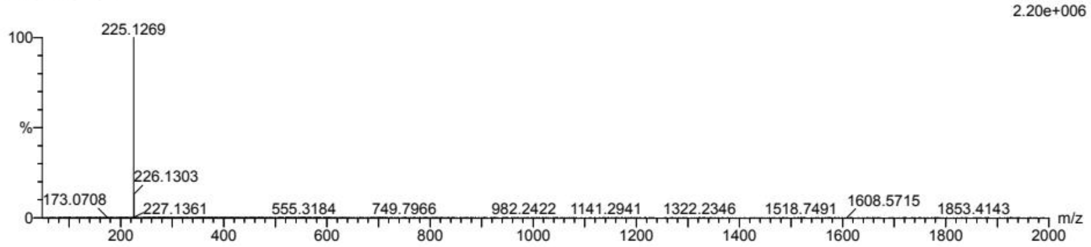

Minimum:				-1.5				
Maximum:		5.0	50.0	50.0				
Mass	Calc. Mass	mDa	PPM	DBE	i-FIT	Norm	Conf(%)	Formula
225.1269	225.1279	-1.0	-4.4	8.5	126.4	n/a	n/a	C16 H17 O

HRMS Spectrum of A6

Monoisotopic Mass, Even Electron Ions
1 formula(e) evaluated with 1 results within limits (up to 50 closest results for each mass)
Elements Used:
C: 15-15 H: 13-13 O: 1-1 F: 2-2

H-A8 7 (0.085)
1: TOF MS ES+

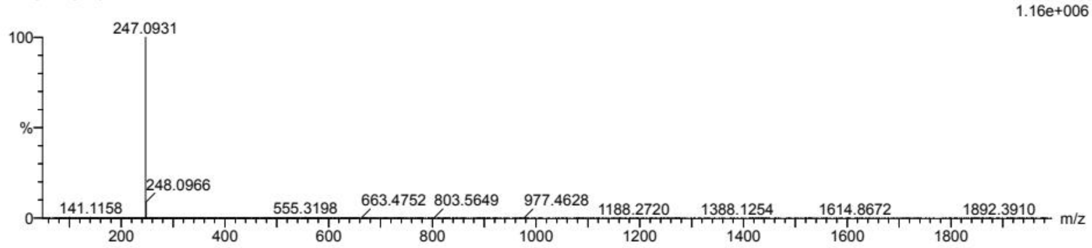

Minimum:				-1.5				
Maximum:		5.0	50.0	50.0				
Mass	Calc. Mass	mDa	PPM	DBE	i-FIT	Norm	Conf(%)	Formula
247.0931	247.0934	-0.3	-1.2	8.5	162.2	n/a	n/a	C15 H13 O F2

HRMS Spectrum of A8

Monoisotopic Mass, Even Electron Ions
1 formula(e) evaluated with 1 results within limits (up to 50 closest results for each mass)
Elements Used:
C: 15-15 H: 13-13 O: 1-1 F: 2-2
H-A11 6 (0.077)
1: TOF MS ES+

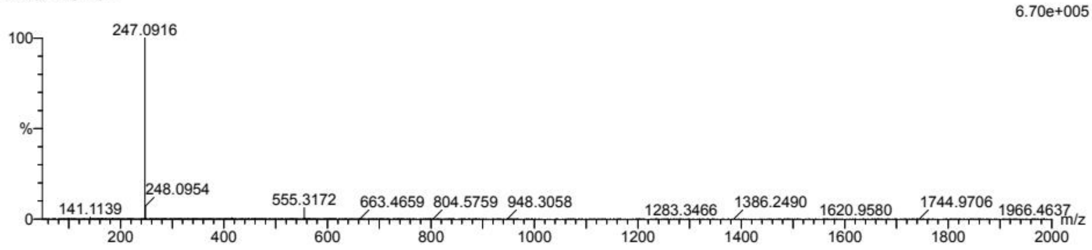

Minimum:				-1.5				
Maximum:		5.0	50.0	50.0				
Mass	Calc. Mass	mDa	PPM	DBE	i-FIT	Norm	Conf(%)	Formula
247.0916	247.0934	-1.8	-7.3	8.5	142.2	n/a	n/a	C15 H13 O F2

HRMS Spectrum of A11

Monoisotopic Mass, Even Electron Ions
1 formula(e) evaluated with 1 results within limits (up to 50 closest results for each mass)
Elements Used:
C: 15-15 H: 13-13 O: 1-1 F: 1-1 Cl: 1-1
H-A12 7 (0.085)
1: TOF MS ES+

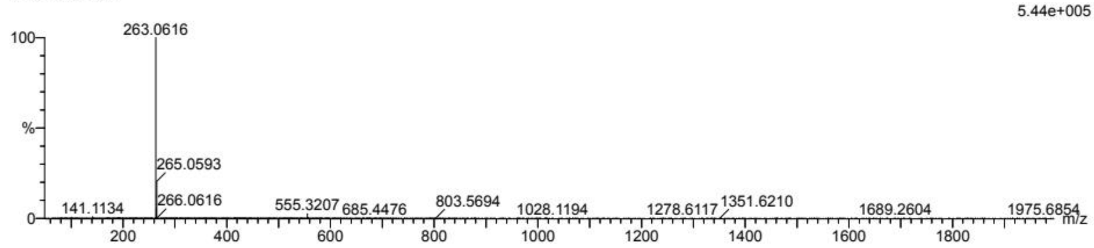

Minimum:				-1.5				
Maximum:		5.0	50.0	50.0				
Mass	Calc. Mass	mDa	PPM	DBE	i-FIT	Norm	Conf(%)	Formula
263.0616	263.0639	-2.3	-8.7	8.5	121.2	n/a	n/a	C15 H13 O F Cl

HRMS Spectrum of A12

Monoisotopic Mass, Even Electron Ions
1 formula(e) evaluated with 1 results within limits (up to 50 closest results for each mass)
Elements Used:
C: 16-16 H: 16-16 O: 2-2 F: 1-1
H-A14 6 (0.077)
1: TOF MS ES+

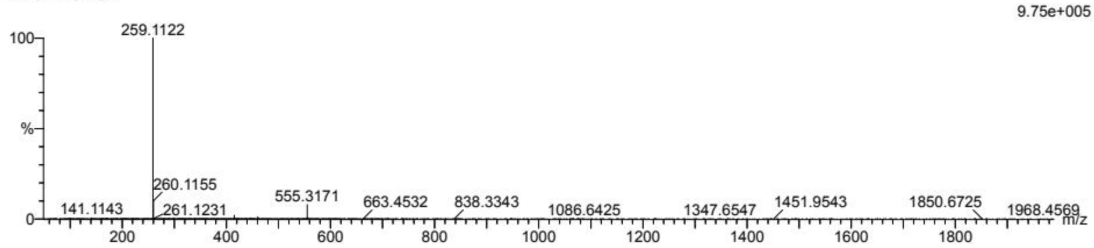

Minimum:				-1.5				
Maximum:		5.0	50.0	50.0				
Mass	Calc. Mass	mDa	PPM	DBE	i-FIT	Norm	Conf(%)	Formula
259.1122	259.1134	-1.2	-4.6	8.5	111.9	n/a	n/a	C16 H16 O2 F

HRMS Spectrum of A14

The original images of western blot

Full unedited gel for Fig. 4b

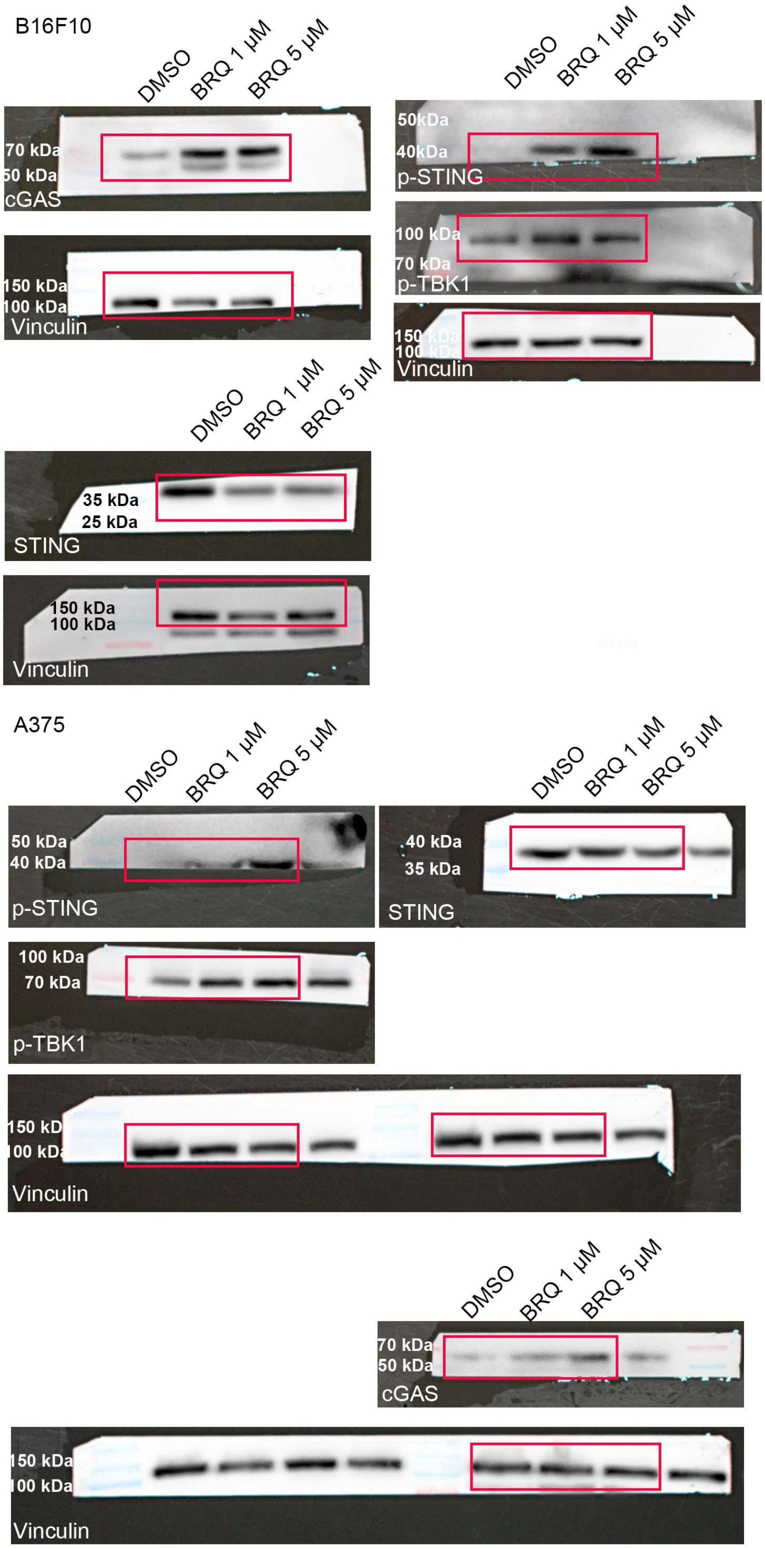

Full unedited gel for Fig. 4d

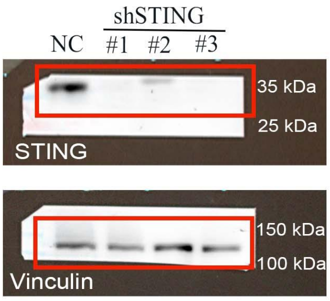

Full unedited gel for Fig. 5c

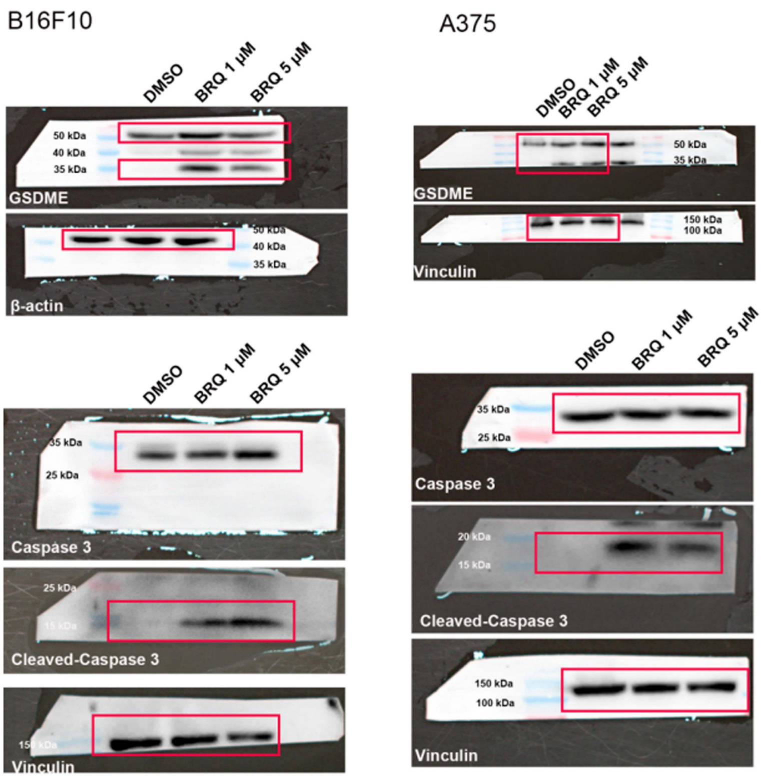

Full unedited gel for Fig. 5h

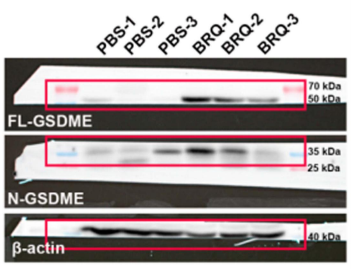

Full unedited gel for Fig. 5j

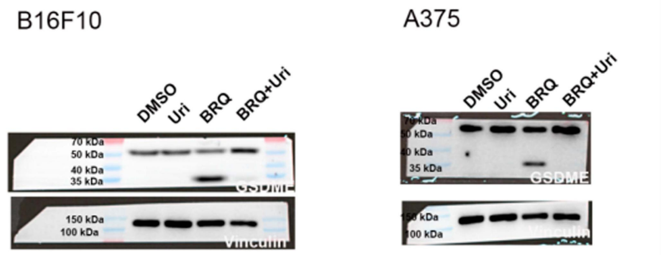

Full unedited gel for Fig. 5l

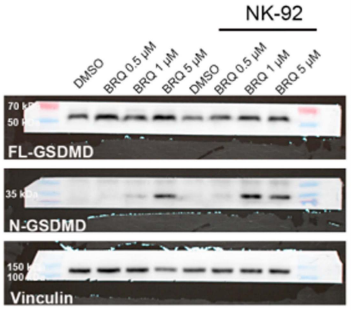

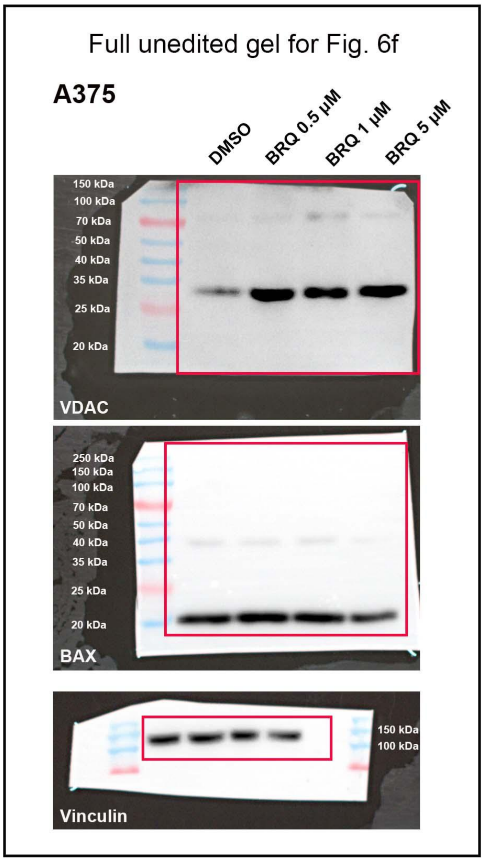
Full unedited gel for Fig. 6f
A375
DMSO
BRQ 0.5 µM
BRQ 1 µM
BRQ 5 µM
150 kDa
100 kDa
70 kDa
50 kDa
40 kDa
35 kDa
25 kDa
20 kDa
VDAC
250 kDa
150 kDa
100 kDa
70 kDa
50 kDa
40 kDa
35 kDa
25 kDa
20 kDa
BAX
150 kDa
100 kDa
Vinculin

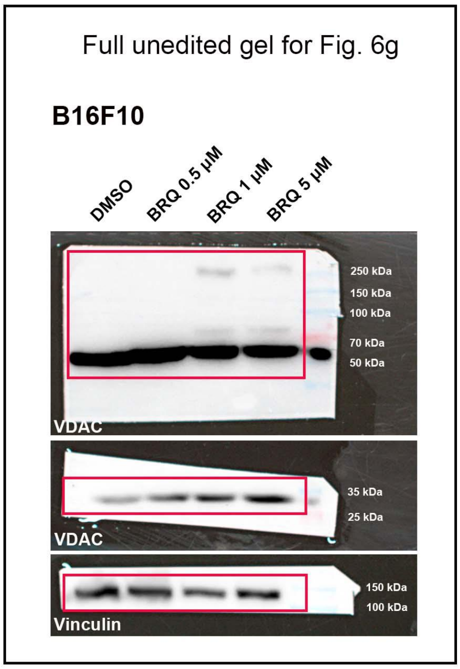
Full unedited gel for Fig. 6g
B16F10
DMSO
BRQ 0.5 µM
BRQ 1 µM
BRQ 5 µM
250 kDa
150 kDa
100 kDa
70 kDa
50 kDa
VDAC
35 kDa
25 kDa
VDAC
150 kDa
100 kDa
Vinculin

Full unedited gel for Fig. S3a

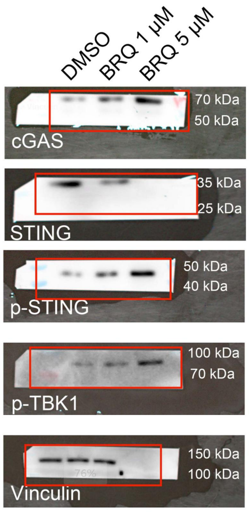

Full unedited gel for Fig. S3d

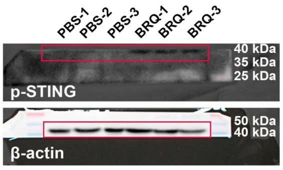

Full unedited gel for Fig. S3i

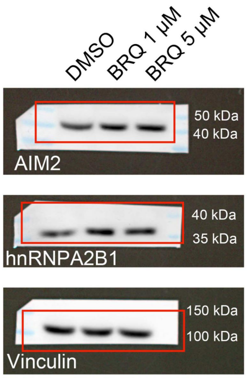

Full unedited gel for Fig. S3g

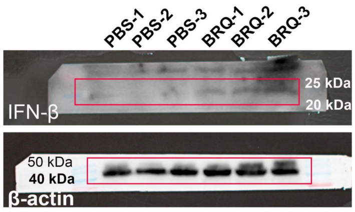

Full unedited gel for Fig. S3j

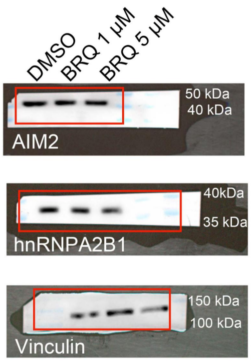

Full unedited gel for Fig. S3l

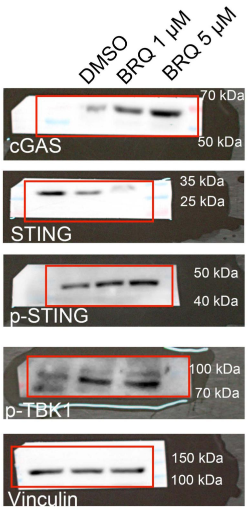

Full unedited gel for Fig. S4a

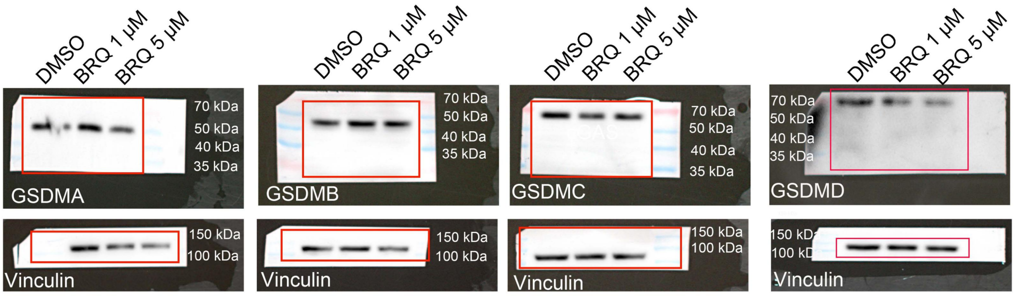

Full unedited gel for Fig. S4b

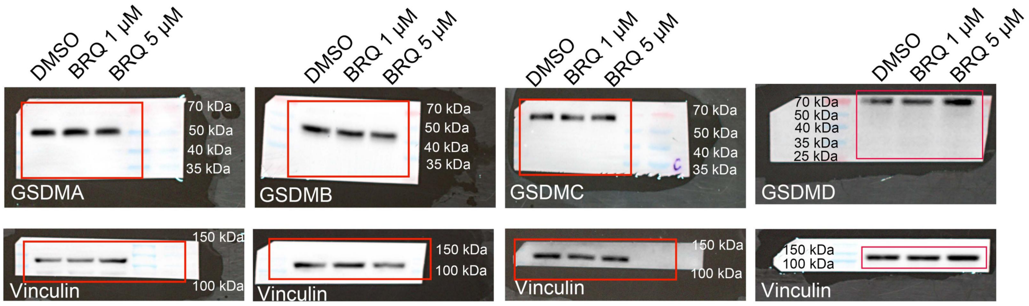

Full unedited gel for Fig. S4f

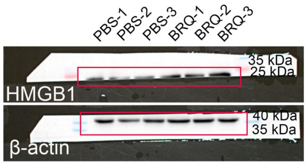

Full unedited gel for Fig. S4g

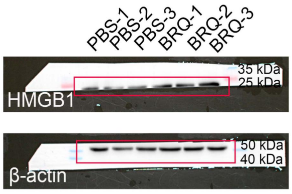

Full unedited gel for Fig. S4k

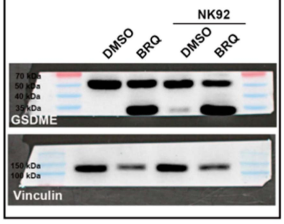

Full unedited gel for Fig. S4e

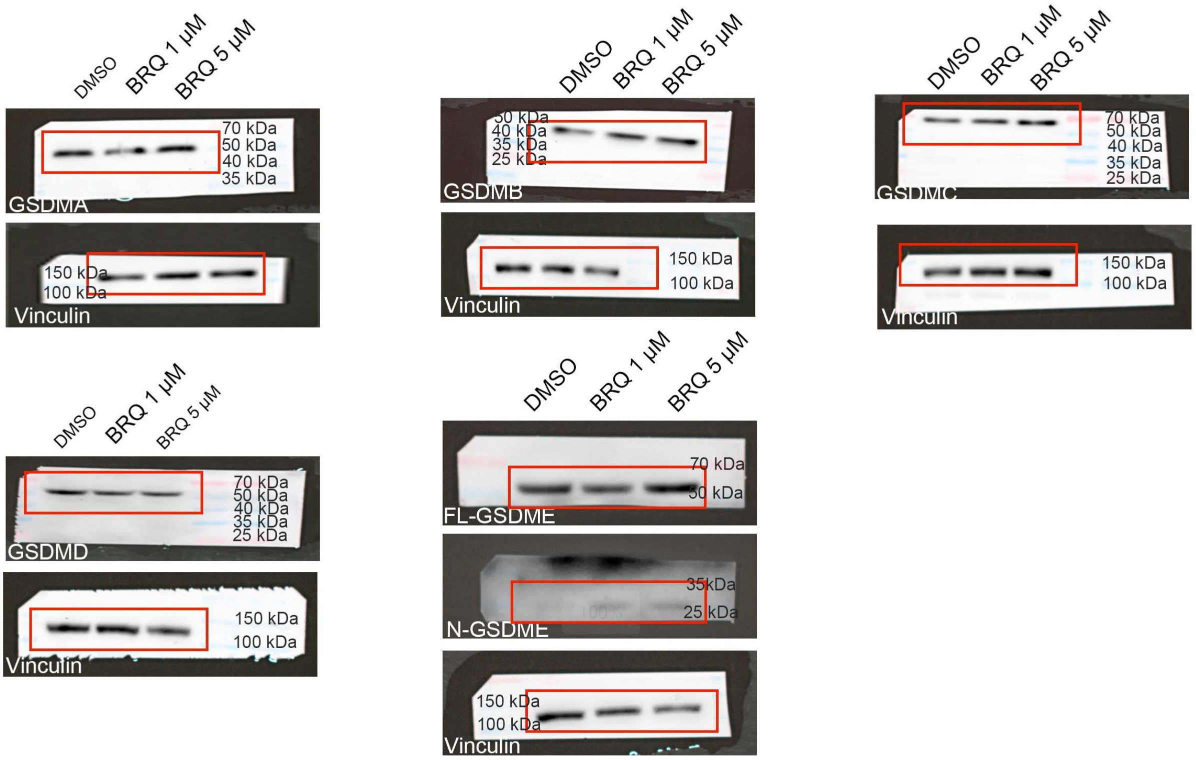

Full unedited gel for Fig. S5c

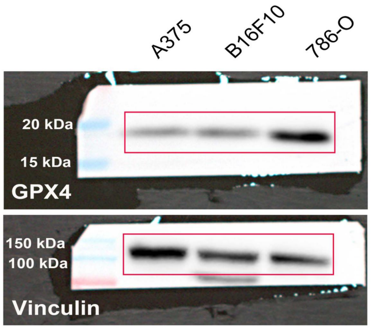

Full unedited gel for Fig. S5i

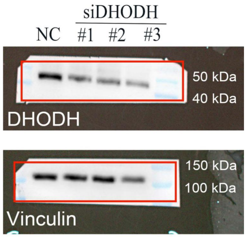

Full unedited gel for Fig. S5k

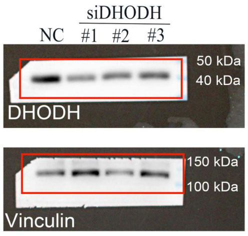

Supplement: Supplementary file 1 — Supplementary Material 1. [file 43556_2025_339_MOESM1_ESM.pdf]
